# AI-enabled clinical decision support in breast cancer care: a blinded multicenter benchmarking study comparing medically specialized with a general-purpose system

**DOI:** 10.1007/s10916-026-02434-w

**Published:** 2026-07-04

**Authors:** Jonas Freudenberg, Johannes Knitza, Niklas Gremke, Niklas Amann, Thomas M. Deutsch, Nikolas Tauber, Kerstin Muras, Zoe S. Oftring, Tobias Engler, Alexander Englisch, Stefan Lukac, Adriano Fabi, André S. Alves, Kristin Reinhardt, Markus Wallwiener, Moritz Kuhlmann, Jonathan Bamberger, Uwe Wagner, Sebastian Kuhn, Sebastian Griewing

**Affiliations:** 1 Philipps-Universität Marburg, School of Medicine, Institute for Digital Medicine, Marburg, Germany; 2 Philipps-Universität Marburg, School of Medicine, Clinic for Gynecology and Obstetrics, Marburg, Germany; 3 Erlangen University Breast Center, University Hospital Erlangen, Friedrich Alexander University of Erlangen-Nuremberg, Erlangen, Germany; 4 University Hospital Heidelberg, Heidelberg University Breast Center, University of Heidelberg, Heidelberg, Germany; 5 Luebeck University Breast Center, University Hospital Schleswig-Holstein, University of Luebeck, Campus Luebeck, Luebeck, Germany; 6 Tuebingen University Breast Center, University Hospital Tuebingen, Eberhard Karls University of Tuebingen, Tuebingen, Germany; 7 Department of Obstetrics and Gynecology, University Hospital Ulm, University of Ulm, Ulm, Germany; 8 Halle-Wittenberg University Breast Center, University Hospital Halle (Saale), Martin Luther University of Halle-Wittenberg, Halle (Saale), Germany; 9 Department of Plastic, Reconstructive, Aesthetic and Hand Surgery Basel, University Hospital of Basel, Basel, Switzerland; 10 Department of Surgery, University Hospital of Geneva, University of Geneva, Geneva, Switzerland; 11 Philipps-Universität Marburg, School of Medicine, Clinic for Pediatrics, Marburg, Germany; 12 Commission Digital Medicine, German Society of Gynecology and Obstetrics, Berlin, Germany

**Keywords:** Clinical decision support, Large language models, Artificial intelligence, Breast cancer, Benchmarking study

## Abstract

Medically specialized AI systems that have obtained regulatory clearance as medical devices can be deployed for patient-specific clinical decision support under defined compliance requirements. However, it remains unclear whether medical specialization and regulatory status translate into higher-quality breast cancer treatment recommendations than those produced by a general-purpose large language model (LLM). This blinded, multicenter study compared the performance of two medically specialized AI systems with a general-purpose model in breast cancer care. Two medically specialized (Prof. Valmed and OpenEvidence) and one general-purpose system (ChatGPT-5 Thinking) were prompted to generate treatment plans for 20 standardized breast cancer patient cases. Outputs were rated and ranked by blinded, board-certified breast cancer specialists from seven university breast cancer centers for safety, guideline adherence, medical adequacy, completeness, overall quality, and logical coherence. Statistical analyses comprised descriptive statistics, inter-rater reliability assessment, non-parametric performance comparisons of rating and ranking outcomes, and correlation analyses. Mean (± standard deviation) processing time for ChatGPT-5 Thinking (159 ± 58 s) was more than fourfold higher than that of Prof. Valmed (35 ± 4) and OpenEvidence (9 ± 1). ChatGPT-5 Thinking achieved significantly higher ratings across all evaluation categories, with no significant differences between the two medically specialized systems. Treatment plans generated by ChatGPT-5 Thinking were ranked as the top choice in 96.4% of rater-case combinations, compared with 3.6% for OpenEvidence, while Prof. Valmed was never ranked first. In this blinded, multicenter evaluation, a general-purpose LLM outperformed two medically specialized, retrieval-augmented systems in generating breast cancer treatment plans across all assessed categories. These results indicate that while regulatory clearance and domain specialization address key requirements for AI-enabled clinical decision support systems, these factors alone do not translate into superior performance in breast cancer care. At present, medically specialized systems may be best used as supportive tools under expert oversight, while further optimization and real-world validation are needed.

Clinical trial number: Not applicable

**Supplementary Information:**

The online version contains supplementary material available at 10.1007/s10916-026-02434-w.

## Introduction

Breast cancer is the most diagnosed cancer worldwide and is projected to remain a major contributor to global cancer mortality, with breast cancer deaths estimated to increase by more than 50%, from 685,000 in 2020 to approximately 1 million in 2040 if current trends remain unchanged [1]. The expanding precision oncological approach in breast cancer care requires physicians to assess large amounts of multimodal treatment data and diverse potential therapeutic plans with increasing detail in order to identify optimal, individualized treatment regimens [2, 3]. This represents a complex, time-consuming challenge that may be prone to errors when clinical decision-making is not aligned with the current and fast-moving development of scientific evidence.

Artificial intelligence (AI), with its ability to consider and integrate vast amounts of data and capability in algorithm-based reasoning, is regarded as a lever to address this healthcare challenge [4–6]. In parallel, the number of FDA-cleared (U.S. Food and Drug Administration) AI-enabled medical devices has increased about fourfold over the past five years, reaching 1,357 certified devices at the end of 2025 [7]. Approximately three-quarters of these devices are registered in the medical domain of radiology, and predominantly rely on machine and deep learning-based algorithmic approaches [7]. The clinical value of AI-enabled medical devices in the field of breast cancer care has been showcased by Eisemann et al., who demonstrated the benefits of integrating AI into the German national breast cancer screening program in a real-world setting. Their study involved more than 460,000 mammographies, achieving a 17.6% higher cancer detection rate for AI-supported compared to standard expert screening [8].

With the increasing prevalence of generative AI models, their relevance in medical research and clinical applications has increased rapidly [9]. In oncology, large language models (LLM) are being investigated across multiple use cases, including clinical decision support, diagnostic performance, data extraction, and supportive management for patients [10]. Accordingly, medical domain-specific LLM-enabled systems are increasingly becoming integral to the continuous evolution of clinical decision support. Prof. Valmed (Validated Medical Information GmbH, Langen, Germany), a subscription-based, retrieval-augmented generation (RAG) clinical decision-support system, is the first LLM-based solution in the European Union to achieve certification as a class IIb medical device, thereby meeting stringent regulatory requirements for clinical approval [11]. Furthermore, OpenEvidence (OpenEvidence, Cambridge, MA, USA), another RAG-based LLM system, has obtained compliance with the U.S. Health Insurance Portability and Accountability Act (HIPAA) and completed a SOC 2 Type II audit (System and Organization Controls; American Institute of Certified Public Accountants, AICPA), underscoring the implementation of independently verified, privacy-preserving approaches to patient data handling.

While the performance of publicly available LLMs in breast cancer care decision support has been evaluated in multiple studies [12, 13], regulatory authorization as a medical device of such AI-enabled systems represents a fundamentally new context. Such approval permits direct use in clinical decision-making and the processing of patient-specific data. Importantly, Kremer et al. previously addressed this aspect by evaluating OpenEvidence, Prof. Valmed, and the publicly available general-purpose AI system ChatGPT-5 Thinking (OpenAI, San Francisco, CA, USA) with a focus on diagnostic performance for rheumatology cases [14]. However, their performance in breast cancer treatment planning has not yet been evaluated. Considering this gap, the Commission Digital Medicine of the German Society for Gynecology and Obstetrics has decided to conduct a benchmarking study with two AI-enabled, medically specialized systems, Prof. Valmed and OpenEvidence, and compare them with one of the most widely used general-purpose AI systems, ChatGPT-5 Thinking.

The aim of this study was to compare the decision-support performance of these two medically specialized, regulated systems with a general-purpose LLM in breast cancer care by applying a previously used multidimensional rating framework in a blinded, multicenter setting across university breast cancer centers in Germany. The findings aim to inform clinical decision-makers about the current performance and appropriate use of domain-specific AI-enabled decision support systems in breast cancer care.

## Methods

### Study design

**Fig. 1:**
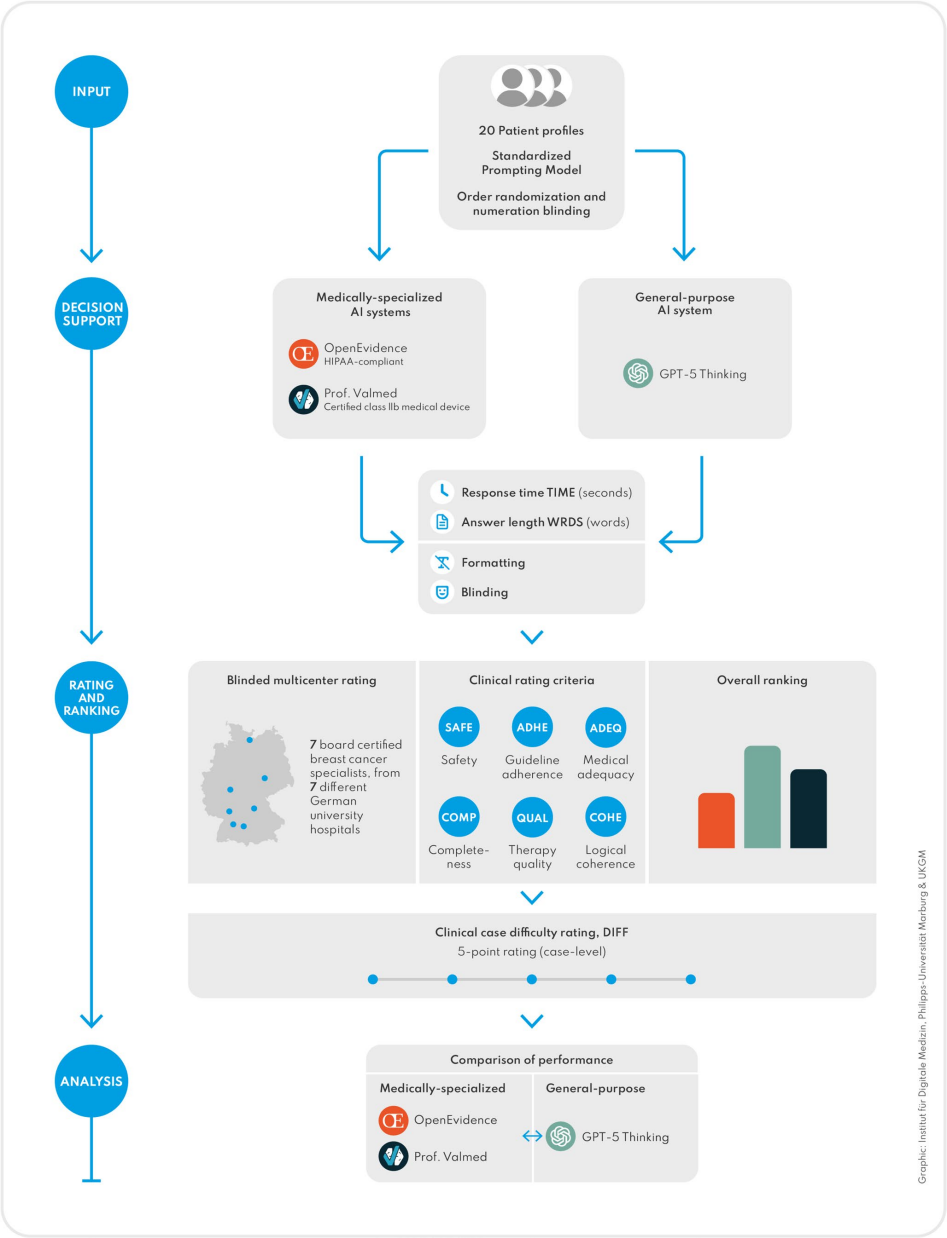
Overview of study design

We conducted a prospective, expert-blinded evaluation of the performance of three AI-enabled systems for clinical decision support in breast cancer care. The analysis included two medical domain-specific systems with different regulatory authorization status (Prof. Valmed and OpenEvidence) as well as one non-domain-specific general-purpose system (ChatGPT-5 Thinking). Each model was queried with a standardized, previously tested and breast cancer specific prompt to provide a singular treatment plan per patient profile in a single-turn interaction based on the most relevant and current international literature [13, 15–17]. Outputs were rated in a blinded manner by board-certified breast cancer specialists from different German university breast cancer centers according to a previously used, multidimensional rating framework for clinical decision support [18]. Subsequently, each rater ranked the three systems per case according to their personal preference. Figure 1 provides an overview of the study design.

### Input

Twenty breast cancer patient profiles, originally developed and published by Griewing et al. [13], were used to represent a range of complex clinical presentations and decision-making situations in breast cancer care, rather than to enable subtype-specific comparisons within individual disease stages. These included three patients with precancerous tumor stages, thirteen with invasive breast cancer (early stage), two with primary metastatic breast cancer, one with bilateral breast cancer, and one with inflammatory breast cancer. In addition, a differentiation by nodal status and postmenopausal status was made for each subtype. The patient profiles were reviewed, edited, and approved by the Head of the Gynecologic Oncology Center of Marburg University Hospital, Germany, to reflect clinical relevance and accuracy. Patient cases varied in complexity and required treatment recommendations in order to illustrate a spectrum ranging from straightforward medical scenarios to more challenging clinical situations with potential pitfalls.

Each patient profile included clinical information such as age, menopausal status, birth history, ECOG (Eastern Cooperative Oncology Group Performance Scale) [19], past medical history, oncological family history, and previous surgical treatment. Further pathological findings were provided to the extent of pTNM classification, minimal resection margin (R0/R1, in mm), histological classification (mucinous, tubular, invasive lobular, non-special-type (NST)), and grading (according to Bloom-Richardson-Elston score [20]), as well as unilaterality versus bilaterality and multifocality or multicentricity. Data regarding subtyping consisted of hormonal status (ER (estrogen receptor expression), 0-100%; PR (progesterone receptor expression), 0-100%), HER2-status (positive/negative; immunohistochemistry score (IHC), 0/1+/2+/3+), and the Ki-67 proliferation index (0-100%).

All case profiles were fictitious and did not represent real patients in order to adhere to data security and compliance requirements. Based on this, the Philipps-University Marburg Research Ethics Committee confirmed that no ethical approval was required (reference number: 23–300 ANZ) relating to the fictitious, non-interventional nature of the study.

### Decision support

All experiments were conducted from August 29th to September 14th, 2025 (in Marburg, Germany) to control for possible updates of the LLM systems. Before prompt execution, the order of profiles was randomized and subsequently applied identically across the three systems. All identifiers referencing profile numbers were removed.

ChatGPT-5 Thinking, Prof. Valmed, and OpenEvidence were queried for treatment recommendations using the 20 patient profiles. Each inquiry, initiated with the standardized prompt (see Online Resource 1), was conducted exactly once per system as an independent session with the chatbot’s memory function disabled. No follow-up questions were asked after the first input (single-turn interaction). For each query, response time (TIME, in seconds) and answer length (WRDS, word count) were recorded. To preserve blinding, all responses were compiled into one document, citations and URLs were removed, and minor formatting adjustments, including standardization of spacing, removal of Markdown syntax, and conversion of Markdown bold markers to standard bold formatting, were applied for consistency without altering content. The final format of the decision support answers is provided in Online Resource 2.

### Rating and ranking

Seven blinded, board-certified physicians, specialized in the field of breast cancer care, from seven German university hospital breast cancer centers (Erlangen, Halle (Saale), Heidelberg, Marburg, Luebeck, Tuebingen, and Ulm), individually rated the given decision support answers generated by each system for the patient profiles in the same sequential order within a single evaluation document. For each patient profile, the three anonymized system-generated answers were presented side by side in a fixed order. After submission of the completed evaluation document, no content-related changes were made. All therapy recommendations were assessed using a 5-point Likert scale (1 – strongly disagree, 2 – disagree, 3 – neither agree nor disagree, 4 – agree, 5 – strongly agree) following the rating framework proposed by Labinsky et al. [18], whereby the midpoint was intended to indicate a neutral or intermediate assessment of the respective domain rather than an absence of opinion. This included the categories of safety of the concept (SAFE), adherence to the current guidelines (ADHE), medical adequacy (ADEQ), completeness (COMP), therapy quality (QUAL), and logical coherence (COHE) of the reasoning behind the treatment decisions. Furthermore, the raters evaluated the clinical difficulty (DIFF) of each patient case on a 5-point Likert scale (1 – very easy, 2 – easy, 3 – moderate, 4 – difficult, 5 – very difficult) and ranked the generated answers for each patient profile in order of their personal preference (RANK, 1st /2nd /3rd rank).

### Analysis

Descriptive statistics were calculated for all variables. Inter-rater reliability (IRR) was assessed for each variable using Krippendorff’s *α* for ordinal data.

To compare system performance, scores were aggregated per case, and global differences between systems within each variable were analyzed using the non-parametric Friedman test for repeated measures, with effect sizes for the global tests reported as Kendall’s *W*. Where the Friedman test indicated significant differences (*p* < 0.05), pairwise Wilcoxon signed-rank tests with Holm-Bonferroni adjustments were applied. Additionally, a sensitivity analysis was conducted by repeating these comparisons for each rater individually to assess the consistency of the results.

In the analysis of the ranking data, the frequency of first-place rankings was tabulated for each model, and inter-rater agreement was evaluated by calculating Kendall’s *W* for each case. Differences in model rankings were tested using the Friedman test, followed by pairwise Wilcoxon signed-rank tests with Bonferroni correction. All statistical tests were two-sided, and significance was defined as *p* < 0.05.

In order to examine whether model performance correlated with perceived DIFF, Spearman rank correlations between the mean DIFF and both the mean Likert scores and the average RANK of each model across cases were calculated.

All analyses were conducted using Python (version 3.12.7) with the libraries pandas (version 2.3.2), NumPy (version 2.3.3), SciPy (version 1.16.3), statsmodels (version 0.14.5), krippendorff (version 0.8.2), and scikit-posthocs (version 0.11.4).

## Results

### Descriptive statistics

Mean TIME for ChatGPT-5 Thinking (arithmetic mean (AM) ± standard deviation (SD), 159 ± 58 s) was more than four times higher than those of Prof. Valmed (35 ± 4) and OpenEvidence (9 ± 1). Similarly, the WRDS for ChatGPT-5 Thinking (927 ± 247 words) was more than double that of Prof. Valmed (419 ± 63) and OpenEvidence (322 ± 55).

Mean case DIFF was rated as 2.54 ± 0.52 (AM ± SD), with most cases being rated as easy (43.6% of 140 rater-case combinations), followed by moderate (29.3%), difficult (16.4%), and very easy (10.0%). Only in one instance (0.7%) was a case rated as very difficult. Neither the mean Likert ratings nor the average RANK of the models showed a statistically significant correlation with DIFF (all Spearman *p* ≥ 0.1), suggesting that model performance and preferences were largely independent of perceived case difficulty.

### Results of rating

Overall, the seven raters showed moderate IRR on the Likert-scale ratings across the six rating categories, with Krippendorff’s *α* ranging from 0.460 (SAFE) to 0.596 (QUAL), while Friedman tests simultaneously revealed statistically significant differences between the three systems (*χ*²≈30–32, *p* < 0.001) with large effect sizes (Kendall’s *W* ≈ 0.75–0.81) (see Table 1).

**Table 1:** Inter-rater reliability and inferential statistics for category ratings and rankings

	Krippendorff’s α	Friedman χ²	*p value*	Kendall’s W	Pairwise comparisons
Rating					
SAFE	0.460	31.18	< 0.001	0.779	GPT > OE GPT > PV OE ≈ PV (n.s.)
ADHE	0.536	31.69	< 0.001	0.792	GPT > OE GPT > PV OE ≈ PV (n.s.)
ADEQ	0.550	32.43	< 0.001	0.811	GPT > OE GPT > PV OE ≈ PV (n.s.)
COMP	0.489	30.10	< 0.001	0.753	GPT > OE GPT > PV OE ≈ PV (n.s.)
QUAL	0.596	30.61	< 0.001	0.765	GPT > OE GPT > PV OE ≈ PV (n.s.)
COHE	0.536	31.69	< 0.001	0.792	GPT > OE GPT > PV OE ≈ PV (n.s.)
Ranking					
RANK	-	195.44	< 0.001	0.767	GPT ranked highest OE ≈ PV (n.s.)

Abbreviations: *SAFE* = safety of the concept, *ADHE* = adherence to current guidelines, *ADEQ* = medical adequacy of the concept, *COMP* = completeness of the concept, *QUAL* = therapy quality, *COHE* = logical coherence of the reasoning, *GPT* = ChatGPT-5 Thinking; OE = OpenEvidence, *PV* = Prof. Valmed; n.s. = not significantKrippendorff’s *α* = *IRR* for ordinal ratings, Kendall’s *W* = effect size for Friedman tests (for ratings) and concordance of rank order across raters (for rankings); Pairwise comparisons: Wilcoxon signed-rank tests with Holm-adjusted *p* values, > indicates significantly higher ratings

In post-hoc Wilcoxon signed-rank tests with Holm correction, ChatGPT-5 Thinking was rated significantly better than both OpenEvidence and Prof. Valmed in every category (all adjusted *p* < 0.001 and effect sizes *r* > 0.87), while OpenEvidence and Prof. Valmed did not differ significantly from each other (see Table 1). This pattern was replicated in sensitivity analyses conducted separately for each rater, where ChatGPT-5 Thinking consistently outperformed the other two systems.

Within each system, mean Likert scores were relatively consistent across the six rating categories. Further tests indicated that ChatGPT-5 Thinking showed a small overall category effect, with significant pairwise differences after correction, whereas OpenEvidence and Prof. Valmed exhibited moderate and statistically significant variation.

**Fig. 2:**
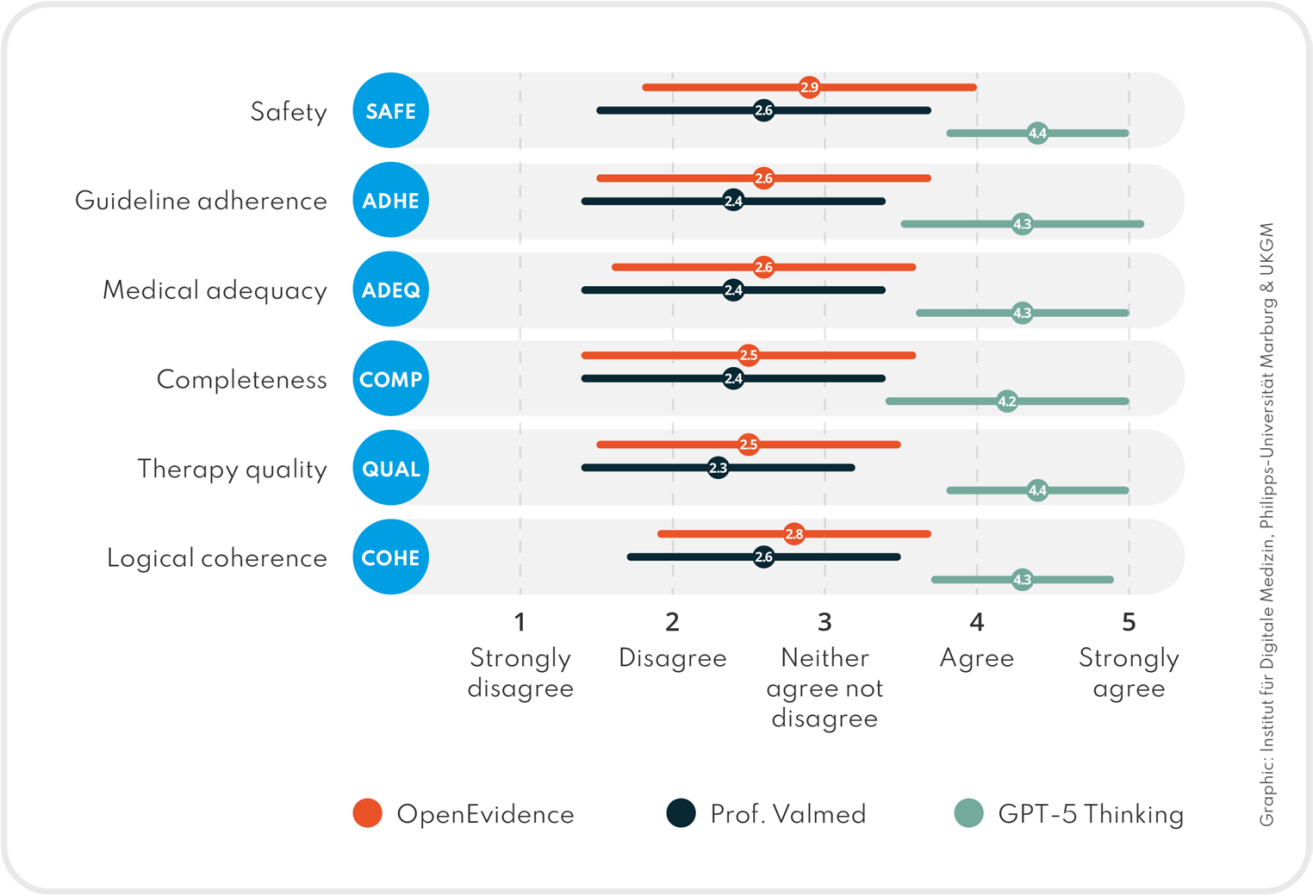
Mean expert category ratings (± standard deviation)

ChatGPT-5 Thinking achieved its highest mean scores in SAFE and QUAL (both 4.4 ± 0.6), followed by ADHE (4.3 ± 0.8), ADEQ (4.3 ± 0.7), and COHE (4.3 ± 0.6), while COMP (4.2 ± 0.8) showed the lowest category-specific mean. For Prof. Valmed, mean ratings were comparatively higher for SAFE (2.6 ± 1.1) and COHE (2.6 ± 0.9), whereas ADHE, ADEQ, and COMP (all 2.4 ± 1.0) were assessed less favorably, and QUAL (2.3 ± 0.9) received the least favorable rating. OpenEvidence showed a similar pattern, with relatively higher mean scores in SAFE (2.9 ± 1.1) and COHE (2.8 ± 0.9), followed by ADHE (2.6 ± 1.1) and ADEQ (2.6 ± 1.0), while COMP (2.5 ± 1.1) and QUAL (2.5 ± 1.0) received lower scores. Figure 2 summarizes the mean rating results based on the Labinsky et al. rating framework [18], showing higher scores for the general-purpose model ChatGPT-5 Thinking across all categories compared to the medically specialized systems.

### Results of ranking

**Fig. 3:**
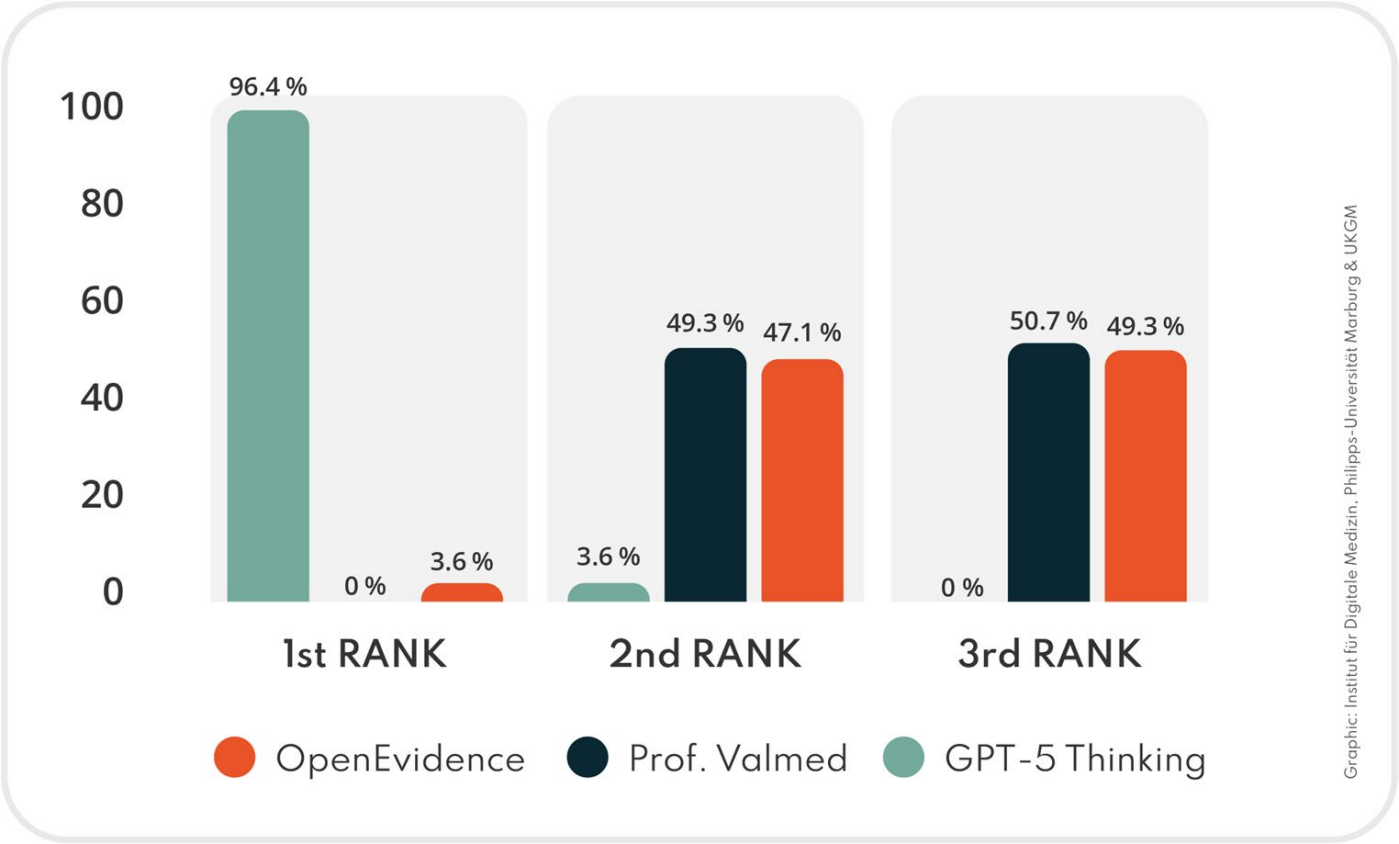
Rank distribution: proportion of 1st -, 2nd -, and 3rd - rank assignments

ChatGPT-5 Thinking was ranked first in the vast majority of evaluations, receiving the top rank in 135 out of 140 rater-case combinations (see Fig. 3). Inter-rater agreement on system rankings was substantial, with a high level of overall concordance across raters (mean Kendall’s *W* = 0.77, see Table 1). Minor systematic differences were detected and supported by *χ*²-tests (*p* = 0.033). A global Friedman test on the ranks showed a statistically significant model effect (*χ*²=195.44, *p* < 0.001). Pairwise Wilcoxon tests with Bonferroni correction confirmed that ChatGPT-5 Thinking was significantly preferred over both other models, whereas no significant difference in RANK was observed between Prof. Valmed and OpenEvidence (see Table 1). Figure 3 visualizes the distribution of 1st -, 2nd -, and 3rd -rank assignments and highlights the near-universal preference for the general-purpose model ChatGPT-5 Thinking, which was rarely ranked second and never ranked third.

## Discussion

### Main finding

To our knowledge, this is the first study to compare the performance of medically specialized and regulatory-cleared AI decision-support systems with a general-purpose AI system in the domain of breast cancer care. This comparison is clinically relevant as the introduction of OpenEvidence, with HIPAA compliance, and Prof. Valmed, a certified class IIb medical device according to EU standard, established a new regulatory and clinical context that enables direct use in clinical decision-making and the processing of patient-specific data. Such use has previously been a key limitation of publicly available, general-purpose LLMs, including ChatGPT-5 Thinking [21]. Overall, our study identified greater performance of the general-purpose system ChatGPT-5 Thinking in a blinded multicenter rating for breast cancer care compared to the domain-specific AI systems. Despite their potential for clinical decision support, the results do not currently justify the sole use of these systems; they require further domain-specific development and cautious application by informed experts in the context of clinical decision-making in breast cancer care. To better understand the clinical implications of this main finding, further results are discussed below, addressing specific performance dimensions in breast cancer care.

### Further findings

Across all rating criteria, ChatGPT-5 Thinking clearly outperformed both medically specialized systems, with mean Likert ratings ranging from 4.2 to 4.4, whereas OpenEvidence and Prof. Valmed mainly remained in the mid 2-point range. These differences were not only statistically significant but also associated with large effect sizes, and were reproduced in sensitivity analyses at the level of individual raters. Across evaluation criteria, ratings varied little for each model, with category-related differences being small for ChatGPT-5 Thinking and moderate for Prof. Valmed and OpenEvidence, suggesting no pronounced category-specific “blind spots”. In agreement with these results, clinicians preferred ChatGPT-5 Thinking to both OpenEvidence and Prof. Valmed in almost all cases, being ranked first in 96.4% of rater-case combinations. Similarly, in an early, not yet peer-reviewed study (December 2025), Vishwanath et al. benchmarked non-specialized LLMs, including ChatGPT-5, against medically specialized models such as OpenEvidence on 1,000 questions assessing clinical knowledge and alignment with expert judgment, and found that ChatGPT outperformed OpenEvidence (96.2% compared with 89.6% in knowledge accuracy; 97.0% compared with 74.3% in expert alignment) [22].

Notably, we further found that perceived DIFF did not correlate with either Likert ratings or RANK, indicating that model performance and preferences were largely independent of clinical difficulty. In line with this, Inojosa et al. compared ChatGPT-4o (OpenAI, San Francisco, CA, USA), a multiple sclerosis (MS) specific RAG system, and Prof. Valmed to postgraduate MS students (predominantly neurologists and neurology trainees) using multiple-choice and open-ended questions and found broadly similar overall accuracy, with a tendency toward better results for the two specialized systems, but comparable system performance across difficulty levels [23]. In their analysis, postgraduate students outperformed the models on easier questions but performed worse on the more difficult ones, suggesting that the added value of AI may be greatest in challenging cases.

Taken together, these convergent findings raise the question of why domain-specific, RAG-enabled systems currently appear to lag behind a general-purpose system in this focused setting. Several factors may contribute to this observation but given that the internal architectures of these systems are not publicly disclosed, it remains difficult to determine which mechanisms are responsible for the observed performance gap.

Under the European Medical Device Regulation, devices must comply with structured requirements concerning safety, performance, clinical evaluation, risk management, technical documentation, and post-market surveillance [24]. While these requirements are essential for market access and patient safety, they do not necessarily overlap with the multidimensional expert assessment applied in the present study. Moreover, the specific evidence base, evaluation scenarios, and performance thresholds underlying the authorization of individual AI-enabled clinical decision-support systems may not be fully transparent to external users. Therefore, regulatory status alone should not be regarded as a direct substitute for independent comparative evaluation, nor should it be assumed to imply superior performance in complex breast cancer treatment decision-support.

A further contributing factor may be that ChatGPT-5 Thinking, as observed in our study, consistently traded speed for more extensive and better-structured responses, whereas both medically specialized, RAG-based systems seemed to prioritize rapid output. This was reflected in a more than fourfold longer TIME for the general-purpose model, approaching 160 s. Thus, higher perceived quality was systematically accompanied by increased latency, which may pose a challenge for time-critical clinical settings, such as tumor boards with many consecutive cases, while being less problematic for preparatory work.

In addition, general-purpose models like ChatGPT are typically trained on extremely large and diverse corpora and are designed for broad language understanding and reasoning, which may translate into more nuanced therapeutic arguments and stronger knowledge retrieval [6, 25].

Our results contrast with a benchmarking study evaluating the same LLMs in a diagnostic rheumatology setting. Kremer et al. reported broadly similar diagnostic accuracy and processing times across medically specialized and general-purpose systems [14]. This discrepancy suggests that generating coherent, guideline-adherent treatment strategies imposes greater demands on reasoning depth, longitudinal coherence, and contextual integration than diagnostic ranking alone. Accordingly, evaluations of clinical LLMs should explicitly differentiate between diagnostic and therapeutic use cases, rather than assuming performance generalizes uniformly across clinical tasks.

In clinical routine, the ability to provide transparent, verifiable recommendations supported by underlying evidence is highly relevant for clinician trust and integration into evidence-based workflows [26, 27]. Domain-specific medical systems offer clear advantages in this regard, most notably through structured and verifiable citations to guidelines and peer-reviewed literature from validated sources - features that were not captured in our blinded evaluation. Consistent with prior work showing that general-purpose systems may frequently fabricate or misattribute references [28, 29], Hooshiar [30] reported reference hallucination in 82.2% of citations generated by ChatGPT-4o, compared with 0.0% for OpenEvidence, in a study on implant dentistry. Because all citations and URLs were removed to maintain blinding, raters could not benefit from the traceability and auditability inherent to RAG-based systems. Consequently, the practical clinical value of these models may be underestimated by text-only evaluations, even if their generated narratives appear less refined than those of general-purpose systems.

Furthermore, both specialized models offer strong data protection and privacy safeguards, an aspect that is not captured in blinded, text-only evaluations.

Our data cannot disentangle these factors, but they suggest that regulatory clearance and domain specialization alone do not guarantee superior clinical output quality. However, while ChatGPT-5 Thinking generated treatment plans consistently judged as safer, more guideline concordant, and more logically reasoned, that does not imply the absence of clinically important errors or sufficiency for independent use. Given the high-stakes nature of oncologic decision-making, any AI system output must therefore remain strictly advisory and subject to human verification.

### Limitations

Several limitations should be acknowledged. First, the ratings were based on multicenter expert consensus rather than a predefined case-specific gold standard. While this approach reflects real-world clinical reasoning, it introduces subjectivity, as assessments may be influenced by individual and institutional preferences.

Second, substantial differences in response length and structure were observed between the systems. Although raters were instructed to focus on content quality, differences in answer length may have introduced a systematic bias favoring more extensive responses. In addition, the presentation of the generated answers in a fixed order might have introduced a positional or expectation bias favoring the response presented in a recurring position.

Third, to ensure rater blinding, we removed citations, URLs, and other source attributions prior to evaluation. While this step was necessary to reduce brand recognition and confirmation bias, it may have systemically disadvantaged RAG-systems.

Fourth, model behavior is strongly influenced by prompt design and platform-specific AI guardrails. Although we used a standardized prompt across systems, differences in formatting preferences, instruction following and default verbosity, could have influenced expert rating beyond underlying medical knowledge. As the success of blinding was not formally assessed, incomplete blinding cannot be fully excluded.

Fifth, due to the rapidly evolving nature of LLM-based systems, model updates or changes in system behavior may lead to variability in outputs over time, even when identical prompts are used. Accordingly, our findings represent a time-specific assessment and may not be fully generalizable to later model versions.

Sixth, the study relied on single-turn interactions without follow-up questions, which may not adequately capture typical clinical use, where iterative clarification and refinement are common.

Finally, although expert raters were recruited from multiple breast cancer centers, all were embedded within the German healthcare system. This limits generalizability of the findings to other healthcare environments with different clinical guidelines, regulatory frameworks, and resource constraints. Furthermore, the limited number and distribution of patient profiles across clinical stages and biological subtypes restrict the extent to which subtype-specific conclusions can be drawn.

### Future research directions

The results point to several important directions for future research. Subsequent studies should extend the case-based design to larger and more diverse real-world patient cohorts. Furthermore, future study designs should include an international multicenter rating approach. In addition, future evaluations should incorporate multi-turn interactions in real-time, ideally within the context of multidisciplinary tumor boards. Such designs would allow for systematic analysis of acceptance, modification, or rejection of model-generated recommendations and provide deeper insight into the clinical integration, safety, and utility of AI-enabled decision support systems in breast cancer care.

## Conclusion

In this blinded, multicenter benchmarking study, a general-purpose LLM (ChatGPT-5 Thinking) consistently outperformed two medically specialized LLM-enabled systems (OpenEvidence and Prof. Valmed) in breast cancer treatment decision support across multiple clinically relevant categories. These findings suggest that domain specialization and regulatory authorization as a medical device alone do not currently translate into superior perceived decision-support quality. While regulated systems offer important advantages in terms of data protection, traceability, and regulatory compliance, their current performance does not justify independent clinical use. Therefore, expert oversight is currently required for all evaluated systems, regardless of regulatory authorization or domain specialization, and prospective real-world validation before routine clinical implementation, as well as post-market monitoring and periodic clinical reassessment thereafter, remain crucial to ensure sustained alignment with evolving clinical evidence and guidelines as well as rapid technological progress.

## Supplementary Information

Below is the link to the electronic supplementary material.

Supplementary Material 1 (Breast cancer specific prompt)

Supplementary Material 2 (Final format of the decision support answers)

## Data Availability

Data are provided within the manuscript or supplementary material. Further datasets generated and analyzed during the current study are available from the corresponding author on reasonable request.

## Declarations

### Explanation of AI-assisted technologies in the writing process

During the proofreading of this paper, the authors used ChatGPT-5.2 to improve readability; after using this tool, the authors reviewed and revised the content as needed and take full responsibility for the content of the publication.

## Abbreviations

ADHE: Guideline adherence
ADEQ: Medical adequacy
AI: Artificial intelligence
AICPA: American Institute of Certified Public Accountants
AM: Arithmetic mean
COHE: Logical coherence of the reasoning
COMP: Therapy completeness
DIFF: Clinical difficulty
ECOG: Eastern Cooperative Oncology Group Performance Scale
ER: Estrogen-receptor
FDA: U.S. Food and Drug Administration
HIPAA: U.S. Health Insurance Portability and Accountability Act
IHC: Immunehistochemistry
IRR: Inter-rater reliability
LLM: Large language model
MS: Multiple sclerosis
NST: Non-special-type
PR: Progesterone-receptor
QUAL: Therapy quality
RAG: Retrieval-augmented generation
SAFE: Therapy safety
SD: Standard deviation
SOC: System and Organization Controls
TIME: Response time (in seconds)
WRDS: Answer length (word count)
