# Supplementary material for: AI-enabled clinical decision support in breast cancer care: a blinded multicenter benchmarking study comparing medically specialized with a general-purpose system
Source: J Med Syst. 2026 Jul 4;50(1):107. doi: 10.1007/s10916-026-02434-w (PMC13332881; doi:10.1007/s10916-026-02434-w)
Supplement: Supplementary file 1 — Supplementary Material 1 (Breast cancer specific prompt) [file 10916_2026_2434_MOESM1_ESM.docx]

**Online Resource 1:**

**Breast cancer specific prompt**

AI-enabled clinical decision support in breast cancer care:

a blinded multicenter benchmarking study comparing medically specialized, regulated systems with a general-purpose large language model

Journal of Medical Systems, The Home of Clinical Informatics Research, Springer

Jonas Freudenberg (1)

Johannes Knitza (1)

Niklas Gremke (2)

Niklas Amann (3)

Thomas M. Deutsch (4,12)

Nikolas Tauber (5,12)

Kerstin Muras (5)

Zoe S. Oftring (1, 11)

Tobias Engler (6,12)

Alexander Englisch (6,12)

Stefan Lukac (7,12)

Adriano Fabi (9)

André S. Alves (10)

Kristin Reinhardt (8)

Markus Wallwiener (8,12)

Moritz Kuhlmann (1)

Jonathan Bamberger (1)

Uwe Wagner (2,12)

Sebastian Kuhn (1)

Sebastian Griewing* (1,2,12)

1) Philipps-Universität Marburg, Department of Medicine, Institute for Digital Medicine, Marburg, Germany

2) Philipps-Universität Marburg, School of Medicine, Clinic for Gynecology and Obstetrics, Marburg, Germany

3) Erlangen University Breast Center, University Hospital Erlangen, Friedrich Alexander University of Erlangen-Nuremberg, Erlangen, Germany

4) Heidelberg University Breast Center, University Hospital Heidelberg, University of Heidelberg, Heidelberg, Germany

5) Luebeck University Breast Center, University Hospital Schleswig-Holstein, Campus Luebeck, University of Luebeck, Luebeck, Germany

6) Tuebingen University Breast Center, University Hospital Tuebingen, Eberhard Karls University of Tuebingen, Tuebingen, Germany

7) Department of Obstetrics and Gynecology, University Hospital Ulm, University of Ulm, Ulm, Germany

8) Halle-Wittenberg University Breast Center, University Hospital Halle (Saale), Martin Luther University of Halle-Wittenberg, Halle (Saale), Germany

9) Department of Plastic, Reconstructive, Aesthetic and Hand Surgery Basel, University Hospital of Basel, Basel, Switzerland

10) Department of Surgery, University Hospital of Geneva, University of Geneva, Geneva, Switzerland

11) Philipps-Universität Marburg, School of Medicine, Clinic for Pediatrics, Marburg, Germany

12) Commission Digital Medicine, German Society of Gynecology and Obstetrics, Berlin, Germany

*Corresponding author: Sebastian Griewing (s.griewing@uni-marburg.de)

How should the following breast cancer patient be treated based on the most relevant and current international literature?

***[Insert patient information here]***

Following the surgical treatment, the following TNM-classification, resection margin, histological classification, grading and immunohistology were identified:

***[Insert histopathology report here]***

Please provide a step-by-step treatment recommendation with regard to further surgical treatment, endocrine treatment, systemic treatment and radiation therapy taking the given patient information in consideration.

If endocrine treatment is advisable, please provide a suitable treatment regimen.

If systemic treatment is advisable, please provide a suitable treatment regimen.

If radiation therapy is advisable, please provide a suitable treatment regimen.

Based on the oncological family history, please state whether genetic testing should be performed or not.
