# Supplementary material for: AI-enabled clinical decision support in breast cancer care: a blinded multicenter benchmarking study comparing medically specialized with a general-purpose system
Source: J Med Syst. 2026 Jul 4;50(1):107. doi: 10.1007/s10916-026-02434-w (PMC13332881; doi:10.1007/s10916-026-02434-w)
Supplement: Supplementary file 2 — Supplementary Material 2 (Final format of the decision support answers) [file 10916_2026_2434_MOESM2_ESM.docx]

**Online Resource 2:**

**Final format of the decision support answers**

AI-enabled clinical decision support in breast cancer care:

a blinded multicenter benchmarking study comparing medically specialized, regulated systems with a general-purpose large language model

Journal of Medical Systems, The Home of Clinical Informatics Research, Springer

Jonas Freudenberg (1)

Johannes Knitza (1)

Niklas Gremke (2)

Niklas Amann (3)

Thomas M. Deutsch (4,12)

Nikolas Tauber (5,12)

Kerstin Muras (5)

Zoe S. Oftring (1, 11)

Tobias Engler (6,12)

Alexander Englisch (6,12)

Stefan Lukac (7,12)

Adriano Fabi (9)

André S. Alves (10)

Kristin Reinhardt (8)

Markus Wallwiener (8,12)

Moritz Kuhlmann (1)

Jonathan Bamberger (1)

Uwe Wagner (2,12)

Sebastian Kuhn (1)

Sebastian Griewing* (1,2,12)

1) Philipps-Universität Marburg, Department of Medicine, Institute for Digital Medicine, Marburg, Germany

2) Philipps-Universität Marburg, School of Medicine, Clinic for Gynecology and Obstetrics, Marburg, Germany

3) Erlangen University Breast Center, University Hospital Erlangen, Friedrich Alexander University of Erlangen-Nuremberg, Erlangen, Germany

4) Heidelberg University Breast Center, University Hospital Heidelberg, University of Heidelberg, Heidelberg, Germany

5) Luebeck University Breast Center, University Hospital Schleswig-Holstein, Campus Luebeck, University of Luebeck, Luebeck, Germany

6) Tuebingen University Breast Center, University Hospital Tuebingen, Eberhard Karls University of Tuebingen, Tuebingen, Germany

7) Department of Obstetrics and Gynecology, University Hospital Ulm, University of Ulm, Ulm, Germany

8) Halle-Wittenberg University Breast Center, University Hospital Halle (Saale), Martin Luther University of Halle-Wittenberg, Halle (Saale), Germany

9) Department of Plastic, Reconstructive, Aesthetic and Hand Surgery Basel, University Hospital of Basel, Basel, Switzerland

10) Department of Surgery, University Hospital of Geneva, University of Geneva, Geneva, Switzerland

11) Philipps-Universität Marburg, School of Medicine, Clinic for Pediatrics, Marburg, Germany

12) Commission Digital Medicine, German Society of Gynecology and Obstetrics, Berlin, Germany

*Corresponding author: Sebastian Griewing (s.griewing@uni-marburg.de)

**Patient 1**

How should the following breast cancer patient be treated based on the most relevant and current international literature?

Patient information:

Age: 62

Menopause status: postmenopausal

ECOG: 0

Previous illness: Bronchial asthma (no long-term therapy, acute therapy with inhaled corticosteroids and formoterol), arterial hypertension (with antihypertensive triple combination of diuretic, calcium antagonist and AT II antagonist)

Previous surgical treatment: Transverse laparotomy for hysterectomy because of hypermenorrhea and uterine myomatosus at age of 42, laparoscopic cholecystectomy at the age of 45, open appendectomy at the age of 29

Birth history: 1 vaginal birth at age of 32, 1 cesarean at the age of 34, 1 early abortion at the age of 30

Oncological family history: Maternal aunt with colon cancer at the age of 62

The patient underwent the following surgical treatment: Breast-conserving tumorectomy with sentinellymphnodectomy on the right side

Following the surgical treatment, the following TNM-classification, resection margin, histological classification, grading and immunohistology were identified:

TNM: pT1bN0MX

Resection margin: R0, minimal resection margin of 5 mm

Histological classification: NST

Grading: G1

Multifocality/-centricity: Monofocal and -centric

Estrogen receptor (ER): 95%

Progesterone receptor (PR): 80%

Her2-status: Negative (IHC 0)

Ki-67-proliferation-index: 10%

Please provide a step-by-step treatment recommendation with regard to further surgical treatment, endocrine treatment, systemic treatment and radiation therapy taking the given patient information in consideration.

If endocrine treatment is advisable, please provide a suitable treatment regimen.

If systemic treatment is advisable, please provide a suitable treatment regimen.

If radiation therapy is advisable, please provide a suitable treatment regimen.

Based on the oncological family history, please state whether genetic testing should be performed or not.

| **Patient 1 Treatment Concepts** |  |  |
| --- | --- | --- |
| **Concept 1** | **Concept 2** | **Concept 3** |
| **Treatment Recommendations for the Patient**   1. **Further Surgical Treatment**   - No further surgical treatment is required. The patient underwent breast-conserving surgery (tumorectomy) with sentinel lymph node biopsy, achieving clear resection margins (R0) with a minimal margin of 5 mm. This is sufficient for a pT1bN0M0, monofocal, low-grade (G1) tumor.   1. **Endocrine Treatment**   - Endocrine therapy is strongly recommended due to the high expression of estrogen (ER: 95%) and progesterone receptors (PR: 80%), indicating hormone receptor-positive breast cancer.  - Recommended regimen: Aromatase inhibitor (AI) therapy is preferred for postmenopausal women. A suitable regimen includes:  - Letrozole 2.5 mg orally once daily, or  - Anastrozole 1 mg orally once daily, or  - Exemestane 25 mg orally once daily.  - Duration: 5 years, with consideration for extending to 7–10 years based on tolerability and risk of recurrence.   1. **Systemic Treatment**   - Systemic chemotherapy is not indicated in this case. The tumor is low-risk based on the following factors:  - Small tumor size (pT1b, ≤1 cm).  - Low grade (G1).  - Low Ki-67 proliferation index (10%).  - Absence of lymph node involvement (N0).  - HER2-negative status.  - The patient’s excellent ECOG performance status (0) and comorbidities (controlled hypertension and asthma) do not necessitate systemic chemotherapy.   1. **Radiation Therapy**   - Radiation therapy is recommended following breast-conserving surgery to reduce the risk of local recurrence.  - Recommended regimen:  - Whole-breast irradiation (WBI) with a total dose of 40–50 Gy delivered in 15–20 fractions over 3–4 weeks.  - A boost dose of 10–16 Gy to the tumor bed may be considered, especially in younger patients or those with close margins, but this is less critical in this case due to the patient’s age and clear margins.   1. **Genetic Testing**   - Genetic testing is not routinely indicated in this patient. The family history includes a maternal aunt with colon cancer at age 62, which does not strongly suggest a hereditary breast cancer syndrome (e.g., BRCA1/2 mutation). Testing may be reconsidered if additional family history of breast, ovarian, or other BRCA-associated cancers emerges.  **Summary of Recommendations**  - Surgery: No further surgical intervention required.  - Endocrine therapy: Aromatase inhibitor for 5 years.  - Systemic therapy: No chemotherapy indicated.  - Radiation therapy: Whole-breast irradiation with a possible boost to the tumor bed.  - Genetic testing: Not indicated based on current family history. | Here’s a concise, guideline-based plan for this patient (62, post-menopausal; pT1b pN0, R0 5 mm, NST, G1, ER 95%, PR 80%, HER2-0, Ki-67 10%). This profile is classic low-risk luminal-A–like early breast cancer.  **1) Further surgery**   - No additional breast or axillary surgery. Margins are negative and adequate; for invasive cancer, “no ink on tumor” is sufficient—5 mm is more than adequate. Sentinel node is negative.   **2) Systemic therapy**  Endocrine therapy (indicated)   - Preferred: an aromatase inhibitor (AI) for 5 years   - *Examples*: Letrozole 2.5 mg PO daily, or Anastrozole 1 mg PO daily, or Exemestane 25 mg PO daily.   - Rationale: Post-menopausal, strongly ER+/PR+, HER2-. Five years is the standard; longer therapy (7–10 years) lowers recurrence modestly but is generally reserved for higher-risk features—here, 5 years is usually sufficient, with reassessment at year 5. - Bone health while on an AI: Baseline and q1–2 y DXA; calcium/vitamin D; weight-bearing exercise; manage arthralgias proactively.   Optional: adjuvant bisphosphonate   - Discuss adding zoledronic acid 4 mg IV every 6 months for 3 years (or oral clodronate/ibandronate) to reduce bone recurrence and slightly improve survival in post-menopausal women. Given this patient’s very low recurrence risk, benefit is small; decide by shared decision-making after dental check and renal review. Denosumab is not recommended for this purpose.   Chemotherapy / genomic testing   - No cytotoxic chemotherapy. Tumor is ≤1 cm (T1b), G1, ER-rich, HER2-, low Ki-67—chemo benefit is negligible. - Multigene assay (e.g., Oncotype DX) is not necessary here; assays are most useful when clinicopathologic risk is intermediate, which is not the case. (Retrospective series show nearly all T1b, grade 1, HR+/HER2- tumors score low.)   **3) Radiation therapy**  After breast-conserving surgery, adjuvant radiation is standard for women under ~65; omission is generally reserved for ≥65 with favorable biology who will take endocrine therapy (even then, omission raises in-breast recurrence). At age 62, recommend radiation.  You have two excellent, evidence-based options:  A) Whole-breast irradiation (WBI) – hypofractionated   - 26 Gy in 5 daily fractions over 1 week (FAST-Forward), no boost given age >50, G1, clear margins, and low risk; or - 40 Gy in 15 fractions over 3 weeks (also acceptable). Both regimens provide excellent control with low toxicity; the 1-week schedule is now widely endorsed.   B) Partial-breast irradiation (PBI) – recommended for this “suitable” low-risk profile (≥40–50 y, ≤2 cm, node-negative, ER+, clear margins)   - 30 Gy in 5 once-daily fractions on non-consecutive days over ~1–2 weeks (Florence/ASTRO-endorsed), delivered with 3D-CRT or IMRT. - PBI offers similar in-breast control with smaller treated volume and even greater convenience in appropriately selected patients like this one.   Which to pick? For most, PBI or 1-week WBI are both excellent; choose based on local expertise, anatomy, and patient preference. A tumor-bed boost is not indicated here.  **4) Staging / imaging**   - With asymptomatic stage I (T1bN0) disease, no routine systemic staging scans (CT/PET/bone scan) are recommended.   **5) Genetic testing**   - Based on family history alone (one maternal aunt with colon cancer at 62): germline testing is not indicated by traditional criteria. - However, current ASCO/SSO 2024 guidance recommends offering BRCA1/2 testing to all newly diagnosed patients ≤65 regardless of family history; testing can be extended to selected other high-penetrance genes when results would change management or inform family risk (e.g., PALB2). This patient (62) should be offered testing under these contemporary recommendations.   **Practical prescriptions (put on the chart)**   - Endocrine therapy: *Letrozole 2.5 mg PO daily x 5 years* (alternatives: anastrozole 1 mg daily, exemestane 25 mg daily). Start within ~6 weeks of surgery when feasible. Arrange baseline DXA, lipids, vitamin D; counsel on joint symptoms. - Radiation: either   - PBI 30 Gy/5 fx (non-consecutive days, ~1–2 weeks) *or*   - WBI 26 Gy/5 fx (daily x 1 week), no boost. - Consider adjuvant zoledronic acid 4 mg IV q6 months x 3 years after dental clearance, if patient values the small added protection against bone recurrence. - No chemotherapy. No further surgery. | This patient has early-stage, hormone receptor-positive, HER2-negative, grade 1, node-negative breast cancer (pT1bN0, ER 95%, PR 80%, HER2 0, Ki-67 10%) following breast-conserving surgery with clear margins. She is postmenopausal, with ECOG 0 and relevant comorbidities.  **Further surgical treatment:**  No additional breast or axillary surgery is indicated, as margins are negative (R0) and sentinel nodes are negative.  **Endocrine therapy:**  Adjuvant endocrine therapy is strongly recommended. For postmenopausal women, an aromatase inhibitor (anastrozole, letrozole, or exemestane) for at least 5 years is preferred, given superior efficacy over tamoxifen in this population. Tamoxifen is an alternative if aromatase inhibitors are contraindicated. The American Society of Clinical Oncology recommends 5 years of adjuvant endocrine therapy for node-negative, low-risk, ER-positive, HER2-negative breast cancer.  **Systemic chemotherapy:**  Chemotherapy is not indicated for this patient, as she has low-risk features: small tumor size, grade 1, low Ki-67, and node-negative status. The American Society of Clinical Oncology guideline states that chemotherapy is reserved for higher-risk tumors (larger size, higher grade, higher proliferation, or adverse genomic features).  **Radiation therapy:**  Radiation therapy may be omitted in select postmenopausal women with luminal A breast cancer (Ki-67 ≤13.25%, ER/PR positive, HER2 negative) who are receiving endocrine therapy, as shown in recent randomized trials. Shared decision-making is essential, but omission is supported by the latest evidence for this risk profile.  **Genetic testing:**  Genetic testing is not routinely indicated based on this family history (single maternal aunt with colon cancer at age 62, no breast/ovarian cancer or early-onset cancers). Testing may be considered if additional family history emerges.  **In summary:**  No further surgery, aromatase inhibitor for 5 years, no chemotherapy, radiation may be omitted, and no genetic testing is indicated. |

| **PATIENT CASE 1** |  | |  | |  |  |  |  |
| --- | --- | --- | --- | --- | --- | --- | --- | --- |
|  |  | |  | |  |  |  |  |
| **How difficult or easy was the patient case?** | **very easy** | | **easy** | | **moderate** | **difficult** | **very difficult** | **Score** |
| **Case 1** | **1** | | **2** | | **3** | **4** | **5** |  |
|  |  | |  | |  |  |  |  |
| **The therapy concept is safe.** | **strongly disagree** | | **disagree** | | **neither agree nor disagree** | **agree** | **strongly agree** | **Score** |
| **Therapy Concept 1** | **1** | | **2** | | **3** | **4** | **5** |  |
| **Therapy Concept 2** | **1** | | **2** | | **3** | **4** | **5** |  |
| **Therapy Concept 3** | **1** | | **2** | | **3** | **4** | **5** |  |
|  |  | |  | |  |  |  |  |
| **The therapy concept is in line with the current guidelines.** | **strongly disagree** | | **disagree** | | **neither agree nor disagree** | **agree** | **strongly agree** | **Score** |
| **Therapy Concept 1** | **1** | | **2** | | **3** | **4** | **5** |  |
| **Therapy Concept 2** | **1** | | **2** | | **3** | **4** | **5** |  |
| **Therapy Concept 3** | **1** | | **2** | | **3** | **4** | **5** |  |
|  |  | |  | |  |  |  |  |
| **The therapy concept is medically adequate.** | **strongly disagree** | | **disagree** | | **neither agree nor disagree** | **agree** | **strongly agree** | **Score** |
| **Therapy Concept 1** | **1** | | **2** | | **3** | **4** | **5** |  |
| **Therapy Concept 2** | **1** | | **2** | | **3** | **4** | **5** |  |
| **Therapy Concept 3** | **1** | | **2** | | **3** | **4** | **5** |  |
|  |  | |  | |  |  |  |  |
| **The therapy concept is complete.** | **strongly disagree** | | **disagree** | | **neither agree nor disagree** | **agree** | **strongly agree** | **Score** |
| **Therapy Concept 1** | **1** | | **2** | | **3** | **4** | **5** |  |
| **Therapy Concept 2** | **1** | | **2** | | **3** | **4** | **5** |  |
| **Therapy Concept 3** | **1** | | **2** | | **3** | **4** | **5** |  |
|  |  | |  | |  |  |  |  |
| **The therapy concept is of high quality.** | **strongly disagree** | | **disagree** | | **neither agree nor disagree** | **agree** | **strongly agree** | **Score** |
| **Therapy Concept 1** | **1** | | **2** | | **3** | **4** | **5** |  |
| **Therapy Concept 2** | **1** | | **2** | | **3** | **4** | **5** |  |
| **Therapy Concept 3** | **1** | | **2** | | **3** | **4** | **5** |  |
|  |  | |  | |  |  |  |  |
| **The reasoning behind the treatment decision is logical.** | **strongly disagree** | | **disagree** | | **neither agree nor disagree** | **agree** | **strongly agree** | **Score** |
| **Therapy Concept 1** | **1** | | **2** | | **3** | **4** | **5** |  |
| **Therapy Concept 2** | **1** | | **2** | | **3** | **4** | **5** |  |
| **Therapy Concept 3** | **1** | | **2** | | **3** | **4** | **5** |  |
|  |  | |  | |  |  |  |  |
| **Please rank the therapy concept according to your preference.** | | **Ranking (1^st^, 2^nd^, 3^rd^)** | |  |  |  |  |  |
| **Therapy Concept 1** | |  | |  |  |  |  |  |
| **Therapy Concept 2** | |  | |  |  |  |  |  |
| **Therapy Concept 3** | |  | |  |  |  |  |  |

**Patient 2**

How should the following breast cancer patient be treated based on the most relevant and current international literature?

Patient information:

Age: 61

Menopause status: postmenopausal

ECOG: 0

Previous illness: Hypothyroidism (with L-thyroxine medication)

Previous surgical treatment: Open cholecystectomy at the age of 35, breast-conserving tumorectomy for right-sided fibroadenoma at the age of 32, uterine curettage after early abortion at age of 20

Birth history: 4 vaginal births at the age of 25, 27, 29 and 30, 1 early abortion at the age of 20

Oncological family history: Maternal female cousin with hodgkin lymphoma at the age of 30

The patient underwent the following surgical treatment: Breast-conserving tumorectomy with sentinellymphnodectomy on the left side

Following the surgical treatment, the following TNM-classification, resection margin, histological classification, grading and immunohistology were identified:

TNM: pT2(2)pN1aM0

Resection margin: R0, minimal resection margin of 6 mm

Histological classification: Invasive-lobular

Grading: G2

Multifocality/-centricity: Monocentric and multifocal, 2 foci

Estrogen receptor (ER): 85%

Progesterone receptor (PR): 80%

Her2-status: Negative (IHC 1+)

Ki-67-proliferation-index: 15%

Please provide a step-by-step treatment recommendation with regard to further surgical treatment, endocrine treatment, systemic treatment and radiation therapy taking the given patient information in consideration.

If endocrine treatment is advisable, please provide a suitable treatment regimen.

If systemic treatment is advisable, please provide a suitable treatment regimen.

If radiation therapy is advisable, please provide a suitable treatment regimen.

Based on the oncological family history, please state whether genetic testing should be performed or not.

| **Patient 2 Treatment Concepts** |  |  |
| --- | --- | --- |
| **Concept 1** | **Concept 2** | **Concept 3** |
| **Treatment Recommendations for the Patient**  **1. Further Surgical Treatment**  - No further surgical treatment is required as the patient has undergone breast-conserving surgery (BCS) with sentinel lymph node dissection, achieving clear resection margins (R0, 6 mm). The TNM classification (pT2N1aM0) and histological findings do not indicate the need for additional surgery.  **2. Endocrine Treatment**  - Given the postmenopausal status, high estrogen receptor (ER, 85%) and progesterone receptor (PR, 80%) positivity, and HER2-negative status, endocrine therapy is strongly recommended.  - Treatment regimen: Aromatase inhibitor (AI) therapy is the preferred option for postmenopausal women.  Drug: Letrozole 2.5 mg orally once daily.  - Duration: 5 years, with consideration for extending therapy to 7–10 years based on risk assessment and tolerability.  **3. Systemic Treatment**  - Systemic chemotherapy is not recommended in this case due to the following factors:  - Low Ki-67 proliferation index (15%), indicating a less aggressive tumor.  - Hormone receptor positivity and HER2-negative status, which suggest a better response to endocrine therapy.  - Absence of high-risk features such as triple-negative or HER2-positive disease.  **4. Radiation Therapy**  - Postoperative radiation therapy is indicated following breast-conserving surgery to reduce the risk of local recurrence.  - Radiation regimen:  - Whole-breast irradiation (WBI) with a total dose of 50 Gy delivered in 25 fractions (2 Gy per fraction) over 5 weeks.  - A tumor bed boost of 10–16 Gy in 5–8 fractions may be considered, especially given the multifocal nature of the tumor.   1. **Genetic Testing**   - Genetic testing is not routinely indicated in this patient based on the provided oncological family history. The maternal female cousin with Hodgkin lymphoma at age 30 does not suggest a hereditary breast cancer syndrome. However, if there are additional family members with breast, ovarian, or other related cancers, further evaluation may be warranted.  **Summary of Recommendations**  1. No further surgical treatment is required.  2. Endocrine therapy with letrozole for 5 years is recommended.  3. Systemic chemotherapy is not indicated.  4. Postoperative radiation therapy with WBI (50 Gy in 25 fractions) and a tumor bed boost (10–16 Gy) is advised.  5. Genetic testing is not necessary based on the current family history. | Here’s a concise, guideline-concordant adjuvant plan tailored to this 61-year-old, postmenopausal patient with pT2 (multifocal, 2 foci) pN1a M0, ER+/PR+, HER2-negative, invasive lobular carcinoma (G2), Ki-67 ≈15%, R0 (6 mm) after breast-conserving surgery (BCS) with sentinel node biopsy.  **1) Further surgery**   - No re-excision: For invasive cancer, “no tumor on ink” is sufficient—including for invasive lobular carcinoma—so a 6 mm clear margin needs no further surgery. - Axilla: If 1–2 macrometastatic sentinel nodes and BCS with planned whole-breast irradiation (WBI), omit completion ALND (Z0011 paradigm). If ≥3 positive nodes, or gross extranodal extension, discuss completion ALND vs axillary RT (AMAROS) in a multidisciplinary board.   **2) Systemic therapy**  2a) Genomic assay to decide chemotherapy   - Order a validated genomic assay (e.g., Oncotype DX). In postmenopausal women with 1–3 positive nodes, RS 0–25 shows no benefit from adding adjuvant chemotherapy to endocrine therapy (RxPONDER). If RS ≥26, offer chemotherapy.   2b) Endocrine therapy (indicated)   - Start now: An aromatase inhibitor (AI) for at least 5 years (e.g., letrozole 2.5 mg daily, or anastrozole 1 mg, or exemestane 25 mg). - Extension: Given node-positive disease, extend to 7–10 years total if tolerated (AI throughout or TAM→AI sequence). Monitor bone and CV risk; switch to tamoxifen if AI intolerance.   2c) If chemotherapy is indicated (only if high genomic risk)   - Reasonable adjuvant regimens:   - TC ×4–6: docetaxel 75 mg/m² + cyclophosphamide 600 mg/m² q3 weeks, or   - AC→T: doxorubicin 60 mg/m² + cyclophosphamide 600 mg/m² q2–3 weeks ×4 → paclitaxel (80 mg/m² weekly ×12 or q2 weeks ×4). Choice depends on comorbidities and preferences; anthracycline–taxane regimens have the broadest evidence in higher-risk disease.   2d) CDK4/6 inhibitors?   - Not indicated here: abemaciclib is reserved for “high-risk” HR+/HER2– (≥4 nodes, or 1–3 nodes with G3 or tumor ≥5 cm); this tumor is G2, T2, and Ki-67 15%. (FDA no longer requires Ki-67, but high-risk clinicopathologic criteria still apply.)   2e) Bone-modifying therapy (recommended)   - In postmenopausal patients receiving adjuvant systemic therapy, discuss adjuvant bisphosphonates (reduce bone recurrence and breast-cancer mortality):   - Zoledronic acid 4 mg IV q6 months for 3–5 years, or   - Clodronate 1600 mg PO daily for 2–3 years, or   - Ibandronate 50 mg PO daily for 3 years. Do dental check, supplement Ca/Vit-D, check renal function. (Denosumab is not recommended for anticancer benefit.)   Note on Ki-67: 15% supports a luminal-A–like picture but should not be used alone to decide on chemotherapy; rely on a genomic assay.  **3) Radiation therapy**   - Whole-breast irradiation (WBI) after BCS is indicated. Acceptable hypofractionation:   - 40 Gy in 15 fractions (3 weeks), or   - 26 Gy in 5 fractions (FAST-Forward, 1 week)—where available/appropriate. - Tumor-bed boost: At age 61 with node-positive, multifocal disease, a boost (e.g., 10–16 Gy in 4–8 fx) is reasonable to lower in-breast recurrence; weigh against fibrosis. - Regional nodal irradiation (RNI): With pN1 (1–3 nodes), strongly consider RNI (supraclavicular ± level III axilla; add internal mammary nodes if medial/central tumor or other risk factors). Trials show reduced recurrences and breast-cancer mortality (no OS difference). Use modern planning to minimize toxicity. - Left-sided cardiac sparing: Use deep-inspiration breath-hold (DIBH) to reduce heart dose.   **4) Practical sequencing**   1. Final pathology review confirms Z0011 eligibility (if only 1–2 positive SLNs): no further axillary surgery. 2. Order Oncotype DX (or similar) to decide on chemotherapy. 3. Start AI; if chemo is needed (RS ≥ 26), give chemo first, then start/endocrine. 4. Begin WBI ± RNI (with DIBH) after wound healing; give boost. 5. Start bisphosphonate therapy soon after systemic therapy starts.   **5) Germline testing**   - Yes—offer germline testing. ASCO/SSO now recommend offering BRCA1/2 testing to all patients ≤65 with current or prior breast cancer; multigene panels (often including PALB2, CHEK2, ATM, etc.) are commonly used. (CDH1 testing is typically reserved for lobular cancer with personal/family history of diffuse gastric cancer, which is not present here.)   - If a pathogenic BRCA1/2 variant is found and high-risk criteria are met, consider 1 year of adjuvant olaparib; with pT2N1 (1–3 nodes) treated upfront with surgery, most HR+ patients will not meet OlympiA’s high-risk thresholds, but this depends on full details.   One possible “default” regimen (if RS ≤ 25; chemo omitted)   - Endocrine: Letrozole 2.5 mg PO daily × 5 years, then discuss extension to 7–10 years based on tolerance/risk. - Bone health: Zoledronic acid 4 mg IV q6 mo × 3–5 y; DEXA at baseline and periodically. - Radiation: WBI 40 Gy/15 fx (or 26 Gy/5 fx) + boost; RNI to SCV ± IMN based on tumor location/risk; DIBH for left-sided sparing.   This plan reflects current international evidence and consensus and should be finalized in a multidisciplinary setting with the patient’s preferences and local resources in mind. | This postmenopausal woman with pT2(2)pN1aM0, ER+/PR+, HER2-negative, G2, multifocal invasive lobular carcinoma (2 foci, monocentric), Ki-67 15%, and R0 resection after breast-conserving surgery requires a multidisciplinary adjuvant approach.  **Further surgical treatment:**  No further surgery is indicated, as margins are negative (R0, 6 mm) and axillary staging is complete with sentinel node dissection  **Endocrine therapy:**  Adjuvant endocrine therapy is strongly indicated for at least 5 years, given high ER/PR expression. In postmenopausal women, an aromatase inhibitor (e.g., anastrozole 1 mg daily, letrozole 2.5 mg daily, or exemestane 25 mg daily) is preferred over tamoxifen due to superior efficacy in reducing recurrence. Extended therapy up to 10 years may be considered based on risk and tolerability. The American Society of Clinical Oncology (ASCO) and the St. Gallen International Expert Consensus recommend aromatase inhibitors as first-line adjuvant endocrine therapy in this setting.  **Systemic (chemotherapy) therapy:**  For postmenopausal women with 1-3 positive nodes, the ASCO guideline states that multigene assays (e.g., Oncotype DX) may be used to guide chemotherapy decisions. If the recurrence score is low (<26), chemotherapy can be omitted; if ≥26, adjuvant chemotherapy should be offered. In the absence of a genomic assay, clinical risk (node positivity, tumor size, multifocality) supports consideration of chemotherapy, but the modest Ki-67 and intermediate grade suggest benefit may be limited. If chemotherapy is chosen, standard regimens include anthracycline- and/or taxane-based protocols.  **Radiation therapy:**  Adjuvant whole-breast irradiation is indicated after breast-conserving surgery. Given nodal involvement and multifocality, regional nodal irradiation (including supraclavicular and axillary nodes) should be considered.  **Genetic testing:**  Routine germline genetic testing is not indicated based on this family history (only a maternal cousin with Hodgkin lymphoma, no breast/ovarian cancer or other high-risk features).  **In summary:**  Aromatase inhibitor for at least 5 years, adjuvant radiotherapy (whole breast and regional nodes), consider adjuvant chemotherapy guided by genomic risk assessment, and no further surgery or genetic testing required. |

| **PATIENT CASE 2** |  | |  | |  |  |  |  |
| --- | --- | --- | --- | --- | --- | --- | --- | --- |
|  |  | |  | |  |  |  |  |
| **How difficult or easy was the patient case?** | **very easy** | | **easy** | | **moderate** | **difficult** | **very difficult** | **Score** |
| **Case 2** | **1** | | **2** | | **3** | **4** | **5** |  |
|  |  | |  | |  |  |  |  |
| **The therapy concept is safe.** | **strongly disagree** | | **disagree** | | **neither agree nor disagree** | **agree** | **strongly agree** | **Score** |
| **Therapy Concept 1** | **1** | | **2** | | **3** | **4** | **5** |  |
| **Therapy Concept 2** | **1** | | **2** | | **3** | **4** | **5** |  |
| **Therapy Concept 3** | **1** | | **2** | | **3** | **4** | **5** |  |
|  |  | |  | |  |  |  |  |
| **The therapy concept is in line with the current guidelines.** | **strongly disagree** | | **disagree** | | **neither agree nor disagree** | **agree** | **strongly agree** | **Score** |
| **Therapy Concept 1** | **1** | | **2** | | **3** | **4** | **5** |  |
| **Therapy Concept 2** | **1** | | **2** | | **3** | **4** | **5** |  |
| **Therapy Concept 3** | **1** | | **2** | | **3** | **4** | **5** |  |
|  |  | |  | |  |  |  |  |
| **The therapy concept is medically adequate.** | **strongly disagree** | | **disagree** | | **neither agree nor disagree** | **agree** | **strongly agree** | **Score** |
| **Therapy Concept 1** | **1** | | **2** | | **3** | **4** | **5** |  |
| **Therapy Concept 2** | **1** | | **2** | | **3** | **4** | **5** |  |
| **Therapy Concept 3** | **1** | | **2** | | **3** | **4** | **5** |  |
|  |  | |  | |  |  |  |  |
| **The therapy concept is complete.** | **strongly disagree** | | **disagree** | | **neither agree nor disagree** | **agree** | **strongly agree** | **Score** |
| **Therapy Concept 1** | **1** | | **2** | | **3** | **4** | **5** |  |
| **Therapy Concept 2** | **1** | | **2** | | **3** | **4** | **5** |  |
| **Therapy Concept 3** | **1** | | **2** | | **3** | **4** | **5** |  |
|  |  | |  | |  |  |  |  |
| **The therapy concept is of high quality.** | **strongly disagree** | | **disagree** | | **neither agree nor disagree** | **agree** | **strongly agree** | **Score** |
| **Therapy Concept 1** | **1** | | **2** | | **3** | **4** | **5** |  |
| **Therapy Concept 2** | **1** | | **2** | | **3** | **4** | **5** |  |
| **Therapy Concept 3** | **1** | | **2** | | **3** | **4** | **5** |  |
|  |  | |  | |  |  |  |  |
| **The reasoning behind the treatment decision is logical.** | **strongly disagree** | | **disagree** | | **neither agree nor disagree** | **agree** | **strongly agree** | **Score** |
| **Therapy Concept 1** | **1** | | **2** | | **3** | **4** | **5** |  |
| **Therapy Concept 2** | **1** | | **2** | | **3** | **4** | **5** |  |
| **Therapy Concept 3** | **1** | | **2** | | **3** | **4** | **5** |  |
|  |  | |  | |  |  |  |  |
| **Please rank the therapy concept according to your preference.** | | **Ranking (1^st^, 2^nd^, 3^rd^)** | |  |  |  |  |  |
| **Therapy Concept 1** | |  | |  |  |  |  |  |
| **Therapy Concept 2** | |  | |  |  |  |  |  |
| **Therapy Concept 3** | |  | |  |  |  |  |  |

**Patient 3**

How should the following breast cancer patient be treated based on the most relevant and current international literature?

Patient information:

Age: 50

Menopause status: premenopausal

ECOG: 1

Previous illness: Relapsing remitting multiple sclerosis (last episode 5 years ago, no long-term medication)

Previous surgical treatment: Tonsillectomy in childhood, open appendectomy for complicated appendicitis without free perforation at the age of 27

Birth history: No prior birth

Oncological family history: No prior oncological family history

The patient underwent the following surgical treatment: Breast-conserving tumorectomy with sentinellymphnodectomy on the right side

Following the surgical treatment, the following TNM-classification, resection margin, histological classification, grading and immunohistology were identified:

TNM: pT1apN0MX

Resection margin: R0, minimal resection margin of 1mm

Histological classification: Mucinous

Grading: G1

Multifocality/-centricity: Monofocal and -centric

Estrogen receptor (ER): 95%

Progesterone receptor (PR): 90%

Her2-status: Negative (IHC 0)

Ki-67-proliferation-index: 8%

Please provide a step-by-step treatment recommendation with regard to further surgical treatment, endocrine treatment, systemic treatment and radiation therapy taking the given patient information in consideration.

If endocrine treatment is advisable, please provide a suitable treatment regimen.

If systemic treatment is advisable, please provide a suitable treatment regimen.

If radiation therapy is advisable, please provide a suitable treatment regimen.

Based on the oncological family history, please state whether genetic testing should be performed or not.

| **Patient 3 Treatment Concepts** |  |  |
| --- | --- | --- |
| **Concept 1** | **Concept 2** | **Concept 3** |
| **Treatment Recommendations for the Patient**  **1. Further Surgical Treatment**  - No further surgical treatment is required. The patient underwent breast-conserving surgery (BCS) with sentinel lymph node biopsy, achieving clear resection margins (R0) with a minimal margin of 1 mm. This is sufficient for a pT1a, G1, ER/PR-positive, HER2-negative, low-proliferation (Ki-67: 8%) mucinous carcinoma, which is a low-risk subtype.  **2. Endocrine Treatment**  - Endocrine therapy is strongly recommended due to the high expression of estrogen (95%) and progesterone (90%) receptors.  - Recommended Regimen:  - Tamoxifen: 20 mg daily for 5 years, as the patient is premenopausal.  - Consider ovarian function suppression (OFS) with a GnRH agonist (e.g., goserelin 3.6 mg subcutaneously every 28 days) if the patient transitions to a higher-risk category (e.g., recurrence or progression).  **3. Systemic Chemotherapy**  - Systemic chemotherapy is not recommended. The tumor is low-grade (G1), monofocal, hormone receptor-positive, HER2-negative, and has a low Ki-67 index (8%), indicating a low risk of recurrence. There is no evidence to suggest that chemotherapy would provide additional benefit in this context.  **4. Radiation Therapy**  - Radiation therapy is recommended following breast-conserving surgery to reduce the risk of local recurrence  - Recommended Regimen:  - Whole-breast irradiation (WBI) with a total dose of 40–50 Gy delivered in 15–20 fractions over 3–4 weeks.  - A tumor bed boost (10–16 Gy in 4–8 fractions) may be considered, especially given the minimal resection margin of 1 mm, though the low-risk profile of the tumor may allow omission of the boost.  **5. Genetic Testing**  - Genetic testing is not indicated. The patient has no family history of breast or other cancers, and the tumor characteristics (mucinous, low-grade, hormone receptor-positive) do not suggest a hereditary cancer syndrome.  **Summary of Recommendations**  - Surgery: No further surgical intervention.  - Endocrine Therapy: Tamoxifen 20 mg daily for 5 years.  - Systemic Therapy: No chemotherapy required.  - Radiation Therapy: Whole-breast irradiation (40–50 Gy in 15–20 fractions) with consideration of a tumor bed boost.  - Genetic Testing: Not indicated. | Here’s a practical, guideline-based plan for this patient (50, premenopausal) with a right-sided, pure mucinous, pT1a pN0, G1, ER/PR-high, HER2-negative, Ki-67 8% cancer after breast-conserving surgery and negative sentinel node.  **1) Further surgery**   - No re-excision: For invasive cancer treated with breast-conserving surgery, a negative margin defined as “no ink on tumor” is adequate; a 1 mm minimal margin meets this standard. No additional breast or axillary surgery is indicated.   **2) Adjuvant endocrine therapy (recommended)**   - Rationale: Strongly ER/PR-positive, node-negative, tiny (T1a) mucinous tumor ⇒ extremely low baseline risk; endocrine therapy provides proportional risk reduction with minimal absolute toxicity burden. Ovarian suppression is not needed in such low-risk disease (SOFT/TEXT intensification benefits were greatest in higher-risk patients). - Regimen:   - Tamoxifen 20 mg orally once daily for 5 years.   - Consider stopping at 5 years; extension beyond 5 years is generally reserved for higher-risk features (positive nodes, larger size), which are absent here. - Notes: Tamoxifen is compatible with a history of multiple sclerosis; no special contraindication is reported. Use non-hormonal contraception while on tamoxifen (teratogenic risk).   **3) Adjuvant systemic chemotherapy (not recommended)**   - No chemotherapy. Pure mucinous, node-negative, HR-positive tumors have excellent outcomes and do not show a survival benefit from adjuvant chemotherapy; genomic assays are rarely informative here and typically unnecessary.   **4) Adjuvant radiation therapy (recommended after lumpectomy)**  Younger age (≤50) after breast-conserving surgery favors radiotherapy, with a tumor-bed boost recommended to maximize local control—even though absolute benefit is small in this very low-risk biology.  Preferred options (choose one, based on local practice and patient preference):  A. Whole-breast irradiation (WBI), hypofractionated (standard worldwide)   - 40 Gy in 15 fractions (3 weeks) *or* 42.5 Gy in 16 fractions (3–3½ weeks) to the whole breast, plus a tumor-bed boost 10–16 Gy in 4–8 fractions. Hypofractionation is the preferred schedule in ASTRO guidance; boost is recommended for patients ≤50 years.   B. WBI, ultra-hypofractionated (FAST-Forward style, where implemented)   - 26 Gy in 5 daily fractions (1 week) to the whole breast; if a boost is given, add 10–12.5 Gy in 4–5 fractions sequentially (some centers offer a simultaneous integrated boost). Five-fraction WBI is non-inferior for local control at 5 years and widely adopted in the UK/Europe.   C. Accelerated partial-breast irradiation (APBI) — reasonable alternative   - Patient meets modern ASTRO “suitable/strongly consider” criteria (age ≥ 50, T1, ER+, node-negative, negative margins, favorable histology). Common external-beam APBI regimens include 30 Gy in 5 fractions delivered on non-consecutive days over ~2 weeks, or 40.05 Gy in 15 fractions. Choose APBI if patient prioritizes fewer visits and the cavity is well-defined with clips.   Fields: Breast-only; no regional nodal irradiation (pN0, T1a, no LVI). Cardiac sparing: Use DIBH for right/left as per center policy.  **5) Genetic testing**   - Offer germline testing despite a negative family history: The 2024 ASCO–SSO guideline recommends BRCA1/2 testing for all patients diagnosed at age ≤ 65 with stage I–III (or de novo IV) breast cancer; most centers implement this via a multigene panel that includes PALB2 and other high-penetrance genes. This patient is 50, so testing should be offered.   **Why this plan fits this case**   - Margins/surgery: 1 mm negative margin is sufficient in invasive disease—no benefit from wider margins. - Biology: Pure mucinous, G1, very low Ki-67, ER/PR-high, HER2-negative → excellent prognosis; chemo adds no proven benefit in node-negative mucinous cancers. - Radiation: After lumpectomy, WBI (or APBI in carefully selected patients) reduces local recurrence; modern hypofractionation is standard, and boost is recommended at age ≤50. - Endocrine: Tamoxifen alone is adequate for low-risk premenopausal disease; OFS±AI is reserved for higher-risk features per SOFT/TEXT. - Genetics: Universal testing up to age 65 is now guideline-supported, independent of family history.   **One concise “default” plan to implement**   1. No further surgery. 2. Radiation: WBI 40 Gy/15 fx + boost 10–12 Gy/4–5 fx (or 26 Gy/5 fx + sequential boost if available). 3. Endocrine: Tamoxifen 20 mg daily for 5 years (start during or after RT; either is acceptable). 4. No chemotherapy. 5. Offer germline BRCA1/2 (panel) testing with pre-test counseling.   If she prefers the fewest visits and meets planning criteria, replace step 2 with APBI 30 Gy in 5 non-consecutive fractions.  Follow-up (brief): clinical exam every 6–12 months for 5 years, then annually; annual mammography of both breasts. (Follow your local guideline schedule.) | This patient has pT1a pN0, ER/PR-positive, HER2-negative, grade 1, mucinous, monofocal breast cancer with a low Ki-67 (8%) and clear margins after breast-conserving surgery. She is premenopausal, ECOG 1, with no family history of cancer.  **Further surgical treatment:**  No additional surgery is indicated. The resection margin is negative (R0), and a 1 mm margin is considered adequate for invasive cancer after breast-conserving surgery according to current standards.  **Endocrine therapy:**  Endocrine therapy is recommended for all premenopausal women with ER-positive, HER2-negative early breast cancer, regardless of tumor size, as it significantly reduces recurrence risk. For premenopausal women, tamoxifen 20 mg daily for 5 years is standard. Ovarian suppression may be considered in higher-risk cases, but this patient’s risk is very low (T1a, G1, low Ki-67, node-negative), so tamoxifen monotherapy is appropriate. The American Society of Clinical Oncology (ASCO) also supports this approach.  **Systemic chemotherapy:**  Systemic chemotherapy is not indicated. The tumor is very small (T1a), low grade, low proliferation, and node-negative. Chemotherapy provides minimal or no benefit in this setting, as supported by the American Society of Clinical Oncology.  **Radiation therapy:**  Adjuvant whole-breast radiation therapy is recommended after breast-conserving surgery to reduce local recurrence risk, even for small, low-risk tumors, unless there are significant contraindications. Hypofractionated regimens (e.g., 40 Gy in 15 fractions) are standard.  **Genetic testing:**  Genetic testing is not indicated based on current guidelines, as there is no personal or family history suggestive of hereditary breast cancer.  **In summary:**  • No further surgery  • Tamoxifen 20 mg daily for 5 years  • No chemotherapy  • Whole-breast radiation therapy  • No genetic testing |

| **PATIENT CASE 3** |  | |  | |  |  |  |  |
| --- | --- | --- | --- | --- | --- | --- | --- | --- |
|  |  | |  | |  |  |  |  |
| **How difficult or easy was the patient case?** | **very easy** | | **easy** | | **moderate** | **difficult** | **very difficult** | **Score** |
| **Case 3** | **1** | | **2** | | **3** | **4** | **5** |  |
|  |  | |  | |  |  |  |  |
| **The therapy concept is safe.** | **strongly disagree** | | **disagree** | | **neither agree nor disagree** | **agree** | **strongly agree** | **Score** |
| **Therapy Concept 1** | **1** | | **2** | | **3** | **4** | **5** |  |
| **Therapy Concept 2** | **1** | | **2** | | **3** | **4** | **5** |  |
| **Therapy Concept 3** | **1** | | **2** | | **3** | **4** | **5** |  |
|  |  | |  | |  |  |  |  |
| **The therapy concept is in line with the current guidelines.** | **strongly disagree** | | **disagree** | | **neither agree nor disagree** | **agree** | **strongly agree** | **Score** |
| **Therapy Concept 1** | **1** | | **2** | | **3** | **4** | **5** |  |
| **Therapy Concept 2** | **1** | | **2** | | **3** | **4** | **5** |  |
| **Therapy Concept 3** | **1** | | **2** | | **3** | **4** | **5** |  |
|  |  | |  | |  |  |  |  |
| **The therapy concept is medically adequate.** | **strongly disagree** | | **disagree** | | **neither agree nor disagree** | **agree** | **strongly agree** | **Score** |
| **Therapy Concept 1** | **1** | | **2** | | **3** | **4** | **5** |  |
| **Therapy Concept 2** | **1** | | **2** | | **3** | **4** | **5** |  |
| **Therapy Concept 3** | **1** | | **2** | | **3** | **4** | **5** |  |
|  |  | |  | |  |  |  |  |
| **The therapy concept is complete.** | **strongly disagree** | | **disagree** | | **neither agree nor disagree** | **agree** | **strongly agree** | **Score** |
| **Therapy Concept 1** | **1** | | **2** | | **3** | **4** | **5** |  |
| **Therapy Concept 2** | **1** | | **2** | | **3** | **4** | **5** |  |
| **Therapy Concept 3** | **1** | | **2** | | **3** | **4** | **5** |  |
|  |  | |  | |  |  |  |  |
| **The therapy concept is of high quality.** | **strongly disagree** | | **disagree** | | **neither agree nor disagree** | **agree** | **strongly agree** | **Score** |
| **Therapy Concept 1** | **1** | | **2** | | **3** | **4** | **5** |  |
| **Therapy Concept 2** | **1** | | **2** | | **3** | **4** | **5** |  |
| **Therapy Concept 3** | **1** | | **2** | | **3** | **4** | **5** |  |
|  |  | |  | |  |  |  |  |
| **The reasoning behind the treatment decision is logical.** | **strongly disagree** | | **disagree** | | **neither agree nor disagree** | **agree** | **strongly agree** | **Score** |
| **Therapy Concept 1** | **1** | | **2** | | **3** | **4** | **5** |  |
| **Therapy Concept 2** | **1** | | **2** | | **3** | **4** | **5** |  |
| **Therapy Concept 3** | **1** | | **2** | | **3** | **4** | **5** |  |
|  |  | |  | |  |  |  |  |
| **Please rank the therapy concept according to your preference.** | | **Ranking (1^st^, 2^nd^, 3^rd^)** | |  |  |  |  |  |
| **Therapy Concept 1** | |  | |  |  |  |  |  |
| **Therapy Concept 2** | |  | |  |  |  |  |  |
| **Therapy Concept 3** | |  | |  |  |  |  |  |

**Patient 4**

How should the following breast cancer patient be treated based on the most relevant and current international literature?

Patient information:

Age: 45

Menopause status: premenopausal

ECOG: 1

Previous illness: HELLP Syndrome at first pregnancy at age of 34

Previous surgical treatment: Postpartum cardiomyopathy with intensive care ECMO support, Roux-Y gastric bypass for obesity (BMI 50) at the age of 32

Birth history: 2 cesareans at the age of 34 and 38

Oncological family history: Paternal uncle with prostate cancer at the age of 65

The patient underwent the following surgical treatment: Breast-conserving tumorectomy with sentinellymphnodectomy on the left side

Following the surgical treatment, the following TNM-classification, resection margin, histological classification, grading and immunohistology were identified:

TNM: pT1cpN1aM0

Resection margin: R1 on lateral aspect

Histological classification: NST

Grading: G2

Multifocality/-centricity: Monofocal and -centric

Estrogen receptor (ER): 100%

Progesterone receptor (PR): 100%

Her2-status: Negative (IHC 0)

Ki-67-proliferation-index: 10%

Please provide a step-by-step treatment recommendation with regard to further surgical treatment, endocrine treatment, systemic treatment and radiation therapy taking the given patient information in consideration.

If endocrine treatment is advisable, please provide a suitable treatment regimen.

If systemic treatment is advisable, please provide a suitable treatment regimen.

If radiation therapy is advisable, please provide a suitable treatment regimen.

Based on the oncological family history, please state whether genetic testing should be performed or not.

| **Patient 4 Treatment Concepts** |  |  |
| --- | --- | --- |
| **Concept 1** | **Concept 2** | **Concept 3** |
| **Treatment Recommendations for the Patient**  **1. Further Surgical Treatment**  - The resection margin is R1 (positive on the lateral aspect), indicating residual tumor at the margin. Re-excision of the lateral margin is recommended to achieve clear margins (R0). If re-excision is not feasible or the patient declines, mastectomy should be considered.  **2. Radiation Therapy**  - Postoperative radiation therapy is indicated due to the breast-conserving surgery and the presence of nodal involvement (pN1a).  - Recommended regimen:  - Whole-breast irradiation (WBI) with a total dose of 50 Gy in 25 fractions over 5 weeks.  - A tumor bed boost of 10–16 Gy in 4–8 fractions is recommended due to the close/positive margin status and the patient’s age (<50 years).  **3. Endocrine Treatment**  - The tumor is strongly hormone receptor-positive (ER 100%, PR 100%) and HER2-negative, making endocrine therapy the cornerstone of systemic treatment.  - Recommended regimen:  - Ovarian suppression with a gonadotropin-releasing hormone (GnRH) agonist (e.g., goserelin 3.6 mg subcutaneously every 28 days) to induce ovarian function suppression (OFS).  - Aromatase inhibitor (AI) (e.g., letrozole 2.5 mg daily) for 5–10 years, as AI combined with OFS is superior to tamoxifen in premenopausal women with high-risk features.  - If AI is contraindicated or not tolerated, tamoxifen 20 mg daily with OFS is an alternative.  **4. Systemic Treatment**  - Chemotherapy is not recommended in this case due to the following factors:  - Low Ki-67 proliferation index (10%), indicating low tumor proliferation.  - Strong hormone receptor positivity and HER2 negativity, suggesting a luminal A-like subtype, which is less likely to benefit from chemotherapy.  - The patient’s history of postpartum cardiomyopathy and HELLP syndrome increases the risk of chemotherapy-related cardiotoxicity and other complications.  **5. Genetic Testing**  - Genetic testing is not routinely indicated based on the provided family history. The paternal uncle’s prostate cancer at age 65 does not strongly suggest a hereditary cancer syndrome. However, if there are additional family members with cancer or if the patient expresses concern, genetic counseling may be considered to assess the need for testing.  **Summary of Treatment Plan**  1. Surgery: Re-excision of the lateral margin or mastectomy if re-excision is not feasible.  2. Radiation Therapy: Whole-breast irradiation with a tumor bed boost.  3. Endocrine Therapy: Ovarian suppression with a GnRH agonist plus an aromatase inhibitor (preferred) or tamoxifen.  4. Systemic Therapy: No chemotherapy recommended.  5. Genetic Testing: Not indicated based on current family history. | Here’s a practical, guideline-concordant plan for this 45-year-old, premenopausal woman with left-sided, ER/PR-positive, HER2-negative breast cancer after breast-conserving surgery (BCS) and SLNB, pathologic stage pT1c pN1a cM0 (G2, Ki-67 10%), with a focally positive lateral margin (R1).  **1) What to do surgically (now)**  a) Re-excise the lateral margin to achieve “no tumor on ink.” Positive margins after BCS are an indication for re-excision; the internationally accepted standard for invasive cancer is *no ink on tumor*—wider margins don’t add benefit. If achieving a clear margin would be cosmetically difficult, involve oncoplastic techniques or (if truly not feasible) discuss mastectomy with immediate reconstruction.  b) Axilla: do *not* complete an ALND if only 1–2 SLNs are positive and there’s no gross extracapsular extension. In women having BCS plus adjuvant therapy, ACOSOG Z0011 and AMAROS showed that omitting ALND (and using breast/axillary RT) yields equivalent survival and regional control with less lymphedema. If ≥3 positive nodes or gross ECE are documented, discuss ALND *or* comprehensive axillary RT.  **2) Radiotherapy (after re-excision; sequencing below)**  Because she had BCS and has node-positive disease, adjuvant radiation is indicated.  a) Fields & targets   - Whole-breast irradiation (WBI) *plus* regional nodal irradiation (RNI) (supraclavicular/axillary level III; treat level I–II with high tangents or dedicated fields). Consider internal mammary nodes particularly for medial/central tumors or higher nodal burden. Large randomized trials (MA.20, EORTC 22922) show RNI reduces recurrence and breast-cancer mortality.   b) Dose/fractionation (examples commonly used today)   - WBI ± RNI: 40 Gy in 15 fractions over 3 weeks (hypofractionated) is standard and guideline-supported, including with nodal fields in many centers. FAST-Forward (26 Gy/5 fx) is established for breast/chest wall; 5-fraction RNI is still emerging—most programs use 15–16 fractions when including nodes. - Tumor-bed boost (recommended at age 45): 10–16 Gy in 4–8 fractions. The EORTC boost trials show durable local-control benefit in younger patients.   c) Heart protection (left breast) given her cardiac history  Plan with deep-inspiration breath-hold (DIBH) and modern planning to keep mean heart dose as low as reasonably achievable (ideally ~1–2 Gy) and LAD doses minimized. DIBH consistently cuts cardiac dose vs free breathing.  **3) Endocrine theray (adjuvant)**  She is premenopausal, node-positive, strongly ER/PR-positive, and Ki-67 is low (luminal-A–like). Endocrine therapy is mandatory and the backbone of systemic treatment.  Preferred regimen (higher-risk premenopausal standard): Ovarian function suppression (OFS) + an aromatase inhibitor (AI) for 5 years.   - OFS: Goserelin 3.6 mg SC every 28 days (or 10.8 mg q12 weeks) *or* leuprolide/triptorelin on standard schedules. - AI: Letrozole 2.5 mg PO daily (or anastrozole 1 mg daily; exemestane 25 mg daily). SOFT/TEXT long-term data show OFS+AI improves disease-free outcomes vs tamoxifen±OFS in premenopausal HR+ disease, particularly for node-positive women.   Why I prefer an AI (and specifically letrozole/anastrozole) here: she previously had a Roux-Y gastric bypass, and case series plus recent PK guidance report tamoxifen malabsorption after RYGB (often yielding subtherapeutic endoxifen unless dose-escalated/monitored). By contrast, anastrozole/letrozole exposures appear adequate after RYGB (no routine dose change needed). If tamoxifen is ever used, consider CYP2D6 genotyping and endoxifen therapeutic drug monitoring.  Duration/extension: After 5 years, reassess menopause (by labs off OFS). If postmenopausal, consider AI extension for 2–5 more years based on risk/tolerance; if still premenopausal and cannot continue OFS, consider tamoxifen to complete 7–10 years total (bearing in mind the RYGB absorption issue). ESMO and contemporary reviews support individualized extension.  Bone health while on OFS+AI: Baseline DXA, calcium/vitamin D, lifestyle measures. Discuss adjuvant bisphosphonates (e.g., zoledronic acid 4 mg IV q6 months for 3 years) to support bone and modestly reduce recurrence in *treatment-induced* menopause; denosumab is not recommended for recurrence prevention.  **4) Chemotherapy (do we need it?)**  This is the single most nuanced decision.   - What guidelines/trials say: In premenopausal women with 1–3 positive nodes and a low Oncotype DX score (≤25), RxPONDER still showed a statistically significant benefit from adding chemotherapy to endocrine therapy; however, many experts believe much of that benefit reflects ovarian suppression induced by chemotherapy rather than cytotoxicity per se. - Your patient’s biology/comorbidity: T1c, G2, Ki-67 10%, and a history of postpartum cardiomyopathy (anthracycline risk). The absolute chemotherapy benefit in this luminal-A–like setting is likely small, and excellent outcomes can be achieved with optimized OFS+AI. ESMO emphasizes that Ki-67 alone shouldn’t decide chemo, but low-grade/low-proliferation features support de-escalation.   Reasonable paths to discuss:   - Path A (conservative, many NCCN/ESMO teams still choose for premenopausal N1): Adjuvant chemotherapy → then endocrine therapy (OFS+AI). If chosen, avoid anthracyclines given her cardiac history. A common, effective, non-anthracycline option is TC x4 (docetaxel 75 mg/m² + cyclophosphamide 600 mg/m² IV q21d for 4 cycles), which outperformed AC in long-term follow-up. Weekly paclitaxel x12 is another lower-cardiac-risk option. Coordinate cardio-oncology surveillance. - Path B (very defensible here, and my favored plan given low-risk biology + cardiac history): Skip chemotherapy and give OFS+AI thoroughly and early, with excellent adherence and bone/cardiac supportive care. (Have a transparent discussion that RxPONDER did show a population-level chemo benefit in premenopausal N1, likely via OFS, and you are *delivering OFS directly*.)   CDK4/6 inhibitors: She does not meet high-risk eligibility used in approvals/trials (≥4 nodes, or 1–3 nodes with tumor ≥5 cm or grade 3, etc.). Adjuvant abemaciclib (monarchE) and recently approved ribociclib (NATALEE-based approvals) target those higher-risk groups; not indicated here.  **5) Treatment sequencing**   - If no chemotherapy (Path B): complete re-excision, then start RT within ~4–8 weeks, and start OFS+AI either during RT or immediately after (practice varies). - If chemotherapy (Path A): re-excision → chemo → RT → OFS+AI. (OFS can begin during or just after RT if not started earlier.)   **6) Genetic testing—should we do it?**  Yes. ASCO–Society of Surgical Oncology’s 2024 guideline recommends offering germline BRCA1/2 testing to all patients diagnosed at age ≤65 (with multipanel testing considered to inform care). Even though the only family history is a paternal uncle with prostate cancer at 65 (not high-grade by report), her age 45 alone meets testing criteria. Order a multigene panel (at least BRCA1/2, PALB2; consider TP53 only if clinical features suggest LFS) with pre-test counseling.  **7) Practical supportive care & precautions**   - Cardio-oncology input given prior cardiomyopathy, especially before chemo and for left-sided RT planning. Use DIBH and modern constraints as noted. - Contraception: Use non-hormonal methods during endocrine therapy. - Bone health: Baseline DXA; calcium/vitamin D; weight-bearing exercise; consider zoledronic acid as above. - Thromboembolism: Another reason to prefer AI+OFS over tamoxifen in a patient with prior obesity and surgical history (plus RYGB absorption concerns).   **A concise, ready-to-order plan**   1. Re-excise lateral margin to “no ink on tumor.” Consider oncoplastic closure. 2. No ALND if only 1–2 positive SLNs and no gross ECE; proceed with RNI instead. 3. Radiation:    - WBI 40 Gy/15 fx + RNI (levels III±II, supraclavicular; consider IMN by location).    - Boost 10–16 Gy in 4–8 fx to tumor bed.    - Use DIBH for heart sparing. 4. Endocrine (start during/after RT): Goserelin q28d + letrozole 2.5 mg daily for 5 years; then reassess for extension. Manage bone health; consider zoledronic acid q6 mo × 3 y. (If tamoxifen is ever chosen, plan CYP2D6 genotyping and endoxifen TDM because of RYGB.) 5. Chemotherapy: Discuss shared decision-making. Given low proliferation (Ki-67 10%), small T1c, G2, and cardiac history, it is reasonable to omit and rely on optimized OFS+AI. If chosen, use TC x4 (non-anthracycline). 6. Genetics: Order germline BRCA1/2 (panel including PALB2 at minimum) with counseling.   **Notes on risk features you asked about**   - Ki-67 10% & G2 support a luminal-A–like biology with lower chemo sensitivity, but ESMO cautions not to use Ki-67 alone to make chemo decisions; hence the emphasis on endocrine intensification with OFS. | This patient is a 45-year-old premenopausal woman with pT1c pN1a M0, ER/PR 100%, HER2-negative, Ki-67 10%, G2, R1 margin after breast-conserving surgery and sentinel node dissection. The tumor is monofocal, monocentric, and node-positive.  **Further surgical treatment:**  Re-excision is indicated to achieve negative margins ("no ink on tumor") per consensus from the St. Gallen International Expert Panel and supported by the American Society of Clinical Oncology (ASCO) guidelines, as R1 margins are associated with increased local recurrence risk.  **Endocrine therapy:**  Adjuvant endocrine therapy is strongly recommended for all patients with ER-positive breast cancer. For premenopausal women with node-positive disease, the St. Gallen Panel and ASCO recommend ovarian function suppression (OFS) plus tamoxifen or OFS plus an aromatase inhibitor (e.g., exemestane) for higher-risk patients, which includes those with nodal involvement. Tamoxifen alone is less preferred in this risk group. The typical regimen is OFS (e.g., monthly GnRH agonist) plus tamoxifen 20 mg daily or exemestane 25 mg daily for at least 5 years.  **Systemic therapy (chemotherapy):**  Given the low Ki-67, intermediate grade, and strong hormone receptor positivity, the absolute benefit of chemotherapy is modest. The American Society of Clinical Oncology and St. Gallen Panel recommend risk-adapted use of chemotherapy, with genomic assays (e.g., Oncotype DX, MammaPrint) considered for decision-making in node-positive, ER-positive, HER2-negative disease. If clinical or genomic risk is high, adjuvant chemotherapy (anthracycline- and taxane-based) may be considered, but in this case, with low proliferation and favorable biology, chemotherapy may be omitted if genomic risk is low.  **Radiation therapy:**  Post-lumpectomy, whole-breast irradiation is standard. For node-positive disease, regional nodal irradiation (including axillary and supraclavicular nodes) is recommended per St. Gallen and ASCO guidelines. A boost to the tumor bed may be considered due to young age and close margin.  **Genetic testing:**  Genetic testing is indicated if there is a strong family history of breast, ovarian, pancreatic, or prostate cancer, especially at young ages. A paternal uncle with prostate cancer at 65 is not sufficient alone, but further family history assessment is warranted. If additional risk factors are present, testing for BRCA1/2 and other relevant genes should be considered.  **In summary**  Re-excision for negative margins, OFS plus tamoxifen or aromatase inhibitor for endocrine therapy, risk-adapted chemotherapy (likely omitted if genomic risk is low), and whole-breast plus regional nodal irradiation are recommended. Genetic testing is not strictly indicated based on current family history, but further assessment may be warranted. |

| **PATIENT CASE 4** |  | |  | |  |  |  |  |
| --- | --- | --- | --- | --- | --- | --- | --- | --- |
|  |  | |  | |  |  |  |  |
| **How difficult or easy was the patient case?** | **very easy** | | **easy** | | **moderate** | **difficult** | **very difficult** | **Score** |
| **Case 4** | **1** | | **2** | | **3** | **4** | **5** |  |
|  |  | |  | |  |  |  |  |
| **The therapy concept is safe.** | **strongly disagree** | | **disagree** | | **neither agree nor disagree** | **agree** | **strongly agree** | **Score** |
| **Therapy Concept 1** | **1** | | **2** | | **3** | **4** | **5** |  |
| **Therapy Concept 2** | **1** | | **2** | | **3** | **4** | **5** |  |
| **Therapy Concept 3** | **1** | | **2** | | **3** | **4** | **5** |  |
|  |  | |  | |  |  |  |  |
| **The therapy concept is in line with the current guidelines.** | **strongly disagree** | | **disagree** | | **neither agree nor disagree** | **agree** | **strongly agree** | **Score** |
| **Therapy Concept 1** | **1** | | **2** | | **3** | **4** | **5** |  |
| **Therapy Concept 2** | **1** | | **2** | | **3** | **4** | **5** |  |
| **Therapy Concept 3** | **1** | | **2** | | **3** | **4** | **5** |  |
|  |  | |  | |  |  |  |  |
| **The therapy concept is medically adequate.** | **strongly disagree** | | **disagree** | | **neither agree nor disagree** | **agree** | **strongly agree** | **Score** |
| **Therapy Concept 1** | **1** | | **2** | | **3** | **4** | **5** |  |
| **Therapy Concept 2** | **1** | | **2** | | **3** | **4** | **5** |  |
| **Therapy Concept 3** | **1** | | **2** | | **3** | **4** | **5** |  |
|  |  | |  | |  |  |  |  |
| **The therapy concept is complete.** | **strongly disagree** | | **disagree** | | **neither agree nor disagree** | **agree** | **strongly agree** | **Score** |
| **Therapy Concept 1** | **1** | | **2** | | **3** | **4** | **5** |  |
| **Therapy Concept 2** | **1** | | **2** | | **3** | **4** | **5** |  |
| **Therapy Concept 3** | **1** | | **2** | | **3** | **4** | **5** |  |
|  |  | |  | |  |  |  |  |
| **The therapy concept is of high quality.** | **strongly disagree** | | **disagree** | | **neither agree nor disagree** | **agree** | **strongly agree** | **Score** |
| **Therapy Concept 1** | **1** | | **2** | | **3** | **4** | **5** |  |
| **Therapy Concept 2** | **1** | | **2** | | **3** | **4** | **5** |  |
| **Therapy Concept 3** | **1** | | **2** | | **3** | **4** | **5** |  |
|  |  | |  | |  |  |  |  |
| **The reasoning behind the treatment decision is logical.** | **strongly disagree** | | **disagree** | | **neither agree nor disagree** | **agree** | **strongly agree** | **Score** |
| **Therapy Concept 1** | **1** | | **2** | | **3** | **4** | **5** |  |
| **Therapy Concept 2** | **1** | | **2** | | **3** | **4** | **5** |  |
| **Therapy Concept 3** | **1** | | **2** | | **3** | **4** | **5** |  |
|  |  | |  | |  |  |  |  |
| **Please rank the therapy concept according to your preference.** | | **Ranking (1^st^, 2^nd^, 3^rd^)** | |  |  |  |  |  |
| **Therapy Concept 1** | |  | |  |  |  |  |  |
| **Therapy Concept 2** | |  | |  |  |  |  |  |
| **Therapy Concept 3** | |  | |  |  |  |  |  |

**Patient 5**

How should the following breast cancer patient be treated based on the most relevant and current international literature?

Patient information:

Age: 62

Menopause status: postmenopausal

ECOG: 0

Previous illness: Diabetes mellitus type 1, arterial hypertension (with ACE inihibitor medication), hemorrhoids

Previous surgical treatment: Mamma abscess cleavage on the right side at the age of 35, open hemorrhoidectomy according to Milligan-Morgan at the age of 40

Birth history: 4 vaginal births at the age of 18, 20, 28 and 30

Oncological family history: Paternal uncle with colon-cancer at the age of 40, paternal grandfather with colon-cancer at the age of 60, paternal cousin with colon cancer at the age of 35

The patient underwent the following surgical treatment: Breast-conserving tumorectomy with sentinellymphnodectomy on the right side

Following the surgical treatment, the following TNM-classification, resection margin, histological classification, grading and immunohistology were identified:

TNM: pT3pN0M0

Resection margin: R0, minimal resection margin of 0.1 mm

Histological classification: Invasive-lobular

Grading: G1

Multifocality/-centricity: Monofocal and -centric

Estrogen receptor (ER): 80%

Progesterone receptor (PR): 75%

Her2-status: Negative (IHC 1+)

Ki-67-proliferation-index: 35%

Please provide a step-by-step treatment recommendation with regard to further surgical treatment, endocrine treatment, systemic treatment and radiation therapy taking the given patient information in consideration.

If endocrine treatment is advisable, please provide a suitable treatment regimen.

If systemic treatment is advisable, please provide a suitable treatment regimen.

If radiation therapy is advisable, please provide a suitable treatment regimen.

Based on the oncological family history, please state whether genetic testing should be performed or not.

| **Patient 5 Treatment Concepts** |  |  |
| --- | --- | --- |
| **Concept 1** | **Concept 2** | **Concept 3** |
| **Step-by-Step Treatment Recommendation**  **1. Further Surgical Treatment**  - No further surgical treatment is required as the patient has undergone breast-conserving surgery (BCS) with sentinel lymph node biopsy, achieving an R0 resection. The minimal resection margin of 0.1 mm is acceptable in invasive lobular carcinoma (ILC) when combined with adjuvant therapies.  **2. Endocrine Treatment**  - Given the strong hormone receptor positivity (ER: 80%, PR: 75%) and postmenopausal status, endocrine therapy is strongly recommended.  - Recommended Regimen:  - Aromatase inhibitor (AI) therapy is preferred over tamoxifen in postmenopausal women due to superior efficacy in reducing recurrence risk.  - Example regimen: Letrozole 2.5 mg orally once daily for 5 years, with consideration for extending therapy to 7–10 years based on tolerance and risk assessment.  - Bone health should be monitored due to the risk of osteoporosis with AI therapy. Baseline bone mineral density (BMD) testing and supplementation with calcium and vitamin D are advised.  **3. Systemic Treatment**  - Systemic chemotherapy is not recommended in this case due to the following factors:  - Low tumor grade (G1).  - Hormone receptor positivity and HER2 negativity.  - Absence of lymph node involvement (pN0).  - The Ki-67 index of 35% is moderately elevated but does not outweigh the low-grade and hormone receptor-positive profile.  - The patient’s comorbidities (type 1 diabetes and hypertension) further support avoiding chemotherapy unless high-risk features are present.  **4. Radiation Therapy**  - Postoperative radiation therapy (RT) is recommended following breast-conserving surgery to reduce the risk of local recurrence.  - Recommended Regimen:  - Whole-breast irradiation (WBI) with a total dose of 50 Gy delivered in 25 fractions (2 Gy per fraction) over 5 weeks.  - A tumor bed boost of 10–16 Gy in 5–8 fractions may be considered due to the close resection margin (0.1 mm).  - Modern hypofractionated regimens (e.g., 40 Gy in 15 fractions) may also be considered based on institutional protocols and patient preference.  **5. Genetic Testing**  - Genetic testing is recommended due to the significant family history of early-onset colon cancer in multiple paternal relatives, which raises suspicion for a hereditary cancer syndrome (e.g., Lynch syndrome).  - Recommended Testing:  - Germline testing for mismatch repair (MMR) gene mutations (e.g., MLH1, MSH2, MSH6, PMS2) and EPCAM.  - Consideration of BRCA1/2 testing is also warranted, as these mutations can occasionally be associated with lobular breast cancer.  **Summary of Recommendations**  1. Surgery: No further surgical intervention required.  2. Endocrine Therapy: Letrozole 2.5 mg daily for 5 years, with possible extension to 7–10 years.  3. Systemic Therapy: Chemotherapy not indicated.  4. Radiation Therapy: Whole-breast irradiation (50 Gy in 25 fractions or hypofractionated regimen) with a tumor bed boost.  5. Genetic Testing: Recommended for MMR gene mutations and BRCA1/2 testing. | Below is a practical, guideline-anchored plan tailored to this patient (62, postmenopausal; pT3 pN0 M0 invasive lobular carcinoma, G1; ER 80%, PR 75%, HER2-neg; Ki-67 ~35%; BCS + negative SLNs; margin “no tumor on ink” with the closest margin 0.1 mm).  **1) Further surgery**  No additional breast or axillary surgery is indicated. For invasive cancer treated with breast-conserving surgery (BCS) and whole-breast RT planned, “no tumor on ink” is the accepted negative margin—including for invasive lobular carcinoma; wider margins do not improve local control. Sentinel nodes are negative; no further axillary surgery is needed.  **2) Radiation therapy**  Radiation after BCS is mandatory. Use modern hypofractionation.  A. Whole-breast irradiation (WBI)   - Preferred schedules (choose one): • 40 Gy in 15 fractions (3 weeks) (START trials), or • 26 Gy in 5 daily fractions over 1 week (FAST-Forward; non-inferior local control and comparable late effects vs 40/15).   B. Tumor-bed boost   - At age 62, a boost can be individualized. Absolute gains are smaller in older patients, but a close margin (0.1 mm) is a reasonable indication to add a boost 10–16 Gy in 4–8 fractions. (Boost improves local control; the largest benefit is in younger patients, but close/positive margins also derive benefit.)   C. Regional nodal irradiation (RNI) – discuss   - She is pT3N0 (tumor >5 cm, node-negative). After lumpectomy, RNI may be considered for higher clinical-risk node-negative disease (e.g., T3), balancing modest recurrence reduction against toxicity; practice varies. If chosen, treat supraclavicular ± internal mammary nodes with a hypofractionated regimen (e.g., 40 Gy/15); FAST-Forward also reported acceptable early nodal-substudy safety. If biology and other factors are favorable (ILC G1, endocrine-sensitive, no LVSI reported), WBI alone is also reasonable. Decide in multidisciplinary RT consult.   **3) Endocrine therapy (adjuvant)**  Indicated for all HR-positive disease. Preferred initial regimen: an aromatase inhibitor (AI) for 5 years (e.g., letrozole 2.5 mg daily, anastrozole 1 mg daily, or exemestane 25 mg daily). Consider extended therapy to 7–10 years based on recurrence risk (large pT3 tumor and Ki-67 35% suggest higher late risk) and tolerance; use tools like CTS5 to individualize extension. Manage bone health (baseline DEXA; calcium/vitamin D; treat osteopenia/osteoporosis as needed).  Adjuvant bisphosphonate (recommended to discuss): For postmenopausal women, zoledronic acid 4 mg IV every 6 months for 3 years (or oral clodronate) reduces bone recurrence and improves breast cancer mortality modestly. This is widely endorsed.  **4) Cytotoxic chemotherapy (adjuvant)**  Because she is HR+/HER2-, node-negative, the standard, guideline-concordant way to decide on chemo is a multigene assay:  A. Order a genomic assay on the surgical specimen   - Oncotype DX (21-gene) or MammaPrint (70-gene)—recognized tools to guide chemotherapy in HR+/HER2-, N0 disease.   - TAILORx: Women ≥50 years with RS 0–25 had no chemo benefit; chemo reserved for RS ≥26.   - MINDACT: In clinically high-risk but genomically low-risk women (many node-negative), chemo could be omitted with small absolute benefit loss, particularly age >50.   Recommendation: • If RS ≤25 (or MammaPrint Low Risk): Endocrine therapy alone (no chemo). • If RS ≥26 (or MammaPrint High Risk): Recommend chemo, then endocrine therapy.  B. If chemotherapy is indicated, choose a regimen   - For node-negative HR+ disease, a non-anthracycline regimen is appropriate and minimizes cardiotoxicity (relevant with hypertension and type 1 diabetes):   - Docetaxel 75 mg/m² + cyclophosphamide 600 mg/m² (TC) q3 weeks × 4 cycles. - For very high genomic risk (e.g., RS ≥31) and fit patients, anthracycline-taxane (e.g., AC-T) can be considered; some contemporary analyses suggest incremental benefit over TC only in the highest scores.   Note: Invasive lobular cancers are often less chemosensitive; genomic risk helps avoid overtreatment.  **5) Targeted adjuvant options (CDK4/6 inhibitors)**   - Abemaciclib (monarchE) is for node-positive high-risk disease—not applicable here (pN0). - Ribociclib (NATALEE) + AI for 3 years showed a significant iDFS benefit in stage II–III HR+/HER2-, including node-negative patients; FDA (Sept 2024) and European Commission (Nov 2024) approved this indication for high-risk early breast cancer. A pT3N0 tumor meets stage II/high-risk definitions considered in NATALEE. Discuss eligibility, toxicities (neutropenia, LFTs, QTc) and country-specific reimbursement/access. If chosen, it is typically started with the AI in the adjuvant setting.   **6) Practical sequencing & timelines**   - If giving chemo, start within typical post-op windows; then give RT; endocrine therapy runs for years (can start before or after RT depending on local practice). Acceptable intervals between modalities are well defined.   **7) Genetics: should testing be performed?**  Yes—two separate issues:  A. Breast cancer germline testing (ASCO–SSO 2024): Offer BRCA1/2 testing to all patients ≤65 with a new stage I–III breast cancer diagnosis (consider multigene panels including PALB2 per local practice). She is 62 → testing recommended/offer. Results can influence future risk management and family counseling.  B. Lynch syndrome evaluation (family history of early colon cancers): Her pedigree includes two second-degree relatives with colorectal cancer, one <50, plus another young cousin—this meets widely used criteria to evaluate for Lynch syndrome. Refer for genetic counseling and consider MMR gene panel testing (MLH1, MSH2, MSH6, PMS2, EPCAM) guided by NCCN hereditary colorectal criteria.  **Putting it all together (one possible pathway)**   1. No re-excision or ALND. 2. Order a genomic assay (Oncotype DX or MammaPrint). Use result to decide on chemo. 3. If RS ≤25 / genomic low-risk: proceed with RT (WBI hypofractionated; consider modest boost due to very close margin; RNI individualized) → AI for 5 y, consider 7–10 y; start zoledronic acid q6 mo × 3 y. 4. If RS ≥26 / genomic high-risk: give TC ×4 (or escalate to AC-T if RS ≥31 and fit), then RT as above, then AI (± consider ribociclib with AI for 3 y if available/appropriate). 5. Genetics: offer BRCA1/2 testing and refer for Lynch evaluation.   **Notes for comorbidities & supportive care**   - Bone & metabolic health: DEXA before AI; optimize calcium/vitamin D; manage dyslipidemia and arthralgias proactively. - Chemo & diabetes: If chemo is used, plan for steroid-associated hyperglycemia around taxane days (coordinate with diabetology). - Cardiac risk: Favor non-anthracycline if chemo is needed, given HTN/diabetes; if using anthracycline, obtain baseline LVEF and monitor. - Heart-sparing RT planning (even right-sided) is routine per modern standards.   **Key sources (selection)**   - Margins/ILC: SSO–ASTRO margin guideline & recent ASBrS resource guide (“no tumor on ink,” applies to ILC). - RT after BCS & hypofractionation: EBCTCG meta-analysis; START & FAST-Forward (26 Gy/5). - Boost evidence: EORTC boost trial & summaries. - RNI considerations: PDQ summary of MA.20/EORTC 22922 effects (reduced recurrences; nuanced OS). - Genomic-guided chemo: TAILORx & MINDACT. - Adjuvant bisphosphonates: ASCO/EBCTCG-aligned guidance. - Adjuvant CDK4/6: NATALEE + EU/FDA approvals of ribociclib for stage II–III, including N0. - Genetics: ASCO–SSO 2024 BRCA testing ≤65; NCCN-aligned Lynch criteria met by family history.   **Bottom line**   - No more surgery. - Radiation: hypofractionated WBI (40/15 or 26/5); consider boost; RNI individualized. - Endocrine therapy: AI 5 y → consider extend to 7–10 y; add bisphosphonate. - Chemo only if genomic high-risk; otherwise endocrine alone. - Consider adjuvant ribociclib + AI (stage II high-risk) depending on access/patient preference. - Offer BRCA1/2 testing and refer for Lynch testing based on family history. | This postmenopausal patient with pT3pN0M0, ER/PR-positive, HER2-negative, G1, invasive lobular carcinoma after breast-conserving surgery and sentinel node dissection requires a multimodal adjuvant approach.  **Further surgical treatment:**  No further surgery is indicated, as margins are R0 (albeit close at 0.1 mm), and there is no nodal involvement. The St. Gallen International Expert Consensus supports margin status as the key determinant for re-excision, and close margins in invasive cancer do not mandate further surgery if R0 is achieved.  **Endocrine therapy:**  Adjuvant endocrine therapy is strongly indicated. The American Society of Clinical Oncology (ASCO) recommends 5–10 years of an aromatase inhibitor (e.g., anastrozole 1 mg daily, letrozole 2.5 mg daily, or exemestane 25 mg daily) for postmenopausal women with ER-positive, HER2-negative breast cancer, given the high ER/PR expression and absence of contraindications.Extended therapy beyond 5 years may be considered in higher-risk cases (large tumor size, high Ki-67), but comorbidities and tolerability should be weighed.  **Systemic therapy (chemotherapy):**  The need for adjuvant chemotherapy is individualized. ASCO guidelines recommend using genomic assays (e.g., Oncotype DX, MammaPrint) to guide chemotherapy decisions in node-negative, ER-positive, HER2-negative disease for women >50 years. Given the tumor’s size (pT3) and high Ki-67 (35%), a genomic assay should be performed if available. If the recurrence score is low, endocrine therapy alone is appropriate; if high, chemoendocrine therapy should be considered. Without genomic data, the high proliferation index and tumor size suggest a moderate-to-high risk, so chemotherapy (e.g., anthracycline/taxane-based regimen) may be reasonable.  **Radiation therapy:**  Adjuvant whole-breast irradiation is indicated after breast-conserving surgery for pT3 tumors, with consideration of a boost to the tumor bed due to close margins. Regional nodal irradiation is not routinely indicated in node-negative disease but may be considered for T3 tumors.  **Genetic testing:**  Given the strong family history of early-onset colon cancer in multiple paternal relatives, genetic counseling and testing for hereditary cancer syndromes (e.g., Lynch syndrome, BRCA1/2, and other relevant panels) is recommended.  **In summary:**  No further surgery; initiate aromatase inhibitor for at least 5 years; perform genomic assay to guide chemotherapy; deliver adjuvant whole-breast irradiation; and refer for genetic counseling/testing. |

| **PATIENT CASE 5** |  | |  | |  |  |  |  |
| --- | --- | --- | --- | --- | --- | --- | --- | --- |
|  |  | |  | |  |  |  |  |
| **How difficult or easy was the patient case?** | **very easy** | | **easy** | | **moderate** | **difficult** | **very difficult** | **Score** |
| **Case 5** | **1** | | **2** | | **3** | **4** | **5** |  |
|  |  | |  | |  |  |  |  |
| **The therapy concept is safe.** | **strongly disagree** | | **disagree** | | **neither agree nor disagree** | **agree** | **strongly agree** | **Score** |
| **Therapy Concept 1** | **1** | | **2** | | **3** | **4** | **5** |  |
| **Therapy Concept 2** | **1** | | **2** | | **3** | **4** | **5** |  |
| **Therapy Concept 3** | **1** | | **2** | | **3** | **4** | **5** |  |
|  |  | |  | |  |  |  |  |
| **The therapy concept is in line with the current guidelines.** | **strongly disagree** | | **disagree** | | **neither agree nor disagree** | **agree** | **strongly agree** | **Score** |
| **Therapy Concept 1** | **1** | | **2** | | **3** | **4** | **5** |  |
| **Therapy Concept 2** | **1** | | **2** | | **3** | **4** | **5** |  |
| **Therapy Concept 3** | **1** | | **2** | | **3** | **4** | **5** |  |
|  |  | |  | |  |  |  |  |
| **The therapy concept is medically adequate.** | **strongly disagree** | | **disagree** | | **neither agree nor disagree** | **agree** | **strongly agree** | **Score** |
| **Therapy Concept 1** | **1** | | **2** | | **3** | **4** | **5** |  |
| **Therapy Concept 2** | **1** | | **2** | | **3** | **4** | **5** |  |
| **Therapy Concept 3** | **1** | | **2** | | **3** | **4** | **5** |  |
|  |  | |  | |  |  |  |  |
| **The therapy concept is complete.** | **strongly disagree** | | **disagree** | | **neither agree nor disagree** | **agree** | **strongly agree** | **Score** |
| **Therapy Concept 1** | **1** | | **2** | | **3** | **4** | **5** |  |
| **Therapy Concept 2** | **1** | | **2** | | **3** | **4** | **5** |  |
| **Therapy Concept 3** | **1** | | **2** | | **3** | **4** | **5** |  |
|  |  | |  | |  |  |  |  |
| **The therapy concept is of high quality.** | **strongly disagree** | | **disagree** | | **neither agree nor disagree** | **agree** | **strongly agree** | **Score** |
| **Therapy Concept 1** | **1** | | **2** | | **3** | **4** | **5** |  |
| **Therapy Concept 2** | **1** | | **2** | | **3** | **4** | **5** |  |
| **Therapy Concept 3** | **1** | | **2** | | **3** | **4** | **5** |  |
|  |  | |  | |  |  |  |  |
| **The reasoning behind the treatment decision is logical.** | **strongly disagree** | | **disagree** | | **neither agree nor disagree** | **agree** | **strongly agree** | **Score** |
| **Therapy Concept 1** | **1** | | **2** | | **3** | **4** | **5** |  |
| **Therapy Concept 2** | **1** | | **2** | | **3** | **4** | **5** |  |
| **Therapy Concept 3** | **1** | | **2** | | **3** | **4** | **5** |  |
|  |  | |  | |  |  |  |  |
| **Please rank the therapy concept according to your preference.** | | **Ranking (1^st^, 2^nd^, 3^rd^)** | |  |  |  |  |  |
| **Therapy Concept 1** | |  | |  |  |  |  |  |
| **Therapy Concept 2** | |  | |  |  |  |  |  |
| **Therapy Concept 3** | |  | |  |  |  |  |  |

**Patient 6**

How should the following breast cancer patient be treated based on the most relevant and current international literature?

Patient information:

Age: 58

Menopause status: postmenopausal

ECOG: 1

Previous illness: Crohn's disease (with continuous therapy with TNF-alpha inhibitors)

Previous surgical treatment: Bowel-sparing resection for ileum stenosis at the age of 35, open appendectomy at the age of 25, longitudinal laparotomy for mechanical ileus at the age of 55

Birth history: No prior birth

Oncological family history: Maternal grandmother with breast cancer at the age of 80

The patient underwent the following surgical treatment: Breast-conserving tumorectomy with sentinellymphnodectomy on the left side

Following the surgical treatment, the following TNM-classification, resection margin, histological classification, grading and immunohistology were identified:

TNM: pT3(3)pN1aM0

Resection margin: R0, minimal resection margin of 7 mm

Histological classification: NST

Grading: G2

Multifocality/-centricity: Monocentric and multifocal, 3 foci

Estrogen receptor (ER): 75%

Progesterone receptor (PR): 90%

Her2-status: Negative (IHC 0)

Ki-67-proliferation-index: 28%

Please provide a step-by-step treatment recommendation with regard to further surgical treatment, endocrine treatment, systemic treatment and radiation therapy taking the given patient information in consideration.

If endocrine treatment is advisable, please provide a suitable treatment regimen.

If systemic treatment is advisable, please provide a suitable treatment regimen.

If radiation therapy is advisable, please provide a suitable treatment regimen.

Based on the oncological family history, please state whether genetic testing should be performed or not.

| **Patient 6 Treatment Concepts** |  |  |
| --- | --- | --- |
| **Concept 1** | **Concept 2** | **Concept 3** |
| **Treatment Recommendations for the Patient**  **1. Further Surgical Treatment**  - No additional surgical treatment is required as the patient has undergone a breast-conserving tumorectomy with sentinel lymph node dissection, achieving clear resection margins (R0) with a minimal margin of 7 mm. This is considered adequate for local control in the absence of residual disease.  **2. Endocrine Treatment**  - The tumor is strongly hormone receptor-positive (ER 75%, PR 90%), making endocrine therapy a cornerstone of treatment.  - Recommended Regimen:  - Aromatase Inhibitor (AI): Letrozole 2.5 mg orally once daily for 5–10 years, depending on tolerance and risk of recurrence.  - Rationale: Postmenopausal women with hormone receptor-positive breast cancer benefit significantly from AI therapy, which reduces the risk of recurrence by suppressing peripheral estrogen production.  - Monitoring: Bone mineral density should be monitored due to the risk of osteoporosis associated with AI therapy.  **3. Systemic Treatment**  - The tumor is classified as pT3pN1aM0, G2, with a Ki-67 index of 28%, indicating intermediate proliferative activity. The patient has multifocal disease and nodal involvement, which places her at higher risk for recurrence.  - Recommended Regimen:  - Chemotherapy: A sequential regimen of anthracycline and taxane-based chemotherapy is recommended:  -Doxorubicin (Adriamycin) 60 mg/m² IV and Cyclophosphamide 600 mg/m² IV every 3 weeks for 4 cycles (AC regimen).  - Followed by Paclitaxel 80 mg/m² IV weekly for 12 weeks or 175 mg/m² IV every 3 weeks for 4 cycles.  -Rationale: This regimen is effective in reducing recurrence risk in node-positive, hormone receptor-positive breast cancer.  - Considerations: The patient’s history of Crohn’s disease and TNF-alpha inhibitor therapy may increase the risk of gastrointestinal toxicity. Close monitoring for adverse effects is essential.  **4. Radiation Therapy**  - Radiation therapy is indicated following breast-conserving surgery to reduce the risk of local recurrence.  - Recommended Regimen:  - Whole-Breast Irradiation (WBI): 50 Gy in 25 fractions (2 Gy per fraction) over 5 weeks.  - Boost to Tumor Bed: An additional boost of 10–16 Gy in 5–8 fractions to the tumor bed, given the multifocal nature of the disease and the close resection margin.  - Regional Nodal Irradiation (RNI): Include the axillary and supraclavicular lymph nodes due to pN1a status.  - Rationale: Radiation therapy significantly reduces the risk of locoregional recurrence in patients with node-positive disease.  **5. Genetic Testing**  - Genetic testing is not routinely indicated based on the provided family history. The maternal grandmother’s breast cancer at the age of 80 is not suggestive of a hereditary breast cancer syndrome. However, if there are additional family members with breast or ovarian cancer, or if the patient expresses concern, testing for BRCA1/2 mutations or other hereditary cancer syndromes may be considered.  **Summary of Treatment Plan**  1. No further surgery is required.  2. Endocrine therapy: Letrozole 2.5 mg daily for 5–10 years.  3. Systemic chemotherapy: Sequential anthracycline and taxane-based regimen (AC-T).  4. Radiation therapy: Whole-breast irradiation with a tumor bed boost and regional nodal irradiation.  5. No genetic testing is recommended based on the current family history. | Below is a practical, guideline-based adjuvant plan for this 58-year-old, postmenopausal woman with left-sided, ER+/PR+, HER2-negative, grade 2, pT3 pN1a (1–3 macrometastatic nodes), multifocal NST breast cancer after breast-conserving surgery (R0, 7 mm).  I’m drawing on the most current international evidence and guidelines (ESMO/ASCO/ASTRO/NCCN) and large trials (RxPONDER, MINDACT, AMAROS, SENOMAC, MA.20/EORTC 22922, monarchE, NATALEE, FAST-Forward). I’ve included concrete regimens and flagged nuances for her Crohn’s disease on TNF-α inhibitors.  **1) Immediate next steps (work-up & MDT)**   1. Baseline systemic staging (if not already done). With anatomic stage IIIA (T3N1aM0), ESMO supports staging with CT chest/abdomen + bone scan *or* an FDG-PET/CT (one or the other) before adjuvant therapy. 2. Multidisciplinary tumor board: confirm the exact number of positive sentinel nodes and presence/absence of gross extracapsular extension (ECE) to finalise axillary management. 3. Consider a validated genomic assay (e.g., Oncotype DX or similar) to refine chemotherapy benefit in postmenopausal HR+/HER2-, 1–3 node-positive disease—consistent with RxPONDER (no chemo benefit for postmenopausal with RS 0–25) and long-term MINDACT results supporting de-escalation in genomically low-risk patients. (Local payer guidance varies, but clinically this is reasonable.)   **2) Further surgery / axilla**   - Breast margins: “No ink on tumor” is sufficient after BCS for invasive cancer; no re-excision needed with a 7 mm margin. - Axilla (sentinel-node positive, pN1a):   - If 1–2 macrometastatic sentinel nodes and no gross ECE: omit completion ALND and treat the axilla with regional nodal irradiation (RNI). This strategy is now backed by SENOMAC (2024, allowed T1–T3) and AMAROS (RT non-inferior to ALND, with less lymphedema).   - If ≥3 positive sentinel nodes and/or extensive ECE: discuss completion ALND vs axillary RT, but most centers still favor ALND in this scenario (plus RNI). (POSNOC is ongoing, but SENOMAC broadens omission criteria to T3 with 1–2 involved SLNs.)   **3) Radiation therapy (left breast)**  Indicated after BCS. Given T3 and pN1, deliver whole-breast irradiation (WBI) + comprehensive RNI (supraclavicular ± axillary levels II–III; include internal mammary nodes for left-sided, node-positive disease). Benefits of RNI are established by MA.20 and EORTC 22922.  Technique & dose (pragmatic options):   - Preferred in 2025 for RNI: Moderately hypofractionated schedule such as 40 Gy in 15 fractions to breast and nodes, with a tumor-bed boost 10–16 Gy in 4–8 fractions based on risk (age 58, multifocality → reasonable to boost). (Boost reduces local recurrence—EORTC boost data.) Use deep-inspiration breath-hold (DIBH) to minimize cardiac dose. - FAST-Forward (26 Gy in 5 fractions/1 week) is non-inferior for breast/chest-wall; however, robust data for 5-fraction *regional* nodal fields remain limited, so many centers reserve 5-fraction schedules for WBI alone. For this patient (RNI indicated), 40 Gy/15 is the safer evidence-based default.   Left-sided planning tips: use DIBH routinely; consider IMN coverage with DIBH to keep mean heart dose low.  **4) Endocrine therapy (must-have)**  She’s postmenopausal with ER 75% / PR 90%:  Baseline plan   - Aromatase inhibitor (AI) for at least 5 years:   - Letrozole 2.5 mg PO daily *(or* anastrozole 1 mg or exemestane 25 mg daily*). - Given node-positive, T3 disease, plan for extended endocrine therapy total 7–10 years, tailoring to tolerance and BMD. (NCCN/ESMO/ASCO endorse endocrine therapy for all HR+; extended duration for higher risk.)   Adjuvant CDK4/6 inhibitor (strongly consider):   - She meets “high-risk” criteria (T ≥ 5 cm and node-positive). Two evidence-based options:   1. Ribociclib 400 mg daily, days 1–21 q28d for 36 months + AI—approved (FDA 2024; EC 2024) based on NATALEE (stage II–III), with significant iDFS benefit. Requires baseline ECG (QTc) and LFT monitoring; lower diarrhea risk than abemaciclib. This is attractive given Crohn’s disease.   2. Abemaciclib 150 mg PO twice daily for 24 months + endocrine therapy—monarchE established durable iDFS/DRFS benefit in node-positive high-risk disease. However, diarrhea is common (often early; proactive loperamide advised). In a patient with Crohn’s, ribociclib is often preferred.   Supportive measures with AIs: baseline DEXA, calcium/vitamin D, lifestyle; see #6 for adjuvant bone agents.  **5) Chemotherapy (consider, don’t assume)**   - Use genomic profiling to decide. If an Oncotype DX RS ≤25 (postmenopausal), no proven chemo benefit per RxPONDER → proceed with endocrine ± CDK4/6 alone. - If RS >25, clear high clinical/genomic risk, or genomic testing is unavailable, offer adjuvant chemotherapy, then start endocrine therapy. MINDACT also supports selective chemo omission in genomically low-risk patients despite high clinical risk.   Chemo regimen choices (if indicated):   - Favor non-anthracycline in this patient (left-sided RT, Crohn’s/TNF-i):   - TC × 6 (Docetaxel 75 mg/m² + Cyclophosphamide 600 mg/m² q3 weeks × 6), with primary G-CSF prophylaxis. - Anthracycline/taxane regimens (e.g., AC→T) remain standards, but consider cardiac and GI risks; if chosen, optimize cardioprotection and bowel symptom monitoring.   Crohn’s & TNF-α inhibitor considerations during chemo:   - Cytotoxic chemotherapy increases infection risk; many multidisciplinary teams temporarily pause anti-TNF during chemotherapy and resume afterwards, balancing Crohn’s control vs infection risk. Formal GI/oncology co-management advised. (ECCO/AGA and recent reviews support individualized decisions; several reviews advise pausing during active cancer treatment.)   **6) Bone health / recurrence reduction**   - Discuss adjuvant bisphosphonates with all postmenopausal patients to reduce bone recurrence and modestly improve overall survival (ASCO-Ontario Health). Common options:   - Zoledronic acid 4 mg IV q6 months for 3 years *(widely used)*, or   - Oral clodronate 1600 mg/day for 2–3 years. (Check dental clearance first; monitor renal function for zoledronic acid.)   **7) Practical sequencing**   1. (If staging negative) → Adjuvant chemotherapy *if indicated by genomics/clinical risk* → then radiation (endocrine therapy may start during or right after RT) → CDK4/6 with endocrine per chosen regimen. 2. If no chemotherapy → start endocrine ± CDK4/6 promptly; radiation can run concurrently with endocrine therapy. (Do not give CDK4/6 concurrently with radiation fields that include large nodal volumes at most centers; sequence to minimize overlapping toxicities.)   **8) What about her Crohn’s disease on TNF-α inhibitors?**   - Radiation to the breast/axilla is generally well tolerated in IBD; major flare risk is linked to pelvic RT, not breast fields. There’s no clear need to stop TNF-α inhibitors for radiotherapy; coordinate with GI, but many centers continue biologics during RT. - CDK4/6 choice: Given Crohn’s, ribociclib (QT/LFT monitoring) may be better tolerated than abemaciclib (diarrhea). If abemaciclib is used, proactive antidiarrheal counseling is essential. - During cytotoxic chemotherapy (if used): multidisciplinary decision; temporary anti-TNF hold is commonly practiced to reduce infection risk, then resume when counts recover.   **9) Genetic testing**  Yes — test. ASCO/SSO 2024 recommends offering germline BRCA1/2 testing to all patients ≤65 years at diagnosis, with multigene panels (including PALB2, CHEK2, ATM, etc.) often used in practice. This matters for family risk and could influence therapy (e.g., adjuvant olaparib in certain gBRCA-mutated high-risk cases). (NCCN also expanded testing to ≤65 in 2024.)  **Concise, ready-to-order plan**   1. Confirm staging negative (CT chest/abdomen + bone scan *or* PET/CT). 2. Axilla: If 1–2 positive SLNs & no gross ECE → no ALND, proceed with RNI; if ≥3 or ECE → discuss ALND. 3. Radiation (post-BCS):    - 40 Gy/15 fx to breast + nodes (SCV ± IMNs);    - Boost 10–16 Gy/4–8 fx to tumor bed;    - Left-sided DIBH planning. 4. Endocrine therapy: Letrozole 2.5 mg daily (or AI equivalent) for ≥5 years, plan 7–10 years total if tolerated. 5. CDK4/6 inhibitor (strongly consider): Prefer ribociclib 400 mg d1-21 q28d for 36 mo + AI (Crohn’s-friendly profile); alternative abemaciclib 150 mg BID for 24 mo + ET. 6. Chemotherapy: Only if indicated by high genomic/clinical risk (e.g., Oncotype RS > 25). If given, use TC×6 with G-CSF; coordinate temporary anti-TNF hold with GI. 7. Bone health / recurrence reduction: Zoledronic acid 4 mg IV q6 mo × 3 y (or clodronate 1600 mg/day × 2–3 y), plus DEXA, Ca/Vit D. 8. Genetics: Order germline panel (BRCA1/2 ± PALB2/CHEK2/ATM…); provide counseling.   **Notes on evidence behind key choices**   - Omitting ALND with 1–2 positive SLNs when giving RNI is now supported even in T3 tumors (SENOMAC 2024); AMAROS established RT as a safe alternative to ALND. - RNI improves outcomes in node-positive disease (MA.20, EORTC 22922). - Postmenopausal, RS 0–25: No chemo benefit (RxPONDER). - Adjuvant CDK4/6: Abemaciclib (monarchE) and ribociclib (NATALEE; FDA/EC 2024) reduce recurrence risk in high-risk HR+/HER2- EBC. - Hypofractionated RT is guideline-standard; DIBH reduces heart dose for left-sided RT. - Adjuvant bisphosphonates should be discussed with all postmenopausal patients. - Genetic testing is recommended for all ≤65-year patients at diagnosis (ASCO/SSO 2024; NCCN aligned). | This postmenopausal patient with pT3(3)pN1aM0, ER+/PR+, HER2-negative, G2, multifocal breast cancer after breast-conserving surgery and sentinel node dissection requires a multimodal adjuvant approach.  **Further surgical treatment:**  No further breast or axillary surgery is indicated, as margins are negative (R0, 7 mm) and only 1-3 nodes are positive. Axillary lymph node dissection is not routinely required in this setting, per consensus and as reviewed in the medical literature.  **Endocrine therapy:**  Adjuvant endocrine therapy is strongly indicated. For postmenopausal women, an aromatase inhibitor (e.g., letrozole, anastrozole, or exemestane) is preferred over tamoxifen due to superior efficacy, especially in node-positive disease. Standard duration is at least 5 years, with consideration for up to 10 years in higher-risk cases. The American Society of Clinical Oncology (ASCO) recommends aromatase inhibitors as first-line adjuvant endocrine therapy in postmenopausal women with ER+/HER2- breast cancer.  **Systemic therapy:**  Adjuvant chemotherapy is recommended due to high-risk features: T3 tumor, nodal involvement, multifocality, and elevated Ki-67 (28%). Standard regimens include anthracycline- and taxane-based combinations (e.g., doxorubicin/cyclophosphamide followed by paclitaxel). The American Society of Clinical Oncology supports chemotherapy in node-positive, high-risk, ER+/HER2- disease. Given the patient’s Crohn’s disease and ongoing TNF-α inhibitor therapy, multidisciplinary evaluation is essential to balance chemotherapy risks.  **Radiation therapy:**  Adjuvant whole-breast irradiation is indicated after breast-conserving surgery, with additional boost to the tumor bed. Regional nodal irradiation (including axilla and supraclavicular fossa) should be considered due to nodal involvement and T3 status.  **Additional systemic options:**  For high-risk, ER+/HER2- early breast cancer, adjuvant CDK4/6 inhibitors (e.g., abemaciclib) may be considered in addition to endocrine therapy, as supported by recent evidence.  **Genetic testing:**  Genetic testing is not routinely indicated based on this family history (one grandmother with breast cancer at age 80), as per current guidelines. Testing is reserved for patients with stronger family histories or early-onset disease.  All recommendations should be tailored to comorbidities and patient preferences, with multidisciplinary input. |

| **PATIENT CASE 6** |  |  |  |  |  |  |
| --- | --- | --- | --- | --- | --- | --- |
|  |  |  |  |  |  |  |
| **How difficult or easy was the patient case?** | **very easy** | **easy** | **moderate** | **difficult** | **very difficult** | **Score** |
| **Case 6** | **1** | **2** | **3** | **4** | **5** |  |
|  |  |  |  |  |  |  |
| **The therapy concept is safe.** | **strongly disagree** | **disagree** | **neither agree nor disagree** | **agree** | **strongly agree** | **Score** |
| **Therapy Concept 1** | **1** | **2** | **3** | **4** | **5** |  |
| **Therapy Concept 2** | **1** | **2** | **3** | **4** | **5** |  |
| **Therapy Concept 3** | **1** | **2** | **3** | **4** | **5** |  |
|  |  |  |  |  |  |  |
| **The therapy concept is in line with the current guidelines.** | **strongly disagree** | **disagree** | **neither agree nor disagree** | **agree** | **strongly agree** | **Score** |
| **Therapy Concept 1** | **1** | **2** | **3** | **4** | **5** |  |
| **Therapy Concept 2** | **1** | **2** | **3** | **4** | **5** |  |
| **Therapy Concept 3** | **1** | **2** | **3** | **4** | **5** |  |
|  |  |  |  |  |  |  |
| **The therapy concept is medically adequate.** | **strongly disagree** | **disagree** | **neither agree nor disagree** | **agree** | **strongly agree** | **Score** |
| **Therapy Concept 1** | **1** | **2** | **3** | **4** | **5** |  |
| **Therapy Concept 2** | **1** | **2** | **3** | **4** | **5** |  |
| **Therapy Concept 3** | **1** | **2** | **3** | **4** | **5** |  |
|  |  |  |  |  |  |  |
| **The therapy concept is complete.** | **strongly disagree** | **disagree** | **neither agree nor disagree** | **agree** | **strongly agree** | **Score** |
| **Therapy Concept 1** | **1** | **2** | **3** | **4** | **5** |  |
| **Therapy Concept 2** | **1** | **2** | **3** | **4** | **5** |  |
| **Therapy Concept 3** | **1** | **2** | **3** | **4** | **5** |  |
|  |  |  |  |  |  |  |
| **The therapy concept is of high quality.** | **strongly disagree** | **disagree** | **neither agree nor disagree** | **agree** | **strongly agree** | **Score** |
| **Therapy Concept 1** | **1** | **2** | **3** | **4** | **5** |  |
| **Therapy Concept 2** | **1** | **2** | **3** | **4** | **5** |  |
| **Therapy Concept 3** | **1** | **2** | **3** | **4** | **5** |  |
|  |  |  |  |  |  |  |
| **The reasoning behind the treatment decision is logical.** | **strongly disagree** | **disagree** | **neither agree nor disagree** | **agree** | **strongly agree** | **Score** |
| **Therapy Concept 1** | **1** | **2** | **3** | **4** | **5** |  |
| **Therapy Concept 2** | **1** | **2** | **3** | **4** | **5** |  |
| **Therapy Concept 3** | **1** | **2** | **3** | **4** | **5** |  |
|  |  |  |  |  |  |  |
| **Please select your preferred therapy concept (one choice)** | **Score** |  |  |  |  |  |
| **Therapy Concept 1** |  |  |  |  |  |  |
| **Therapy Concept 2** |  |  |  |  |  |  |
| **Therapy Concept 3** |  |  |  |  |  |  |

**Patient 7**

How should the following breast cancer patient be treated based on the most relevant and current international literature?

Patient information:

Age: 40

Menopause status: premenopausal

ECOG: 0

Previous illness: Deep vein thrombosis at age 25 while on contraceptive medication, heterozygous factor V Leiden

Previous surgical treatment: Open appendectomy at the age of 28

Birth history: 1 vaginal birth at the age of 39

Oncological family history: Sister-in-law with breast cancer at the age of 30

The patient underwent the following surgical treatment: Breast-conserving tumorectomy with sentinellymphnodectomy on the left side

Following the surgical treatment, the following TNM-classification, resection margin, histological classification, grading and immunohistology were identified:

TNM: pT2pN0M0

Resection margin: R1 on lateral aspect

Histological classification: Tubular

Grading: G2

Multifocality/-centricity: Monofocal and -centric

Estrogen receptor (ER): 90%

Progesterone receptor (PR): 50%

Her2-status: Negative (IHC 2+, ISH negative)

Ki-67-proliferation-index: 30%

Please provide a step-by-step treatment recommendation with regard to further surgical treatment, endocrine treatment, systemic treatment and radiation therapy taking the given patient information in consideration.

If endocrine treatment is advisable, please provide a suitable treatment regimen.

If systemic treatment is advisable, please provide a suitable treatment regimen.

If radiation therapy is advisable, please provide a suitable treatment regimen.

Based on the oncological family history, please state whether genetic testing should be performed or not.

| **Patient 7 Treatment Concepts** |  |  |
| --- | --- | --- |
| **Concept 1** | **Concept 2** | **Concept 3** |
| **Step-by-Step Treatment Recommendation**  **1. Further Surgical Treatment**  - The resection margin is R1 (positive margin) on the lateral aspect, indicating residual tumor presence. Re-excision of the tumor bed is recommended to achieve clear margins (R0 resection). If clear margins cannot be achieved with re-excision, mastectomy should be considered.  **2. Endocrine Treatment**  - The tumor is strongly hormone receptor-positive (ER 90%, PR 50%) and HER2-negative. Endocrine therapy is indicated.  - Recommended Regimen:  - Premenopausal Status: Start with ovarian function suppression (OFS) using a gonadotropin-releasing hormone (GnRH) agonist (e.g., goserelin 3.6 mg subcutaneously every 28 days).  - Combine OFS with tamoxifen (20 mg orally daily) for 5–10 years, depending on risk assessment and tolerance.  - Alternatively, OFS can be combined with an aromatase inhibitor (e.g., letrozole 2.5 mg orally daily) if the patient is at high risk and can tolerate the regimen.  **3. Systemic Treatment**  - The tumor is HER2-negative, hormone receptor-positive, and Ki-67 is 30%, indicating intermediate proliferative activity. The patient is node-negative (pN0) and premenopausal. Based on current guidelines:  - Adjuvant Chemotherapy: May be considered due to the intermediate Ki-67 index and the patient's age (40 years). A genomic assay (e.g., Oncotype DX or MammaPrint) could help refine the decision for chemotherapy. If genomic testing is unavailable or not feasible:  - Recommended Regimen: Anthracycline and taxane-based chemotherapy, such as:  - Doxorubicin (60 mg/m²) and cyclophosphamide (600 mg/m²) every 3 weeks for 4 cycles, followed by paclitaxel (80 mg/m² weekly for 12 weeks) or docetaxel (75 mg/m² every 3 weeks for 4 cycles).  - Consider prophylactic anticoagulation during chemotherapy due to the history of deep vein thrombosis and heterozygous factor V Leiden mutation.  **4. Radiation Therapy**  - Radiation therapy is indicated following breast-conserving surgery to reduce the risk of local recurrence.  - Recommended Regimen:  - Whole-breast irradiation (WBI) with a total dose of 50 Gy in 25 fractions (2 Gy per fraction) over 5 weeks.  - A tumor bed boost of 10–16 Gy in 4–8 fractions should be added due to the positive resection margin (R1) and the patient's young age.  **5. Genetic Testing**  - The patient has a significant family history of breast cancer (sister-in-law diagnosed at age 30). Although the sister-in-law is not a first-degree relative, the early onset of breast cancer warrants consideration of genetic testing.  - Recommendation: Offer genetic counseling and testing for BRCA1/BRCA2 mutations and other hereditary breast cancer syndromes (e.g., PALB2, TP53, CHEK2), especially given the patient's young age and family history.  **Summary of Treatment Plan**  1. Surgery: Re-excision to achieve R0 margins; consider mastectomy if R0 cannot be achieved.  2. Endocrine Therapy: OFS with tamoxifen or an aromatase inhibitor.  3. Systemic Therapy: Consider adjuvant chemotherapy (anthracycline and taxane-based) based on risk assessment or genomic testing.  4. Radiation Therapy: Whole-breast irradiation with a tumor bed boost.  5. Genetic Testing: Recommended due to family history of early-onset breast cancer. | Here’s a clear, guideline-anchored plan for this 40-year-old, premenopausal woman with left-sided, ER-positive/HER2-negative, pT2 pN0 tubular carcinoma after breast-conserving surgery (BCS) and an R1 lateral margin.  **1) Further surgery**   - Re-excise the lateral margin to achieve negative margins (“no ink on tumor”). This is the international standard after BCS for invasive cancer. If negative margins cannot be achieved with acceptable cosmesis, discuss mastectomy. - Axilla: Sentinel nodes are negative—no further axillary surgery is indicated.   **2) Radiation therapy (after re-excision, if BCS is maintained)**   - Whole-breast irradiation (WBI) is indicated after BCS. Preferred hypofractionated options:   - 40 Gy in 15 fractions over 3 weeks or 26 Gy in 5 fractions over 1 week (FAST-Forward, non-inferior). - Tumor-bed boost is recommended (age <50 and prior positive margin). Typical choices: 10–16 Gy in 4–8 fractions (e.g., 16 Gy/8 as in EORTC). - Left-sided heart sparing: plan with deep inspiration breath-hold (DIBH) to reduce mean heart dose. - Regional nodal irradiation: not routine in pT2N0 without additional high-risk features. Treat the breast only. (Nodal RT is generally reserved for node-positive or select very high-risk cases.)   **3) Endocrine therapy (adjuvant)**  Given strong ER/PR expression, premenopausal status, and a history of DVT with heterozygous factor V Leiden, avoid tamoxifen (its VTE risk is substantially higher and further amplified by FVL). Use ovarian suppression + aromatase inhibitor instead.  Recommended regimen   - Ovarian function suppression (OFS) with a GnRH agonist (e.g., goserelin 3.6 mg SC q4 weeks or 10.8 mg q12 weeks, or leuprolide 3.75 mg IM q4 weeks) plus an aromatase inhibitor (e.g., exemestane 25 mg PO daily). Continue for 5 years, then reassess menopausal status and recurrence risk to decide on extension (see below). Long-term SOFT/TEXT follow-up shows exemestane+OFS improves disease-free and distant recurrence-free survival versus tamoxifen+OFS. - Bone health & anticancer adjuvant effect: obtain baseline DEXA; calcium/vitamin D, weight-bearing exercise. Because OFS renders patients functionally postmenopausal, discuss adjuvant bisphosphonate (e.g., zoledronic acid 4 mg IV q6 months for 3 years or oral clodronate/ibandronate) to reduce bone loss and (in postmenopausal status) lower bone recurrence/mortality risk. - Contraception: OFS is not contraception; use a non-hormonal method during therapy (e.g., copper IUD). - Duration beyond 5y: If she is postmenopausal at year 5, consider AI extension for 2–5 additional years (total 7–10y) based on risk/tolerance; if still premenopausal and tamoxifen is contraindicated (VTE), many clinicians stop at 5y of OFS+AI or individualize extension with continued OFS+AI if risk justifies it.   **4) Cytotoxic chemotherapy (adjuvant)**  This is not automatic in node-negative HR+/HER2- disease and tubular histology (typically favorable). Use a validated genomic assay to guide the decision.  How to decide   - Order Oncotype DX (preferred by major guidelines for node-negative HR+/HER2- disease) or an equivalent genomic test (e.g., MammaPrint). - If Oncotype DX Recurrence Score (RS):   - ≤15 → Endocrine therapy alone.   - 16–20 → In women ≤50, chemo benefit is small and may largely reflect ovarian suppression; with planned OFS+AI, many omit chemo. Shared decision-making.   - 21–25 → Consider chemotherapy (especially if other high-risk features). Benefit exists in younger women, though some may derive similar benefit from optimal OFS; discuss pros/cons.   - ≥26 → Recommend chemotherapy.  (TAILORx/RxPONDER/MINDACT collectively support genomics-guided use of chemo and highlight that much of the apparent benefit in premenopausal women can be due to ovarian suppression.)   If chemotherapy is chosen, suitable regimens include:   - TC ×4: Docetaxel 75 mg/m² + Cyclophosphamide 600 mg/m² IV q3 weeks ×4 cycles (non-anthracycline; proven superior to AC×4). - Dose-dense AC→T: Doxorubicin 60 mg/m² + Cyclophosphamide 600 mg/m² q2 weeks ×4, then Paclitaxel 175 mg/m² q2 weeks ×4 with G-CSF support (for higher-risk biology when chemotherapy is clearly indicated). - VTE note: ambulatory prophylactic anticoagulation is not routinely recommended in breast cancer patients on outpatient chemo unless overall VTE risk is very high; maintain vigilance given her history.   **5) Special notes on histology/biology**   - Tubular carcinoma generally has excellent prognosis; chemotherapy is often unnecessary in node-negative disease unless genomics/clinical factors indicate high risk. Your plan to use genomics before committing to chemo fits current evidence.   **6) Genetics**   - At age 40, she meets modern criteria for germline testing. Current ASCO–SSO guidelines (2024) recommend offering BRCA1/2 testing to all newly diagnosed patients ≤65 years, with additional genes as appropriate. Use a multigene panel that typically includes BRCA1, BRCA2, PALB2 (and, depending on local practice and history, CHEK2, ATM, etc.). Results may affect future risk management and (if positive) surgical discussions.   **Putting it all together (practical sequence)**   1. Re-excision of lateral margin to R0 (no ink). 2. Adjuvant WBI (40 Gy/15 fx or 26 Gy/5 fx) + boost to tumor bed; plan DIBH (left-sided). 3. Adjuvant endocrine therapy: OFS + exemestane for 5 years; baseline DEXA and start adjuvant zoledronic acid if no contraindication. Avoid tamoxifen. 4. Order Oncotype DX (or equivalent). If RS drives toward chemo (especially ≥26), choose TC×4 or ddAC→T, then resume OFS+AI afterward. If RS low/intermediate, proceed with OFS+AI only. 5. Genetic counseling + germline testing (BRCA1/2 ± panel including PALB2, etc.).   **Regimen details (ready toprescribe)**   - OFS + AI (preferred):   - Goserelin 3.6 mg SC every 28 days (or 10.8 mg q12 weeks) + Exemestane 25 mg PO daily for 5 years, then reassess for extension based on menopausal status/risk. Add bone protection as below. - Bone agent: Zoledronic acid 4 mg IV every 6 months for 3 years (ensure dental clearance, renal function). - If chemotherapy indicated:   - TC ×4 (Docetaxel 75 mg/m² + Cyclophosphamide 600 mg/m² IV day 1, q21 days ×4) or   - ddAC→T (AC q14 days ×4 → Paclitaxel q14 days ×4 with G-CSF).   **Why this plan fits *this* patient**   - Margin-positive BCS → re-excision per consensus (“no ink”). - Young, node-negative, ER+/HER2- → WBI+boost and genomics-guided chemo decision; many such patients safely omit chemo when genomic risk is low. - High VTE risk (prior DVT, factor V Leiden) → avoid tamoxifen; OFS+AI gives superior disease-control vs tamoxifen-based regimens without increasing VTE risk. - Left-sided RT → DIBH to minimize cardiac dose. - Age 40 → germline testing indicated under current ASCO–SSO recommendations. | **Patient and Tumor Characteristics:**  This is a 40-year-old premenopausal woman (ECOG 0) with a history of deep vein thrombosis (DVT) at age 25 while on contraceptives and heterozygous factor V Leiden. She has undergone breast-conserving tumorectomy with sentinel lymph node dissection for a left-sided, monofocal, pT2pN0M0, tubular, grade 2, ER 90%, PR 50%, HER2-negative (IHC 2+, ISH negative), Ki-67 30% tumor. The resection margin is R1 (positive) on the lateral aspect. Family history includes a sister-in-law with breast cancer at age 30.  **Further Surgical Management:**  Re-excision of the involved margin is indicated to achieve negative margins (R0), as positive margins after breast-conserving surgery are associated with increased local recurrence risk. If negative margins cannot be achieved with re-excision, mastectomy should be considered. This approach aligns with current surgical standards for breast-conserving therapy.  **Adjuvant Endocrine Therapy:**  Adjuvant endocrine therapy is strongly recommended for premenopausal women with ER-positive, HER2-negative breast cancer. Tamoxifen 20 mg daily for 5–10 years is standard. For higher-risk features (pT2, Ki-67 30%), ovarian function suppression (OFS) plus tamoxifen or an aromatase inhibitor (AI) (e.g., exemestane 25 mg daily) may be considered, as this combination further reduces recurrence risk in premenopausal women with higher-risk disease. The choice between tamoxifen alone and OFS plus tamoxifen/AI should be individualized based on risk, tolerability, and patient preference.  **Adjuvant Systemic Therapy:**  Given the tumor is node-negative but pT2 with a high Ki-67 (30%), this patient is at intermediate to high risk for recurrence. In premenopausal women, the benefit of chemotherapy is greater, especially with high-risk features. Adjuvant chemotherapy should be considered; the decision can be further refined by a genomic assay (e.g., Oncotype DX), which is validated for node-negative, ER+/HER2- premenopausal patients and can help avoid overtreatment. If a high-risk genomic score is found, chemotherapy is indicated. Standard regimens include anthracycline- and/or taxane-based protocols. CDK4/6 inhibitors (e.g., abemaciclib) may be considered in the adjuvant setting for very high-risk, HR+/HER2- disease, but are not standard for node-negative tumors.  **Radiation Therapy:**  Adjuvant whole-breast radiotherapy is indicated after breast-conserving surgery, especially with an R1 margin and a Ki-67 >13.25% (luminal B phenotype). Standard fractionation is 40–50 Gy in 15–25 fractions, with a tumor bed boost (10–16 Gy) due to the positive margin and higher risk of local recurrence. Omission of radiotherapy is not appropriate in this setting.  **Genetic Testing:**  Genetic counseling and testing are recommended due to the young age at diagnosis (<45 years) and a family history of early-onset breast cancer, even though the affected relative is a sister-in-law. Testing should include BRCA1, BRCA2, and other relevant breast cancer susceptibility genes.  If a genomic assay is not available, risk stratification is less precise, and decisions should be based on clinical and pathological features.  **Summary Table**  Further Surgery: Re-excision to achieve negative margins (R0)  Endocrine Therapy: Tamoxifen 20 mg daily × 5–10 yrs; consider OFS + tamoxifen/AI for high risk  Systemic Therapy: Consider adjuvant chemotherapy; use genomic assay to guide decision  Radiation Therapy: Whole-breast RT (40–50 Gy/15–25 fx) + boost (10–16 Gy)  Genetic Testing: Recommend genetic counseling/testing (BRCA1/2, others) |

| **PATIENT CASE 7** |  | |  | |  |  |  |  |
| --- | --- | --- | --- | --- | --- | --- | --- | --- |
|  |  | |  | |  |  |  |  |
| **How difficult or easy was the patient case?** | **very easy** | | **easy** | | **moderate** | **difficult** | **very difficult** | **Score** |
| **Case 7** | **1** | | **2** | | **3** | **4** | **5** |  |
|  |  | |  | |  |  |  |  |
| **The therapy concept is safe.** | **strongly disagree** | | **disagree** | | **neither agree nor disagree** | **agree** | **strongly agree** | **Score** |
| **Therapy Concept 1** | **1** | | **2** | | **3** | **4** | **5** |  |
| **Therapy Concept 2** | **1** | | **2** | | **3** | **4** | **5** |  |
| **Therapy Concept 3** | **1** | | **2** | | **3** | **4** | **5** |  |
|  |  | |  | |  |  |  |  |
| **The therapy concept is in line with the current guidelines.** | **strongly disagree** | | **disagree** | | **neither agree nor disagree** | **agree** | **strongly agree** | **Score** |
| **Therapy Concept 1** | **1** | | **2** | | **3** | **4** | **5** |  |
| **Therapy Concept 2** | **1** | | **2** | | **3** | **4** | **5** |  |
| **Therapy Concept 3** | **1** | | **2** | | **3** | **4** | **5** |  |
|  |  | |  | |  |  |  |  |
| **The therapy concept is medically adequate.** | **strongly disagree** | | **disagree** | | **neither agree nor disagree** | **agree** | **strongly agree** | **Score** |
| **Therapy Concept 1** | **1** | | **2** | | **3** | **4** | **5** |  |
| **Therapy Concept 2** | **1** | | **2** | | **3** | **4** | **5** |  |
| **Therapy Concept 3** | **1** | | **2** | | **3** | **4** | **5** |  |
|  |  | |  | |  |  |  |  |
| **The therapy concept is complete.** | **strongly disagree** | | **disagree** | | **neither agree nor disagree** | **agree** | **strongly agree** | **Score** |
| **Therapy Concept 1** | **1** | | **2** | | **3** | **4** | **5** |  |
| **Therapy Concept 2** | **1** | | **2** | | **3** | **4** | **5** |  |
| **Therapy Concept 3** | **1** | | **2** | | **3** | **4** | **5** |  |
|  |  | |  | |  |  |  |  |
| **The therapy concept is of high quality.** | **strongly disagree** | | **disagree** | | **neither agree nor disagree** | **agree** | **strongly agree** | **Score** |
| **Therapy Concept 1** | **1** | | **2** | | **3** | **4** | **5** |  |
| **Therapy Concept 2** | **1** | | **2** | | **3** | **4** | **5** |  |
| **Therapy Concept 3** | **1** | | **2** | | **3** | **4** | **5** |  |
|  |  | |  | |  |  |  |  |
| **The reasoning behind the treatment decision is logical.** | **strongly disagree** | | **disagree** | | **neither agree nor disagree** | **agree** | **strongly agree** | **Score** |
| **Therapy Concept 1** | **1** | | **2** | | **3** | **4** | **5** |  |
| **Therapy Concept 2** | **1** | | **2** | | **3** | **4** | **5** |  |
| **Therapy Concept 3** | **1** | | **2** | | **3** | **4** | **5** |  |
|  |  | |  | |  |  |  |  |
| **Please rank the therapy concept according to your preference.** | | **Ranking (1^st^, 2^nd^, 3^rd^)** | |  |  |  |  |  |
| **Therapy Concept 1** | |  | |  |  |  |  |  |
| **Therapy Concept 2** | |  | |  |  |  |  |  |
| **Therapy Concept 3** | |  | |  |  |  |  |  |

**Patient 8**

How should the following breast cancer patient be treated based on the most relevant and current international literature?

Patient information:

Age: 35

Menopause status: premenopausal

ECOG: 0

Previous illness: Colitis ulcerosa, Hashimoto-thyreoiditis (with L-thyroxine medication)

Previous surgical treatment: Laparoscopy for cyst extirpation of left ovarian cyst at age 30

Birth history: No prior birth

Oncological family history: Paternal grandmother with breast cancer at the age of 70, paternal aunt with breast cancer at the age of 50, maternal uncle with pancreatic cancer at the age of 60

The patient underwent the following surgical treatment: Breast-conserving tumorectomy with sentinellymphnodectomy on the right side

Following the surgical treatment, the following TNM-classification, resection margin, histological classification, grading and immunohistology were identified:

TNM: pT2pN1cM0

Resection margin: R0, minimal resection margin of 2 mm

Histological classification: Invasive-lobular

Grading: G3

Multifocality/-centricity: Monofocal and -centric

Estrogen receptor (ER): 75%

Progesterone receptor (PR): 75%

Her2-status: Positive (IHC 3+)

Ki-67-proliferation-index: 40%

Please provide a step-by-step treatment recommendation with regard to further surgical treatment, endocrine treatment, systemic treatment and radiation therapy taking the given patient information in consideration.

If endocrine treatment is advisable, please provide a suitable treatment regimen.

If systemic treatment is advisable, please provide a suitable treatment regimen.

If radiation therapy is advisable, please provide a suitable treatment regimen.

Based on the oncological family history, please state whether genetic testing should be performed or not.

| **Patient 8 Treatment Concepts** |  |  |
| --- | --- | --- |
| **Concept 1** | **Concept 2** | **Concept 3** |
| **Treatment Recommendations for the Patient**  **1. Surgical Treatment**  - No further surgical treatment is required as the patient has undergone breast-conserving surgery (tumorectomy) with sentinel lymph node dissection, achieving clear resection margins (R0, 2 mm).  **2. Systemic Treatment**  Given the patient's high-risk features (pT2, N1, G3, HER2-positive, high Ki-67 index, and premenopausal status), systemic therapy is strongly recommended.  **2.1. Chemotherapy**  - Regimen: A combination of anthracycline and taxane-based chemotherapy is recommended, such as:  - Dose-dense AC-T: Doxorubicin (Adriamycin) and Cyclophosphamide followed by Paclitaxel (Taxol) or Docetaxel.  - Duration: 4 cycles of AC followed by 4 cycles of T (total 6–8 cycles).  - HER2-targeted therapy should be initiated concurrently with chemotherapy.  **2.2. HER2-Targeted Therapy**  - Regimen: Dual HER2 blockade with Trastuzumab (Herceptin) and Pertuzumab (Perjeta) is recommended.  - Trastuzumab: Administered every 3 weeks for a total duration of 1 year.  - Pertuzumab: Administered during the chemotherapy phase.  - If residual disease is detected post-neoadjuvant chemotherapy, consider T-DM1 (trastuzumab emtansine) as adjuvant therapy.  **3. Endocrine Treatment**  - As the tumor is hormone receptor-positive (ER 75%, PR 75%), endocrine therapy is indicated after the completion of chemotherapy and HER2-targeted therapy.  - Regimen:  - Ovarian suppression: Gonadotropin-releasing hormone (GnRH) agonist such as Goserelin (Zoladex) to induce ovarian suppression.  - Aromatase inhibitor: Letrozole (preferred in premenopausal women with ovarian suppression) for 5–10 years.  - Alternatively, Tamoxifen can be considered if ovarian suppression is not feasible.  **4. Radiation Therapy**  - Radiation therapy is indicated following breast-conserving surgery.  - Regimen:  - Whole-breast irradiation (WBI) with a total dose of 50 Gy in 25 fractions over 5 weeks.  - A tumor bed boost of 10–16 Gy in 4–8 fractions is recommended due to the high-grade tumor (G3).  **5. Genetic Testing**  - Based on the family history of breast cancer in a paternal aunt (age 50) and paternal grandmother (age 70), as well as pancreatic cancer in a maternal uncle, genetic testing is recommended.  - Testing: BRCA1/BRCA2 and other hereditary breast and ovarian cancer (HBOC) panel genes should be evaluated.  **Summary of Treatment Plan**  1. Systemic Therapy:  - Chemotherapy: Dose-dense AC-T.  - HER2-targeted therapy: Trastuzumab + Pertuzumab.  2. Endocrine Therapy:  - Ovarian suppression with GnRH agonist + Aromatase inhibitor.  3. Radiation Therapy:  - Whole-breast irradiation with tumor bed boost.  4. Genetic Testing:  - BRCA1/BRCA2 and HBOC panel testing.  This comprehensive approach addresses the patient's high-risk features and optimizes long-term outcomes. | Below is a concise, evidence-based adjuvant plan for this patient (35, premenopausal; pT2 pN1 cM0; invasive lobular, G3; ER/PR 75%+, HER2 IHC 3+, Ki-67 40%; BCS + SLNB; R0 margin 2 mm).  **1) Further surgery**   - Breast: No re-excision. For invasive cancer, “no tumor on ink” is an adequate margin (your 2 mm margin already exceeds this). - Axilla:   - If 1–2 positive sentinel nodes and the case met Z0011 criteria (T1–2 cN0 at presentation, lumpectomy, planned whole-breast RT, systemic therapy), omit completion ALND.   - If ≥3 positive nodes or gross extracapsular extension, treat the axilla with axillary RT rather than ALND to minimize lymphedema (AMAROS).   **2) Systemic therapy (chemo + anti-HER2)**  Given node-positive, HR+/HER2+ disease and upfront surgery, use adjuvant combination chemo + anti-HER2, then complete 1 year of anti-HER2:  Preferred regimen (anthracycline-free):   - TCHP x 6 cycles q3w: Docetaxel 75 mg/m² + Carboplatin AUC 6 + Trastuzumab + Pertuzumab (IV), then continue trastuzumab + pertuzumab to complete 12 months total anti-HER2 (IV or the fixed-dose SC combo PHESGO). Rationale: excellent efficacy with lower cardiotoxicity vs anthracycline regimens, and additional benefit of pertuzumab in node-positive disease (APHINITY long-term).   - If using PHESGO (SC fixed-dose pertuzumab/trastuzumab): load 1200/600 mg, then 600/600 mg q3w to finish 1 year of anti-HER2.   Monitoring/notes   - Cardiac: Echo/MUGA at baseline and q3 months during anti-HER2; follow label-based thresholds for holding/resuming. - Fertility: Offer GnRH agonist (e.g., goserelin) during chemotherapy to reduce premature ovarian insufficiency and improve post-treatment pregnancy rates; safe oncologically. - IBD (ulcerative colitis): Expect diarrhea with docetaxel/pertuzumab; proactive antidiarrheals and GI co-management. If later considering neratinib (see below), use prophylaxis (loperamide ± colestipol/budesonide or dose-escalation) per CONTROL trial; weigh carefully given UC history.   Extended anti-HER2 for HR+ disease (optional):   - After finishing the 1-year trastuzumab-based course, consider 1 year of oral neratinib for HR+/HER2+ node-positive patients at higher risk, started within ≤12 months of completing trastuzumab; strongest iDFS benefit is in HR+ subgroup, but diarrhea prevention is essential—use CONTROL-style prophylaxis. Given this patient’s ulcerative colitis, the risk/benefit should be individualized.   *(Note: Adjuvant T-DM1 is indicated only for residual disease after neoadjuvant therapy—not applicable here because surgery preceded systemic therapy.)*  **3) Endocrine therapy**  Indicated (ER/PR 75%+). In a young, node-positive patient, the most effective adjuvant endocrine option is:   - Ovarian function suppression (OFS) + aromatase inhibitor (exemestane) for 5 years, starting after chemotherapy (can be concurrent with ongoing anti-HER2 maintenance). SOFT/TEXT long-term follow-up shows lower recurrence with exemestane+OFS vs tamoxifen±OFS, particularly in higher-risk women. - Duration/extension: At 5 years, reassess; given high risk (age 35, G3, N1), consider extended endocrine therapy to 7–10 years (continue AI if postmenopausal by then, otherwise tamoxifen to 10 years).   Bone protection: If rendered functionally postmenopausal by OFS, discuss adjuvant bisphosphonate (e.g., zoledronic acid 4 mg IV q6 months for 3 years) for reduction in bone recurrence/mortality and to counter AI-related bone loss; get baseline DEXA, calcium/vitamin D, dental check.  **4) Radiation therapy**  Indicated after BCS and node-positive disease.  Fields   - Whole-breast irradiation (WBI) + regional nodal irradiation (RNI) (supraclavicular/level III ± levels I–II and internal mammary chain, tailored to tumor location and axillary management). RNI reduces locoregional and distant recurrences and improves DFS (MA.20; EORTC 22922 15-year).   Dose/fractions (modern options):   - Hypofractionated WBI: 40 Gy in 15 fractions (+ sequential boost 10–16 Gy in 5–8 fx given young age and G3), per ASTRO. - RNI: deliver in the same hypofractionated schedule (e.g., 40 Gy/15 fx) per contemporary practice and data supporting hypofractionated RNI. - (Where appropriate and available) 26 Gy in 5 fractions over 1 week is an accepted alternative for WBI; apply with caution if comprehensive RNI is required (evidence for ultra-hypofractionated RNI remains limited).   Technique/notes: DIBH is more critical for left-sided cases; for right-sided IMN coverage, still optimize heart/lung dose.  **5) Genetics**  Yes — germline testing is indicated.   - 35-year-old at diagnosis meets ASCO/SSO 2024 guidance to test all patients ≤65; testing should be multigene panel centered on BRCA1/2 and PALB2, with inclusion of TP53, CHEK2, ATM based on age/family history. Given invasive lobular carcinoma at a young age, consider CDH1 if there are personal/family features suggestive of hereditary lobular/diffuse gastric cancer (no gastric cancers reported here, but CDH1 may still be considered in young LBC). The family history (breast at 50, pancreatic at 60) further supports testing (BRCA2/PALB2). Refer for genetic counseling.   **Practical sequencing (summary)**   1. No further breast surgery; manage axilla per Z0011/AMAROS as above. 2. Adjuvant TCHP x 6 → continue HP to complete 1 year of anti-HER2 (SC PHESGO acceptable). Echo/MUGA q3 months. Offer GnRH agonist during chemo. 3. Start OFS + exemestane for 5 years (then consider extension). Add adjuvant bisphosphonate if functionally postmenopausal on OFS. 4. Radiation: WBI (40 Gy/15 fx) + boost; add RNI (SCV ± IMN; ± levels I–II by plan). 5. After trastuzumab-based year, consider neratinib (HR+, node-positive) only if UC is quiescent and diarrhea prophylaxis is feasible. 6. Genetic counseling/testing (BRCA1/2, PALB2, TP53, CHEK2/ATM; consider CDH1).   **Small but important caveats for this patient**   - Ulcerative colitis: prioritize diarrhea prevention (diet, loperamide PRN from cycle 1; discuss bile-acid binders if needed). Neratinib may be inadvisable if UC is active; involve gastroenterology early. - Cardiac risk: favor anthracycline-free backbone (TCHP) given excellent efficacy and lower CHF risk vs AC-THP. | This patient is a 35-year-old premenopausal woman with invasive lobular carcinoma, pT2pN1cM0, G3, ER/PR 75%, HER2-positive (IHC 3+), Ki-67 40%, and R0 resection after breast-conserving surgery and sentinel node dissection. She has a significant family history of breast and pancreatic cancer.  **Further surgical treatment:**  No further breast or axillary surgery is indicated, as she has undergone breast-conserving tumorectomy with clear margins (R0) and sentinel node dissection. This is consistent with consensus from the St. Gallen International Expert Panel and the American Society of Clinical Oncology (ASCO) guidelines, which support breast conservation and omission of further axillary surgery in this setting.  **Systemic therapy:**  The American Society of Clinical Oncology recommends that patients with node-positive, HER2-positive early breast cancer receive adjuvant chemotherapy combined with anti-HER2 therapy. The standard regimen is anthracycline- and taxane-based chemotherapy (e.g., doxorubicin/cyclophosphamide followed by paclitaxel) plus trastuzumab (and optionally pertuzumab for higher-risk cases), initiated postoperatively. Trastuzumab is continued for one year. Cardiac function should be monitored due to trastuzumab’s cardiotoxicity risk.  **Endocrine therapy:**  Given her premenopausal status and ER/PR positivity, adjuvant endocrine therapy is indicated. The St. Gallen Panel and ASCO recommend ovarian suppression (e.g., goserelin or leuprolide) plus tamoxifen for higher-risk premenopausal women. Alternatively, ovarian suppression plus aromatase inhibitor may be considered, but tamoxifen remains standard unless there are contraindications. Duration is typically 5 years, with consideration for extension based on risk.  **Radiation therapy:**  Post-lumpectomy, adjuvant whole-breast irradiation is standard. Given node positivity, regional nodal irradiation (including supraclavicular and axillary nodes) is recommended to reduce recurrence risk, as supported by the St. Gallen consensus and ASCO.  **Genetic testing:**  Given her young age, lack of prior births, and family history of breast and pancreatic cancer, genetic testing for hereditary breast cancer syndromes (e.g., BRCA1/2, PALB2) is indicated, as per consensus recommendations for patients with significant family history and early-onset disease.  **Summary:**  • No further surgery  • Adjuvant anthracycline/taxane chemotherapy + trastuzumab (± pertuzumab)  • Ovarian suppression + tamoxifen for 5 years  • Whole-breast and regional nodal irradiation  • Genetic testing for hereditary cancer syndromes  All recommendations are supported by the American Society of Clinical Oncology and the St. Gallen International Expert Panel. |

| **PATIENT CASE 8** |  | |  | |  |  |  |  |
| --- | --- | --- | --- | --- | --- | --- | --- | --- |
|  |  | |  | |  |  |  |  |
| **How difficult or easy was the patient case?** | **very easy** | | **easy** | | **moderate** | **difficult** | **very difficult** | **Score** |
| **Case 8** | **1** | | **2** | | **3** | **4** | **5** |  |
|  |  | |  | |  |  |  |  |
| **The therapy concept is safe.** | **strongly disagree** | | **disagree** | | **neither agree nor disagree** | **agree** | **strongly agree** | **Score** |
| **Therapy Concept 1** | **1** | | **2** | | **3** | **4** | **5** |  |
| **Therapy Concept 2** | **1** | | **2** | | **3** | **4** | **5** |  |
| **Therapy Concept 3** | **1** | | **2** | | **3** | **4** | **5** |  |
|  |  | |  | |  |  |  |  |
| **The therapy concept is in line with the current guidelines.** | **strongly disagree** | | **disagree** | | **neither agree nor disagree** | **agree** | **strongly agree** | **Score** |
| **Therapy Concept 1** | **1** | | **2** | | **3** | **4** | **5** |  |
| **Therapy Concept 2** | **1** | | **2** | | **3** | **4** | **5** |  |
| **Therapy Concept 3** | **1** | | **2** | | **3** | **4** | **5** |  |
|  |  | |  | |  |  |  |  |
| **The therapy concept is medically adequate.** | **strongly disagree** | | **disagree** | | **neither agree nor disagree** | **agree** | **strongly agree** | **Score** |
| **Therapy Concept 1** | **1** | | **2** | | **3** | **4** | **5** |  |
| **Therapy Concept 2** | **1** | | **2** | | **3** | **4** | **5** |  |
| **Therapy Concept 3** | **1** | | **2** | | **3** | **4** | **5** |  |
|  |  | |  | |  |  |  |  |
| **The therapy concept is complete.** | **strongly disagree** | | **disagree** | | **neither agree nor disagree** | **agree** | **strongly agree** | **Score** |
| **Therapy Concept 1** | **1** | | **2** | | **3** | **4** | **5** |  |
| **Therapy Concept 2** | **1** | | **2** | | **3** | **4** | **5** |  |
| **Therapy Concept 3** | **1** | | **2** | | **3** | **4** | **5** |  |
|  |  | |  | |  |  |  |  |
| **The therapy concept is of high quality.** | **strongly disagree** | | **disagree** | | **neither agree nor disagree** | **agree** | **strongly agree** | **Score** |
| **Therapy Concept 1** | **1** | | **2** | | **3** | **4** | **5** |  |
| **Therapy Concept 2** | **1** | | **2** | | **3** | **4** | **5** |  |
| **Therapy Concept 3** | **1** | | **2** | | **3** | **4** | **5** |  |
|  |  | |  | |  |  |  |  |
| **The reasoning behind the treatment decision is logical.** | **strongly disagree** | | **disagree** | | **neither agree nor disagree** | **agree** | **strongly agree** | **Score** |
| **Therapy Concept 1** | **1** | | **2** | | **3** | **4** | **5** |  |
| **Therapy Concept 2** | **1** | | **2** | | **3** | **4** | **5** |  |
| **Therapy Concept 3** | **1** | | **2** | | **3** | **4** | **5** |  |
|  |  | |  | |  |  |  |  |
| **Please rank the therapy concept according to your preference.** | | **Ranking (1^st^, 2^nd^, 3^rd^)** | |  |  |  |  |  |
| **Therapy Concept 1** | |  | |  |  |  |  |  |
| **Therapy Concept 2** | |  | |  |  |  |  |  |
| **Therapy Concept 3** | |  | |  |  |  |  |  |

**Patient 9**

How should the following breast cancer patient be treated based on the most relevant and current international literature?

Patient information:

Age: 58

Menopause status: postmenopausal

ECOG: 1

Previous illness: COPD GOLD B (with inhaled long-acting muscarinic receptor antagonists and inhaled long-acting ß2 sympathomimetics medication)

Previous surgical treatment: None

Birth history: 2 vaginal births at the age of 28 and 30

Oncological family history: Maternal grandmother with endometrial cancer at the age of 75, mother with bile duct carcinoma at the age of 60

The patient underwent the following surgical treatment: Breast-conserving tumorectomy with sentinellymphnodectomy on the left side

Following the surgical treatment, the following TNM-classification, resection margin, histological classification, grading and immunohistology were identified:

TNM: pT1apN0M0

Resection margin: R0, minimal resection margin of 0.05mm

Histological classification: NST

Grading: G2

Multifocality/-centricity: Monofocal and -centric

Estrogen receptor (ER): 5%

Progesterone receptor (PR): 1%

Her2-status: Positive (ISH positive)

Ki-67-proliferation-index: 20%

Please provide a step-by-step treatment recommendation with regard to further surgical treatment, endocrine treatment, systemic treatment and radiation therapy taking the given patient information in consideration.

If endocrine treatment is advisable, please provide a suitable treatment regimen.

If systemic treatment is advisable, please provide a suitable treatment regimen.

If radiation therapy is advisable, please provide a suitable treatment regimen.

Based on the oncological family history, please state whether genetic testing should be performed or not.

| **Patient 9 Treatment Concepts** |  |  |
| --- | --- | --- |
| **Concept 1** | **Concept 2** | **Concept 3** |
| **Step-by-Step Treatment Recommendation**  **1. Further Surgical Treatment**  - No further surgical treatment is required as the patient has undergone breast-conserving surgery with sentinel lymph node biopsy, achieving an R0 resection with clear margins (minimal margin of 0.05 mm). The tumor is monofocal and -centric, and there is no evidence of lymph node involvement (pN0).  **2. Radiation Therapy**  - Indication: Postoperative radiation therapy is recommended for all patients undergoing breast-conserving surgery to reduce the risk of local recurrence.  - Regimen:  - Whole-breast irradiation (WBI) with a total dose of 40–50 Gy delivered in 15–20 fractions over 3–4 weeks.  - A tumor bed boost of 10–16 Gy in 4–8 fractions may be considered, especially given the minimal resection margin of 0.05 mm.  **3. Systemic Treatment**  **3.1 HER2-Targeted Therapy:**  - The patient has HER2-positive breast cancer (ISH-positive). Adjuvant HER2-targeted therapy is strongly recommended.  - Regimen: Trastuzumab for 1 year (administered every 3 weeks or weekly, depending on the formulation). Consider dual HER2 blockade with pertuzumab if high-risk features (e.g., larger tumor size, nodal involvement) were present, but this is not indicated in this case due to the small tumor size (pT1a) and node-negative status.  **3.2 Chemotherapy:**  - Chemotherapy is generally not recommended for HER2-positive, node-negative tumors ≤1 cm (pT1a) unless there are additional high-risk features. In this case, the tumor is small, and the Ki-67 index is moderate (20%), so chemotherapy can be omitted.  **4. Endocrine Therapy:**  - The tumor is ER/PR-low positive (ER 5%, PR 1%), which suggests limited benefit from endocrine therapy. However, given the postmenopausal status, endocrine therapy may still be considered.  - Regimen: Aromatase inhibitor (e.g., letrozole 2.5 mg daily) for 5 years. The decision should be individualized based on the patient’s tolerance and risk-benefit assessment.  **4. Genetic Testing**  - Based on the oncological family history, genetic testing is not routinely indicated. The family history includes endometrial cancer in the maternal grandmother and bile duct carcinoma in the mother, which are not strongly associated with hereditary breast cancer syndromes (e.g., BRCA1/2 mutations). However, if there are additional family members with breast, ovarian, or pancreatic cancer, genetic counseling may be reconsidered.  **Summary of Treatment Plan**  1. Radiation Therapy: Whole-breast irradiation (40–50 Gy in 15–20 fractions) with a tumor bed boost (10–16 Gy in 4–8 fractions).  2. Systemic Therapy:  - HER2-targeted therapy: Trastuzumab for 1 year.  - Endocrine therapy: Aromatase inhibitor (e.g., letrozole) for 5 years, if tolerated.  3. No further surgical treatment is required.  4. No genetic testing is indicated based on the current family history. | Here’s a clear, guideline-anchored plan tailored to the details you provided (58 y, post-menopause; pT1a pN0 M0, NST, G2, HER2-positive [ISH+], ER 5%, PR 1%, Ki-67 20%; breast-conserving surgery with SLNB on the left; R0 with a minimal margin of 0.05 mm; ECOG 1; COPD GOLD-B).  **1) Further surgery**   - No re-excision of the breast: For invasive breast cancer treated with planned whole-breast irradiation, a negative margin defined as “no ink on tumor” is adequate—margin width beyond that does not reduce local recurrence. Your 0.05 mm minimal margin is therefore acceptable; re-excision is not recommended. - No additional axillary surgery: Sentinel nodes are negative → no completion dissection. (Standard practice per NCCN/ESMO.)   **2) Radiation therapy (after lumpectomy)**   - Indication: Yes—whole-breast irradiation (WBI) is standard after breast-conserving surgery. - Preferred fractionation (choose one, based on local practice and logistics):   - 40 Gy in 15 fractions over ~3 weeks (moderately hypofractionated WBI), or   - 26 Gy in 5 fractions given in 1 week (FAST-Forward schedule), which is now widely adopted and non-inferior for tumor control with acceptable late effects. - Tumor-bed boost: Reasonable to give a boost 10–16 Gy in 4–8 fractions (or an equivalent SIB) because of HER2-positive biology and the extremely close margin, even though the margin is technically negative. (Boost reduces local recurrence; strongest benefit is in younger and/or higher-risk features; guidelines allow clinical judgment here.) - Technique/normal-tissue sparing: Left-sided breast → plan with deep-inspiration breath-hold (DIBH) to reduce mean heart dose; also keep lung dose low given COPD (consider prone setup if appropriate). DIBH consistently lowers heart (and often lung) dose. - Sequencing: If you proceed with adjuvant paclitaxel-trastuzumab (see §3), deliver WBI after paclitaxel and concurrently with trastuzumab maintenance; 12–16-week intervals between surgery, RT and systemic therapy are acceptable.   **3) Systemic (anti-HER2 ± chemotherapy) therapy**  You have pT1a (≤5 mm) node-negative HER2-positive disease. For this very small size, international guidance recognizes two reasonable paths; choice hinges on exact tumor size in millimeters, lymphovascular invasion (not reported), comorbidities, and patient preference.  Path A — Omit chemotherapy/anti-HER2 (de-escalation):   - Many guidelines allow no adjuvant chemo-trastuzumab for T1aN0 HER2+ tumors (very-low risk), especially without LVI or grade-3 histology. Recent reviews/meta-analyses show uncertain or small absolute benefit from adding trastuzumab-based chemo in T1aN0. This is an acceptable approach that avoids neuropathy/cardiac risks.   Path B — Offer de-escalated anti-HER2 chemotherapy (maximal risk reduction):   - If the invasive focus is closer to 5 mm, ER is very low (as here), or if any adverse features (e.g., LVI) are present—or if the patient prefers the lowest possible recurrence risk—offer weekly paclitaxel + trastuzumab (the “APT” regimen):   - Paclitaxel 80 mg/m² weekly × 12, with trastuzumab given concurrently (8 mg/kg loading → 6 mg/kg q3wk or 4 mg/kg → 2 mg/kg weekly), then continue trastuzumab alone to complete 1 year total.   - Long-term APT outcomes show excellent 10-year control in small, node-negative HER2+ disease with low serious toxicity. - Cardiac monitoring with trastuzumab: Baseline LVEF (echo or MUGA) and about every 3 months during therapy; manage any decline per cardio-oncology guidance. Continuing trastuzumab can be appropriate with asymptomatic mild LVEF drop while starting cardioprotective meds. - Duration nuance: Standard is 12 months; 6 months may be considered in lower-risk patients or if cardiac toxicity emerges (PERSEPHONE showed non-inferiority overall, though not all meta-analyses agree).   My overall take for *this* patient: With pT1aN0, G2, monofocal disease, no reported LVI, and COPD (neuropathy/pulmonary considerations), either Path A (omit anti-HER2 chemo) or Path B (APT) is guideline-concordant. If the invasive focus is ≤3 mm, I lean to Path A; if it’s 4–5 mm or if the patient strongly prefers the greatest risk reduction, I lean to Path B (APT).  **4) Endocrine therapy (ER 5%, PR 1% → “ER-low”)**   - ER-low (1–10%) tumors behave closer to HR-negative biologically; the benefit from endocrine therapy (ET) is modest/uncertain, but most guidelines still allow or encourage offering ET for any HR-positive invasive cancer, with counseling about limited benefit in ER-low disease. - If you choose ET (reasonable but not mandatory here):   - Aromatase inhibitor (e.g., letrozole 2.5 mg daily) for 5 years is standard in postmenopausal women; tamoxifen 20 mg daily is an alternative if AI is not tolerated/contraindicated.   - Start after paclitaxel (if APT is given); it can run concurrently with trastuzumab and with radiotherapy.   - Manage bone health (baseline DEXA, vitamin D/calcium, exercise) if using an AI. (General standard-of-care measures.) - If you *do not* choose ET: That is acceptable given ER-low status, provided the team documents the discussion about limited expected benefit.   **5) Practical sequencing**   - If APT chosen: Surgery → Paclitaxel+trastuzumab ×12 weeks → WBI (±boost) given concurrently with trastuzumab maintenance → continue trastuzumab to 12 months total → Start AI after paclitaxel (may overlap with RT/trastuzumab). - If APT omitted: Surgery → WBI (±boost) → Consider AI (or omit ET after counseling, as above).   **6) COPD considerations**   - Keep lung dose constraints tight and use DIBH (or prone positioning if helpful) to minimize pneumonitis risk; coordinate with pulmonology on inhaler optimization during RT. (DIBH lowers heart and often lung dose.)   **7) Genetic testing**   - Not routinely indicated based on the information given: unilateral breast cancer at 58 y with no first-degree relatives with breast/ovarian/pancreatic/prostate cancer and no early-onset or triple-negative phenotype. Current NCCN patient guidance reserves testing for specific personal/family histories (e.g., diagnosis at ≤45–50, triple-negative ≤60, ovarian/pancreatic/male breast in the family, multiple close relatives with breast cancer, etc.). You don’t meet those criteria as described. (If additional family history emerges, reassess.)   **Regimen snapshots (for handoff)**   - WBI options   - 40 Gy/15 fx (3 weeks) ± boost 10–16 Gy/4–8 fx, or   - 26 Gy/5 fx (1 week) ± sequential boost (center-specific planning). Use DIBH for left-sided RT. - APT (if chosen)   - Paclitaxel 80 mg/m² weekly ×12 + trastuzumab (8→6 mg/kg q3wk or 4→2 mg/kg weekly), then trastuzumab alone to complete 1 year; LVEF at baseline and ~q3 months. Consider 6 months total trastuzumab if toxicity necessitates shortening. - Endocrine therapy (optional in ER-low)   - Letrozole 2.5 mg PO daily ×5 years (or anastrozole 1 mg / exemestane 25 mg daily); tamoxifen 20 mg daily if AI not tolerated. Start after paclitaxel; can overlap with RT and trastuzumab.   **Why this plan fits today’s literature**   - Margins: “No ink on tumor” is sufficient after BCS with WBI—your 0.05 mm margin does not mandate re-excision. - Small HER2+ tumors: For T1aN0, omission of chemo-trastuzumab is acceptable; when treatment is chosen, APT is the de-escalated standard with excellent 10-year results. - Radiation: Hypofractionation is preferred; FAST-Forward (26/5) is validated; boost reduces local recurrence in selected patients; DIBH cuts heart dose for left-sided RT. - ER-low endocrine therapy: Documented limited benefit; still reasonable to offer with counseling (NCCN considers any HR+ eligible). - Trastuzumab monitoring: Baseline and ~q3-month LVEF checks; mild asymptomatic drops can be managed without automatic discontinuation. | This patient has pT1a pN0 M0, ER-low (5%), PR-low (1%), HER2-positive, G2, monofocal, postmenopausal breast cancer after breast-conserving surgery with clear margins. The most relevant management steps are as follows:  **Further surgical treatment:**  No further surgery is indicated, as margins are negative (R0) and sentinel node is negative. This is consistent with standard practice for early-stage, node-negative disease.  **Radiation therapy:**  Adjuvant whole-breast irradiation is recommended after breast-conserving surgery for invasive breast cancer, regardless of molecular subtype, to reduce local recurrence risk. Hypofractionated regimens (e.g., 40 Gy in 15 fractions) are standard for most patients.  **Systemic therapy:**  For HER2-positive, node-negative, pT1a tumors, the American Society of Clinical Oncology (ASCO) guideline states that adjuvant systemic therapy should be considered, especially if any high-risk features are present (e.g., grade 2, close margin, Ki-67 20%). The standard regimen is trastuzumab (e.g., Hercessi) for 1 year, combined with a taxane (e.g., paclitaxel) for 12 weeks, as per the APT trial, which demonstrated excellent outcomes in this population. Given the patient’s COPD, careful assessment of chemotherapy tolerability is warranted, but the regimen is generally well tolerated.  **Endocrine therapy:**  The tumor is ER/PR-low (≤10%), so the benefit of endocrine therapy is minimal and not routinely recommended for ER/PR <10%. Endocrine therapy (e.g., aromatase inhibitor) may be considered only if the patient strongly prefers maximal risk reduction, but the expected benefit is very limited.  **Genetic testing:**  The family history (endometrial and bile duct cancer) does not meet criteria for routine germline genetic testing for breast cancer predisposition (e.g., BRCA1/2), as per current consensus. Testing may be considered if additional family history of breast/ovarian/pancreatic cancer emerges.  **In summary**  The most evidence-based approach is adjuvant trastuzumab plus paclitaxel, adjuvant whole-breast irradiation, and no further surgery or endocrine therapy. Genetic testing is not indicated based on current family history. |

| **PATIENT CASE 9** |  | |  | |  |  |  |  |
| --- | --- | --- | --- | --- | --- | --- | --- | --- |
|  |  | |  | |  |  |  |  |
| **How difficult or easy was the patient case?** | **very easy** | | **easy** | | **moderate** | **difficult** | **very difficult** | **Score** |
| **Case 9** | **1** | | **2** | | **3** | **4** | **5** |  |
|  |  | |  | |  |  |  |  |
| **The therapy concept is safe.** | **strongly disagree** | | **disagree** | | **neither agree nor disagree** | **agree** | **strongly agree** | **Score** |
| **Therapy Concept 1** | **1** | | **2** | | **3** | **4** | **5** |  |
| **Therapy Concept 2** | **1** | | **2** | | **3** | **4** | **5** |  |
| **Therapy Concept 3** | **1** | | **2** | | **3** | **4** | **5** |  |
|  |  | |  | |  |  |  |  |
| **The therapy concept is in line with the current guidelines.** | **strongly disagree** | | **disagree** | | **neither agree nor disagree** | **agree** | **strongly agree** | **Score** |
| **Therapy Concept 1** | **1** | | **2** | | **3** | **4** | **5** |  |
| **Therapy Concept 2** | **1** | | **2** | | **3** | **4** | **5** |  |
| **Therapy Concept 3** | **1** | | **2** | | **3** | **4** | **5** |  |
|  |  | |  | |  |  |  |  |
| **The therapy concept is medically adequate.** | **strongly disagree** | | **disagree** | | **neither agree nor disagree** | **agree** | **strongly agree** | **Score** |
| **Therapy Concept 1** | **1** | | **2** | | **3** | **4** | **5** |  |
| **Therapy Concept 2** | **1** | | **2** | | **3** | **4** | **5** |  |
| **Therapy Concept 3** | **1** | | **2** | | **3** | **4** | **5** |  |
|  |  | |  | |  |  |  |  |
| **The therapy concept is complete.** | **strongly disagree** | | **disagree** | | **neither agree nor disagree** | **agree** | **strongly agree** | **Score** |
| **Therapy Concept 1** | **1** | | **2** | | **3** | **4** | **5** |  |
| **Therapy Concept 2** | **1** | | **2** | | **3** | **4** | **5** |  |
| **Therapy Concept 3** | **1** | | **2** | | **3** | **4** | **5** |  |
|  |  | |  | |  |  |  |  |
| **The therapy concept is of high quality.** | **strongly disagree** | | **disagree** | | **neither agree nor disagree** | **agree** | **strongly agree** | **Score** |
| **Therapy Concept 1** | **1** | | **2** | | **3** | **4** | **5** |  |
| **Therapy Concept 2** | **1** | | **2** | | **3** | **4** | **5** |  |
| **Therapy Concept 3** | **1** | | **2** | | **3** | **4** | **5** |  |
|  |  | |  | |  |  |  |  |
| **The reasoning behind the treatment decision is logical.** | **strongly disagree** | | **disagree** | | **neither agree nor disagree** | **agree** | **strongly agree** | **Score** |
| **Therapy Concept 1** | **1** | | **2** | | **3** | **4** | **5** |  |
| **Therapy Concept 2** | **1** | | **2** | | **3** | **4** | **5** |  |
| **Therapy Concept 3** | **1** | | **2** | | **3** | **4** | **5** |  |
|  |  | |  | |  |  |  |  |
| **Please rank the therapy concept according to your preference.** | | **Ranking (1^st^, 2^nd^, 3^rd^)** | |  |  |  |  |  |
| **Therapy Concept 1** | |  | |  |  |  |  |  |
| **Therapy Concept 2** | |  | |  |  |  |  |  |
| **Therapy Concept 3** | |  | |  |  |  |  |  |

**Patient 10**

How should the following breast cancer patient be treated based on the most relevant and current international literature?

Patient information:

Age: 65

Menopause status: postmenopausal

ECOG: 2

Previous illness: Atrial fibrillation (with direct oral anticoagulant and beta-blocker medication), pulmonary artery embolism at the age of 65 following immobilization during right-sided total hip arthroplasty

Previous surgical treatment: Right-sided total hip arthroplasty at the age of 65

Birth history: 2 vaginal births at the age of 23 and 30 and 1 cesarean at the age of 35

Oncological family history: Sister with childhood acute lymphoblastic leukemia, father with gastric carcinoma at the age of 50

The patient underwent the following surgical treatment: Mastectomy with sentinellymphnodectomy on the right side

Following the surgical treatment, the following TNM-classification, resection margin, histological classification, grading and immunohistology were identified:

TNM: pT3pN1aM0

Resection margin: R0, minimal resection margin of 10 mm

Histological classification: NST

Grading: G2

Multifocality/-centricity: Monofocal and -centric

Estrogen receptor (ER): 0%

Progesterone receptor (PR): 0%

Her2-status: Positive (ICH 3+)

Ki-67-proliferation-index: 35%

Please provide a step-by-step treatment recommendation with regard to further surgical treatment, endocrine treatment, systemic treatment and radiation therapy taking the given patient information in consideration.

If endocrine treatment is advisable, please provide a suitable treatment regimen.

If systemic treatment is advisable, please provide a suitable treatment regimen.

If radiation therapy is advisable, please provide a suitable treatment regimen.

Based on the oncological family history, please state whether genetic testing should be performed or not.

| **Patient 10 Treatment Concepts** |  |  |
| --- | --- | --- |
| **Concept 1** | **Concept 2** | **Concept 3** |
| **Treatment Recommendation for the Given Patient**  **1. Further Surgical Treatment**  - No further surgical treatment is required as the patient has undergone a mastectomy with sentinel lymph node dissection, achieving an R0 resection with a clear margin of 10 mm. This is considered adequate for local control in the context of pT3pN1aM0 disease.  **2. Endocrine Treatment**  - Endocrine therapy is not indicated as the tumor is estrogen receptor (ER)-negative and progesterone receptor (PR)-negative.  **3. Systemic Treatment**  Given the patient's HER2-positive, high-risk profile (pT3, N1, Ki-67 of 35%), systemic therapy is strongly recommended. The following regimen is advised:  - Neoadjuvant/Adjuvant Chemotherapy with HER2-Targeted Therapy:  - Chemotherapy Backbone:  - Anthracycline-based regimen (e.g., doxorubicin and cyclophosphamide) for 4 cycles, followed by  - Taxane-based regimen (e.g., paclitaxel or docetaxel) for 4 cycles.  - HER2-Targeted Therapy:  - Trastuzumab (HER2 monoclonal antibody) should be initiated concurrently with the taxane phase and continued for a total of 1 year.  - Consider adding pertuzumab (dual HER2 blockade) during the chemotherapy phase, especially given the high-risk features (pT3, N1).  - Cardiac Monitoring:  - Given the patient’s history of atrial fibrillation and pulmonary embolism, careful cardiac monitoring (e.g., echocardiography or MUGA scans) is essential during HER2-targeted therapy due to the risk of cardiotoxicity.  -Anticoagulation Consideration:  - The patient’s direct oral anticoagulant (DOAC) therapy should be carefully managed during chemotherapy to minimize bleeding risks, particularly if thrombocytopenia occurs.  **4. Radiation Therapy**  - Radiation therapy is recommended due to the following factors:  - Tumor size (pT3).  - Nodal involvement (pN1a).  - Radiation Plan:  - Chest Wall Radiation: Post-mastectomy radiation therapy (PMRT) to the chest wall.  - Regional Nodal Radiation: Include the supraclavicular, infraclavicular, and axillary lymph node basins.  - Dose and Fractionation:  - Standard dose: 50 Gy in 25 fractions over 5 weeks.  - Hypofractionated regimens (e.g., 40 Gy in 15 fractions) may be considered based on institutional protocols and patient preference.  **5. Genetic Testing**  - Genetic testing is not routinely indicated based on the provided family history. While the patient’s sister had childhood acute lymphoblastic leukemia and the father had gastric carcinoma, these cancers are not strongly associated with hereditary breast cancer syndromes (e.g., BRCA1/2 mutations).  - However, if there is additional family history of breast, ovarian, or other BRCA-associated cancers, genetic counseling and testing may be reconsidered.  **Summary of Treatment Plan**  1. No further surgical treatment is required.  2. Endocrine therapy is not indicated due to ER/PR negativity.  3. Systemic therapy:  - Anthracycline-taxane chemotherapy with trastuzumab ± pertuzumab.  - Continue trastuzumab for 1 year with cardiac monitoring.  4. Radiation therapy:  - Post-mastectomy chest wall and regional nodal radiation.  5. Genetic testing is not indicated based on the current family history. | Here’s a concise, guideline-based plan for this 65-year-old, postmenopausal, ECOG 2 patient with pT3 pN1a M0, ER/PR-negative, HER2-positive (IHC 3+) invasive breast cancer (NST, G2) after right mastectomy + sentinel node biopsy.  **1) Any further surgery?**  No re-excision is needed (R0, 10 mm margins).  Axilla: because only a sentinel node procedure was done and 1–3 nodes are positive (pN1a), completion ALND can be omitted if you give regional nodal irradiation (RNI) as part of post-mastectomy RT. This approach is now endorsed by ASCO (2025 update) and supported by AMAROS long-term data (axillary RT ≈ ALND for control, with less lymphedema).  **2) Staging & timing checks (do now)**  Systemic staging (if not already done): CT chest/abdomen + bone scan or FDG-PET/CT—recommended for stage IIB–III disease.  Start adjuvant systemic therapy promptly (ideally within ~4–6 weeks of surgery).  Baseline echo (LVEF) before anti-HER2 therapy; repeat about every 3 months during therapy (ESC cardio-oncology guidance / drug label).  **3) Endocrine therapy**  Not indicated. Tumor is ER 0% / PR 0%. (Guidelines restrict endocrine therapy to HR-positive disease.)  **4) Adjuvant systemic therapy (HER2-targeted chemo)**  Given node-positive, T3 disease and ECOG 2 with atrial fibrillation on a DOAC, favor an anthracycline-free regimen to limit cardiotoxicity:  Preferred (if reasonably fit):  TCHP × 6 cycles q3 weeks  Docetaxel 75 mg/m² + Carboplatin AUC 6 (Day 1)  Trastuzumab (8 mg/kg load → 6 mg/kg q3w) + Pertuzumab (840 mg load → 420 mg q3w)  Then continue trastuzumab ± pertuzumab to complete 1 year of anti-HER2 therapy. The APHINITY trial shows sustained benefit of adding pertuzumab in node-positive early HER2+ disease, including overall-survival benefit at 10 years. Anthracycline-free TCH had efficacy similar to AC-TH with less cardiotoxicity (BCIRG-006).  If pertuzumab is not tolerated or frailty dominates:  TCH (docetaxel/carboplatin/trastuzumab) × 6, then finish trastuzumab to 1 year. Still avoids anthracyclines and has long-standing evidence.  What’s not indicated here:  Adjuvant T-DM1 (KATHERINE) is for residual disease after neoadjuvant therapy—doesn’t apply after primary surgery.  Extended adjuvant neratinib benefits HR-positive disease only—not for HR-negative tumors.  **Supportive care & safety notes**  G-CSF prophylaxis: consider primary prophylaxis with TCH(P) if your institution regards the regimen/patient as ≥ intermediate risk for febrile neutropenia (age ~65, ECOG 2, comorbidities tilt toward use).  Cardiac monitoring: baseline and ~q3 months during anti-HER2; closer follow-up if LVEF falls or symptoms develop.  Anticoagulation: continue DOAC for AF/PE; coordinate around port placement and monitor counts for bleeding risk during docetaxel/carboplatin.  **5) Post-mastectomy radiation therapy (PMRT)**  Indication: Strong for T3 and node-positive disease after mastectomy. Plan chest wall + regional nodes.  Volumes:  Chest wall;  RNI: supraclavicular + axillary levels II–III (include level I if no ALND) and consider internal mammary nodes (IMN)—commonly included for T3/N+ to improve disease control, balancing heart/lung doses.  Dose/fractionation (choose per center protocol & anatomy):  Conventional: 50 Gy in 25 fractions (5 weeks) to chest wall and RNI; or  Moderate hypofractionation: ~40–42.5 Gy in 15–16 fractions (3 weeks) to chest wall/RNI (endorsed in contemporary guidance and used in trials), adjusting nodal prescription per protocol. No boost typically needed with 10 mm negative margins.  Sequencing & concurrency:  Give chemo first, then PMRT. Trastuzumab (± pertuzumab) may continue during RT—data support good tolerability  **6) Bone-modifying therapy (discuss)**  For postmenopausal women receiving adjuvant systemic therapy, adjuvant bisphosphonates (e.g., zoledronic acid 4 mg IV q6 months for 3 years) lower bone recurrence and modestly improve survival; discuss risks (ONJ, renal) and ensure a dental check beforehand.  **7) Genetic testing—should it be done?**  Yes. The 2024 ASCO–SSO guideline recommends offering BRCA1/2 testing to all newly diagnosed patients ≤ 65 years (and select > 65). At age 65, she qualifies regardless of family history. A multigene panel that at least includes BRCA1/2 (± PALB2 and others per local policy) is reasonable after counselling.    **One practical pathway (pulling it together)**  Confirm no distant disease on CT/bone scan or PET/CT; baseline labs and echo.  Adjuvant TCHP × 6 → continue trastuzumab ± pertuzumab to complete 1 year (LVEF checks q3 months). Use G-CSF if indicated.  PMRT to chest wall + RNI (include axilla given no ALND; consider IMN). Dose 50 Gy/25 or ~40 Gy/15 per local standard; give concurrently with ongoing anti-HER2.  Discuss adjuvant zoledronic acid q6 months × 3 years.  Offer germline testing (BRCA1/2±panel).  Notes tailored to this case: anthracyclines are avoidable (cardio-safer TCH/TCHP has comparable efficacy); endocrine therapy is not indicated (ER/PR 0%); T-DM1/neratinib do not apply here. | This patient has postmenopausal, HER2-positive, hormone receptor-negative (ER 0%, PR 0%) breast cancer, stage pT3pN1aM0, grade 2, with a high Ki-67 (35%), and a history of atrial fibrillation and prior pulmonary embolism. The following evidence-based recommendations are appropriate:  **Further surgical treatment:**  No further breast or axillary surgery is indicated, as the patient has undergone mastectomy with sentinel lymph node dissection and achieved R0 resection with adequate margins. This is consistent with current standards for local control in node-positive disease.  **Endocrine treatment:**  Endocrine therapy is not indicated because the tumor is negative for both estrogen and progesterone receptors. The American Society of Clinical Oncology (ASCO) and the medical literature agree that endocrine therapy provides no benefit in ER/PR-negative disease.  **Systemic treatment:**  Adjuvant systemic therapy is indicated. For HER2-positive, node-positive, high-risk breast cancer, the standard is a combination of chemotherapy and anti-HER2 targeted therapy. The recommended regimen is a taxane-based chemotherapy (e.g., docetaxel or paclitaxel) plus trastuzumab, with or without pertuzumab, for one year. Anthracyclines may be considered, but given the patient’s age, performance status (ECOG 2), and comorbidities (notably atrial fibrillation and prior embolism), a non-anthracycline regimen (e.g., docetaxel, carboplatin, trastuzumab, and pertuzumab—TCHP) may be preferred to minimize cardiotoxicity and thrombotic risk. Trastuzumab should be continued for 1 year, with cardiac monitoring.  **Radiation therapy:**  Adjuvant radiation therapy is recommended for patients with pT3 and/or node-positive disease after mastectomy, as it reduces locoregional recurrence and improves survival. The standard is chest wall irradiation, with possible inclusion of regional lymphatics (axillary, supraclavicular, and internal mammary nodes) depending on risk assessment.  **Genetic testing:**  Genetic testing should be considered due to the family history of early-onset gastric carcinoma in the father and childhood leukemia in the sister. While these are not classic hereditary breast cancer syndromes, the presence of multiple cancers in first-degree relatives warrants assessment for hereditary cancer predisposition, including BRCA1/2 and other relevant genes.  All recommendations are supported by the American Society of Clinical Oncology and current international literature. |

| **PATIENT CASE 10** |  | |  | |  |  |  |  |
| --- | --- | --- | --- | --- | --- | --- | --- | --- |
|  |  | |  | |  |  |  |  |
| **How difficult or easy was the patient case?** | **very easy** | | **easy** | | **moderate** | **difficult** | **very difficult** | **Score** |
| **Case 10** | **1** | | **2** | | **3** | **4** | **5** |  |
|  |  | |  | |  |  |  |  |
| **The therapy concept is safe.** | **strongly disagree** | | **disagree** | | **neither agree nor disagree** | **agree** | **strongly agree** | **Score** |
| **Therapy Concept 1** | **1** | | **2** | | **3** | **4** | **5** |  |
| **Therapy Concept 2** | **1** | | **2** | | **3** | **4** | **5** |  |
| **Therapy Concept 3** | **1** | | **2** | | **3** | **4** | **5** |  |
|  |  | |  | |  |  |  |  |
| **The therapy concept is in line with the current guidelines.** | **strongly disagree** | | **disagree** | | **neither agree nor disagree** | **agree** | **strongly agree** | **Score** |
| **Therapy Concept 1** | **1** | | **2** | | **3** | **4** | **5** |  |
| **Therapy Concept 2** | **1** | | **2** | | **3** | **4** | **5** |  |
| **Therapy Concept 3** | **1** | | **2** | | **3** | **4** | **5** |  |
|  |  | |  | |  |  |  |  |
| **The therapy concept is medically adequate.** | **strongly disagree** | | **disagree** | | **neither agree nor disagree** | **agree** | **strongly agree** | **Score** |
| **Therapy Concept 1** | **1** | | **2** | | **3** | **4** | **5** |  |
| **Therapy Concept 2** | **1** | | **2** | | **3** | **4** | **5** |  |
| **Therapy Concept 3** | **1** | | **2** | | **3** | **4** | **5** |  |
|  |  | |  | |  |  |  |  |
| **The therapy concept is complete.** | **strongly disagree** | | **disagree** | | **neither agree nor disagree** | **agree** | **strongly agree** | **Score** |
| **Therapy Concept 1** | **1** | | **2** | | **3** | **4** | **5** |  |
| **Therapy Concept 2** | **1** | | **2** | | **3** | **4** | **5** |  |
| **Therapy Concept 3** | **1** | | **2** | | **3** | **4** | **5** |  |
|  |  | |  | |  |  |  |  |
| **The therapy concept is of high quality.** | **strongly disagree** | | **disagree** | | **neither agree nor disagree** | **agree** | **strongly agree** | **Score** |
| **Therapy Concept 1** | **1** | | **2** | | **3** | **4** | **5** |  |
| **Therapy Concept 2** | **1** | | **2** | | **3** | **4** | **5** |  |
| **Therapy Concept 3** | **1** | | **2** | | **3** | **4** | **5** |  |
|  |  | |  | |  |  |  |  |
| **The reasoning behind the treatment decision is logical.** | **strongly disagree** | | **disagree** | | **neither agree nor disagree** | **agree** | **strongly agree** | **Score** |
| **Therapy Concept 1** | **1** | | **2** | | **3** | **4** | **5** |  |
| **Therapy Concept 2** | **1** | | **2** | | **3** | **4** | **5** |  |
| **Therapy Concept 3** | **1** | | **2** | | **3** | **4** | **5** |  |
|  |  | |  | |  |  |  |  |
| **Please rank the therapy concept according to your preference.** | | **Ranking (1^st^, 2^nd^, 3^rd^)** | |  |  |  |  |  |
| **Therapy Concept 1** | |  | |  |  |  |  |  |
| **Therapy Concept 2** | |  | |  |  |  |  |  |
| **Therapy Concept 3** | |  | |  |  |  |  |  |

**Patient 11**

How should the following breast cancer patient be treated based on the most relevant and current international literature?

Patient information:

Age: 32

Menopause status: premenopausal

ECOG: 0

Previous illness: Insulin-dependent gestational diabetes during the first pregnancy at the age of 20, postpartum depression at the age of 20

Previous surgical treatment: None

Birth history: 1 vaginal birth at the age of 20

Oncological family history: Father with bronchial carcinoma at the age of 60, sister with osteosarcoma at the age of 18

The patient underwent the following surgical treatment: Breast-conserving tumorectomy with sentinellymphnodectomy on the left side

Following the surgical treatment, the following TNM-classification, resection margin, histological classification, grading and immunohistology were identified:

TNM: pT2pN0M0

Resection margin: R0, minimal resection margin of 4 mm

Histological classification: NST

Grading: G2

Multifocality/-centricity: Monofocal and -centric

Estrogen receptor (ER): 0%

Progesterone receptor (PR): 0%

Her2-status: Positive (ICH 3+)

Ki-67-proliferation-index: 65%

Please provide a step-by-step treatment recommendation with regard to further surgical treatment, endocrine treatment, systemic treatment and radiation therapy taking the given patient information in consideration.

If endocrine treatment is advisable, please provide a suitable treatment regimen.

If systemic treatment is advisable, please provide a suitable treatment regimen.

If radiation therapy is advisable, please provide a suitable treatment regimen.

Based on the oncological family history, please state whether genetic testing should be performed or not.

| **Patient 11 Treatment Concepts** |  |  |
| --- | --- | --- |
| **Concept 1** | **Concept 2** | **Concept 3** |
| **Treatment Recommendations for the Patient:**  **1. Surgical Treatment:**  - No further surgical treatment is required as the patient has undergone a breast-conserving tumorectomy with sentinel lymph node dissection, achieving clear resection margins (R0) with a minimal margin of 4 mm. This is considered adequate for local control.  **2. Systemic Treatment:**  **2.1. Chemotherapy:**  - Given the patient's high-risk features, including HER2-positive status (IHC 3+), triple-negative hormone receptor status (ER 0%, PR 0%), high Ki-67 proliferation index (65%), and pT2 tumor size, adjuvant systemic chemotherapy is strongly recommended.  - Recommended regimen:  - Anthracycline and taxane-based chemotherapy:  - Doxorubicin (60 mg/m²) and Cyclophosphamide (600 mg/m²) every 2 weeks for 4 cycles (dose-dense AC) followed by Paclitaxel (80 mg/m²) weekly for 12 weeks.  **2.2 Anti-HER2 therapy:**  - Trastuzumab (8 mg/kg loading dose, then 6 mg/kg every 3 weeks) combined with Pertuzumab (840 mg loading dose, then 420 mg every 3 weeks) during the taxane phase, followed by maintenance Trastuzumab for a total of 1 year.  **3. Endocrine Treatment:**  - Endocrine therapy is not indicated as the tumor is hormone receptor-negative (ER 0%, PR 0%).  **4. Radiation Therapy:**  - Postoperative radiation therapy is recommended due to the breast-conserving surgery and the tumor size (pT2).  - Recommended regimen:  - Whole-breast irradiation (WBI): 50 Gy in 25 fractions over 5 weeks.  - Tumor bed boost: An additional 10–16 Gy in 4–8 fractions to the tumor bed, considering the patient’s young age and high-risk features.  **5. Genetic Testing:**  - Genetic testing is recommended based on the patient’s young age (32 years), triple-negative breast cancer (TNBC) status, and significant family history of cancer (sister with osteosarcoma at 18 years, father with bronchial carcinoma at 60 years).  - Recommended testing:  - BRCA1/BRCA2 mutation analysis.  - Consideration of a broader panel for hereditary cancer syndromes, including TP53 (Li-Fraumeni syndrome) and other relevant genes.  **Summary of Treatment Plan:**  1. Surgical Treatment: No further surgery required.  2. Systemic Treatment: Anthracycline and taxane-based chemotherapy with concurrent anti-HER2 therapy (Trastuzumab and Pertuzumab).  3. Endocrine Treatment: Not indicated.  4. Radiation Therapy: Whole-breast irradiation with a tumor bed boost.  5. Genetic Testing: Recommended for BRCA1/BRCA2 and other hereditary cancer syndromes. | Here’s an evidence-based, step-by-step plan for this 32-year-old, premenopausal patient with pT2 pN0 M0, ER/PR-negative, HER2-positive (IHC 3+), G2, Ki-67 65% breast cancer after breast-conserving surgery (R0, 4 mm margin). I’m drawing on the most current international guidance and major trials.  **1) Further surgery**   - No re-excision indicated. For invasive cancer treated with breast-conserving surgery, “no tumor on ink” is an adequate margin; wider margins do not reduce local recurrence. Your 4 mm negative margin meets this standard. - Axilla: Sentinel nodes are negative → no further axillary surgery.   Important exception to consider now: because of the family history (sister with osteosarcoma at 18) and very young age, urgent germline testing (including TP53) is warranted before committing to radiation. A pathogenic TP53 (Li-Fraumeni) result would favor completion mastectomy to avoid breast irradiation (elevated radiation-induced malignancy risk in LFS). You can proceed with systemic therapy while testing is arranged and finalize breast/radiation decisions once results return.  **2) Endocrine therapy**   - Not indicated. Tumor is ER 0% and PR 0%. Extended options like neratinib are only for HR-positive disease—not applicable here.   **3) Adjuvant systemic therapy (chemotherapy + anti-HER2)**  Goal: Reduce distant and local recurrence risk for a pT2N0, HR-negative, HER2-positive tumor.  Recommended regimen (preferred, anthracycline-free):   - TCH → H *Docetaxel + Carboplatin + Trastuzumab (q3 weeks × 6 cycles),* then Trastuzumab alone to complete 12 months total anti-HER2 therapy. Typical dosing: docetaxel 75 mg/m² + carboplatin AUC 6 q3 weeks ×6; trastuzumab 8 mg/kg load then 6 mg/kg q3 weeks during chemo and to complete 1 year. This matches pivotal data showing similar efficacy but less cardiotoxicity vs anthracycline-containing AC-TH.   Acceptable alternative (if TCH contraindicated):   - ddAC-TH (dose-dense doxorubicin/cyclophosphamide ×4 → paclitaxel + trastuzumab), acknowledging higher cardiotoxicity risk vs TCH without clear efficacy advantage.   What not to add in this case:   - Pertuzumab: Adjuvant benefit is confined to node-positive cohorts; no benefit in node-negative patients on long-term APHINITY follow-up. Thus, do not add pertuzumab here. - T-DM1: Reserved for residual invasive disease after neoadjuvant therapy (KATHERINE). Not applicable after primary surgery with no prior neoadjuvant therapy. - De-escalated TH (weekly paclitaxel + trastuzumab): Excellent for small (T1), node-negative tumors (APT trial) but insufficient for T2 high-risk disease like this case.   Timing & monitoring:   - Aim to start adjuvant chemotherapy promptly after surgery (delays >60 days are associated with worse outcomes in HER2-positive disease). - Cardiac monitoring: Baseline LVEF (echo/MUGA), then about every 3 months during trastuzumab per label/ESC guidance (some centers individualize to every 6 months for low-risk, non-anthracycline regimens, but 3-monthly remains the conservative standard).   Fertility & ovarian function:   - At 32 and premenopausal, discuss embryo/oocyte cryopreservation up front and consider temporary GnRH agonist (e.g., goserelin) during chemotherapy to reduce premature ovarian insufficiency and increase post-treatment pregnancy rates—especially well supported in HR-negative disease.   **4) Radiation therapy (after chemotherapy; trastuzumab may continue during RT)**  Indication: Post-lumpectomy whole-breast irradiation (WBI) is standard for local control in invasive cancer with breast conservation.  Recommended schedule:   - Hypofractionated WBI (preferred): • 40 Gy in 15 fractions (3 weeks), *or* 42.56 Gy in 16 fractions (3.2 weeks). These are ASTRO-endorsed as preferred options, regardless of age or chemo receipt. - Tumor bed boost: Yes. Given age 32 and HR-negative/HER2-positive biology, add a boost 10–16 Gy in 4–8 fractions to the lumpectomy cavity. - Regional nodes: Not indicated for pN0 without additional high-risk features. (Reassess only if new adverse pathology emerges.) - Concurrent trastuzumab: Acceptable to continue trastuzumab during WBI; routine practice with acceptable safety.   If TP53 (Li-Fraumeni) positive: favor mastectomy to avoid breast RT; if breast conservation is maintained despite LFS, RT should be avoided or minimized due to secondary malignancy risk—this would require a dedicated genetics/radiation risk discussion.  **5) Genetic testing:**  Yes - urgently.   - ASCO/SSO 2024: offer BRCA1/2 testing to all patients ≤65 at breast cancer diagnosis (broader than past criteria). At 32, she qualifies. - Given early onset (32) and a first-degree relative with osteosarcoma at 18, test TP53 (Li-Fraumeni) and other high-penetrance genes (e.g., PALB2, CHEK2, ATM, PTEN) via a multigene panel per NCCN genetics guidance. A TP53 result could change local therapy (mastectomy to avoid RT) and long-term surveillance.   **Practical, one-page regimen summary (to copy into the chart)**  Systemic (start first):   - TCH × 6 cycles q3 weeks (docetaxel 75 mg/m² + carboplatin AUC 6 + trastuzumab 8→6 mg/kg), then trastuzumab q3 weeks to complete 12 months total. Echos at baseline and about q3 months. Consider goserelin during chemo for ovarian protection. No pertuzumab. No endocrine therapy.   Radiation (after chemo; overlap with trastuzumab OK):   - WBI 40 Gy/15 fx or 42.56 Gy/16 fx, plus boost 10–16 Gy/4–8 fx to the tumor bed. No regional nodal irradiation for pN0. (Revisit if TP53 positive → consider mastectomy and omit RT.)   Surgery:   - No further breast/axillary surgery required given R0 and pN0—unless TP53 positive and electing mastectomy to avoid RT.   Genetics (do now):   - Multigene germline testing including BRCA1/2, TP53, PALB2, CHEK2, ATM, PTEN with pre-/post-test counseling. TP53 result may alter local therapy and surveillance plan.   **Notes & caveats**   - If neoadjuvant therapy had been given and residual disease found, adjuvant T-DM1 for 14 cycles would be standard; not relevant here. - Keep trastuzumab on schedule; discuss cardiotoxicity risks and risk-factor control; involve cardio-oncology for any LVEF drop. | This patient has early-stage, HER2-positive, hormone receptor-negative (ER-/PR-), grade 2, pT2N0M0 breast cancer, status post breast-conserving surgery with clear margins. She is premenopausal, with excellent performance status and no significant comorbiditiespertuzumab to trastuzumab-based.  **Further surgical treatment:**  No additional breast or axillary surgery is indicated, as margins are negative (R0, 4 mm) and sentinel nodes are negative.  **Endocrine treatment:**  Endocrine therapy is not indicated because the tumor is ER- and PR- negative.  **Systemic treatment:**  The standard of care is adjuvant chemotherapy combined with anti-HER2 therapy. The American Society of Clinical Oncology (ASCO) recommends a regimen of anthracycline- and/or taxane-based chemotherapy plus trastuzumab for HER2-positive, node-negative tumors >1 cm (T2).  Typical regimens:  • Docetaxel + carboplatin + trastuzumab (TCH)  • Doxorubicin + cyclophosphamide followed by paclitaxel + trastuzumab (AC→TH)  Trastuzumab is given for a total of 1 year.  • For high-risk features (e.g., high Ki-67, young age), consider adding pertuzumab to trastuzumab (dual HER2 blockade) during chemotherapy, as supported by pivotal trials.  • If residual disease is present after neoadjuvant therapy, adjuvant T-DM1 is indicated, but in this case, surgery was first and margins are clear.  **Radiation therapy:**  Adjuvant whole-breast radiotherapy is indicated after breast-conserving surgery, per standard practice.  • Typical regimen: 40–50 Gy in 15–25 fractions over 3–5 weeks, with or without a tumor bed boost.  **Genetic testing:**  Given the strong family history of early-onset cancers (osteosarcoma in sister, bronchial carcinoma in father) and young age at diagnosis, genetic counseling and testing for hereditary cancer syndromes (including BRCA1/2) is recommended. This may impact future management and family risk assessment.  **In summary:**  The patient should receive adjuvant chemotherapy plus trastuzumab (± pertuzumab), followed by adjuvant radiotherapy, and undergo genetic testing due to her family history. Endocrine therapy is not indicated. |

| **PATIENT CASE 11** |  | |  | |  |  |  |  |
| --- | --- | --- | --- | --- | --- | --- | --- | --- |
|  |  | |  | |  |  |  |  |
| **How difficult or easy was the patient case?** | **very easy** | | **easy** | | **moderate** | **difficult** | **very difficult** | **Score** |
| **Case 11** | **1** | | **2** | | **3** | **4** | **5** |  |
|  |  | |  | |  |  |  |  |
| **The therapy concept is safe.** | **strongly disagree** | | **disagree** | | **neither agree nor disagree** | **agree** | **strongly agree** | **Score** |
| **Therapy Concept 1** | **1** | | **2** | | **3** | **4** | **5** |  |
| **Therapy Concept 2** | **1** | | **2** | | **3** | **4** | **5** |  |
| **Therapy Concept 3** | **1** | | **2** | | **3** | **4** | **5** |  |
|  |  | |  | |  |  |  |  |
| **The therapy concept is in line with the current guidelines.** | **strongly disagree** | | **disagree** | | **neither agree nor disagree** | **agree** | **strongly agree** | **Score** |
| **Therapy Concept 1** | **1** | | **2** | | **3** | **4** | **5** |  |
| **Therapy Concept 2** | **1** | | **2** | | **3** | **4** | **5** |  |
| **Therapy Concept 3** | **1** | | **2** | | **3** | **4** | **5** |  |
|  |  | |  | |  |  |  |  |
| **The therapy concept is medically adequate.** | **strongly disagree** | | **disagree** | | **neither agree nor disagree** | **agree** | **strongly agree** | **Score** |
| **Therapy Concept 1** | **1** | | **2** | | **3** | **4** | **5** |  |
| **Therapy Concept 2** | **1** | | **2** | | **3** | **4** | **5** |  |
| **Therapy Concept 3** | **1** | | **2** | | **3** | **4** | **5** |  |
|  |  | |  | |  |  |  |  |
| **The therapy concept is complete.** | **strongly disagree** | | **disagree** | | **neither agree nor disagree** | **agree** | **strongly agree** | **Score** |
| **Therapy Concept 1** | **1** | | **2** | | **3** | **4** | **5** |  |
| **Therapy Concept 2** | **1** | | **2** | | **3** | **4** | **5** |  |
| **Therapy Concept 3** | **1** | | **2** | | **3** | **4** | **5** |  |
|  |  | |  | |  |  |  |  |
| **The therapy concept is of high quality.** | **strongly disagree** | | **disagree** | | **neither agree nor disagree** | **agree** | **strongly agree** | **Score** |
| **Therapy Concept 1** | **1** | | **2** | | **3** | **4** | **5** |  |
| **Therapy Concept 2** | **1** | | **2** | | **3** | **4** | **5** |  |
| **Therapy Concept 3** | **1** | | **2** | | **3** | **4** | **5** |  |
|  |  | |  | |  |  |  |  |
| **The reasoning behind the treatment decision is logical.** | **strongly disagree** | | **disagree** | | **neither agree nor disagree** | **agree** | **strongly agree** | **Score** |
| **Therapy Concept 1** | **1** | | **2** | | **3** | **4** | **5** |  |
| **Therapy Concept 2** | **1** | | **2** | | **3** | **4** | **5** |  |
| **Therapy Concept 3** | **1** | | **2** | | **3** | **4** | **5** |  |
|  |  | |  | |  |  |  |  |
| **Please rank the therapy concept according to your preference.** | | **Ranking (1^st^, 2^nd^, 3^rd^)** | |  |  |  |  |  |
| **Therapy Concept 1** | |  | |  |  |  |  |  |
| **Therapy Concept 2** | |  | |  |  |  |  |  |
| **Therapy Concept 3** | |  | |  |  |  |  |  |

**Patient 12**

How should the following breast cancer patient be treated based on the most relevant and current international literature?

Patient information:

Age: 42

Menopause status: premenopausal

ECOG: 0

Previous illness: Pulmonary artery embolism after pelvic vein thrombosis at the age of 26, antiphospholipid syndrome with anti-cardiolipin antibodies (with permanent oral anticoagulation with phenprocoumon)

Previous surgical treatment: None

Birth history: 6 early abortions between the age of 20 and 26

Oncological family history: Father with colon cancer at the age of 45, paternal grandmother with endometrial cancer at the age of 65, paternal uncle with urothelial carcinoma of the renal pelvis at the age of 55

The patient underwent the following surgical treatment: Breast-conserving tumorectomy with sentinellymphnodectomy on the right side

Following the surgical treatment, the following TNM-classification, resection margin, histological classification, grading and immunohistology were identified:

TNM: pT2pN1M0

Resection margin: R0, minimal resection margin of 2 mm

Histological classification: NST

Grading: G3

Multifocality/-centricity: Monofocal and -centric

Estrogen receptor (ER): 0%

Progesterone receptor (PR): 5%

Her2-status: Positive (ISH positive)

Ki-67-proliferation-index: 80%

Please provide a step-by-step treatment recommendation with regard to further surgical treatment, endocrine treatment, systemic treatment and radiation therapy taking the given patient information in consideration.

If endocrine treatment is advisable, please provide a suitable treatment regimen.

If systemic treatment is advisable, please provide a suitable treatment regimen.

If radiation therapy is advisable, please provide a suitable treatment regimen.

Based on the oncological family history, please state whether genetic testing should be performed or not.

| **Patient 12 Treatment Concepts** |  |  |
| --- | --- | --- |
| **Concept 1** | **Concept 2** | **Concept 3** |
| **Treatment Recommendations for the Patient**  **1. Further Surgical Treatment**  - No further surgical treatment is required as the patient has undergone breast-conserving surgery with sentinel lymph node biopsy, achieving clear resection margins (R0) with a minimal margin of 2 mm. This is considered adequate for local control.  **2. Systemic Treatment**  Given the patient's HER2-positive, hormone receptor-negative (ER 0%, PR 5%), high-grade (G3), and high-proliferation (Ki-67: 80%) tumor, systemic therapy is strongly indicated.  Neoadjuvant Therapy:  - Although the patient has already undergone surgery, the tumor's high-risk features (pT2, pN1, HER2-positive, G3) suggest the need for adjuvant systemic therapy to reduce the risk of recurrence.  **2.1 Adjuvant Chemotherapy**  - A non-anthracycline-based regimen is preferred due to the patient's history of antiphospholipid syndrome and pulmonary embolism, which increases the risk of thromboembolic events. Anthracyclines are associated with cardiotoxicity and may exacerbate thrombotic risks.  - Recommended regimen:  - Docetaxel (T) and Carboplatin (C) in combination with HER2-targeted therapy (trastuzumab and pertuzumab).  - Example: Docetaxel 75 mg/m² and Carboplatin AUC 6 every 3 weeks for 6 cycles, with concurrent trastuzumab and pertuzumab.  **2.2 HER2-Targeted Therapy**  - Trastuzumab and Pertuzumab should be continued for a total of 1 year (including during chemotherapy and afterward as maintenance therapy).  - Cardiac function (LVEF) should be monitored regularly due to the risk of cardiotoxicity.  **2.3 Anticoagulation Consideration**  - The patient is on phenprocoumon for antiphospholipid syndrome. Close monitoring of INR is essential during chemotherapy to avoid bleeding complications. Switching to low-molecular-weight heparin (LMWH) during chemotherapy may be considered to reduce drug interactions.  **3. Endocrine Treatment**  - Endocrine therapy is not indicated as the tumor is hormone receptor-negative (ER 0%, PR 5%).  **4. Radiation Therapy**  - Indicated due to breast-conserving surgery and the presence of nodal involvement (pN1).  - Recommended regimen:  - Whole-breast irradiation (WBI): 50 Gy in 25 fractions over 5 weeks.  - Tumor bed boost: Additional 10-16 Gy in 5-8 fractions to the tumor bed.  - Regional nodal irradiation (RNI): Include the supraclavicular and axillary lymph node regions due to pN1 status.  **5. Genetic Testing**  - The patient's oncological family history (father with colon cancer at 45, paternal grandmother with endometrial cancer, and paternal uncle with urothelial carcinoma) raises suspicion for a hereditary cancer syndrome.  - Genetic testing is recommended to evaluate for germline mutations, particularly in genes associated with hereditary breast and ovarian cancer (e.g., BRCA1/2) or Lynch syndrome (e.g., MLH1, MSH2, MSH6, PMS2).  **Summary of Treatment Plan**    1. No further surgery is required.  2. Adjuvant systemic therapy:  - Docetaxel + Carboplatin with trastuzumab and pertuzumab.  - Continue HER2-targeted therapy for 1 year.  3. Radiation therapy:  - Whole-breast irradiation with tumor bed boost and regional nodal irradiation.  4. Genetic testing:  - Recommended to assess for hereditary cancer syndromes.  This comprehensive approach addresses the patient's high-risk breast cancer while considering her thrombotic history and family history. | Here’s a concise, evidence-based adjuvant plan for this 42-year-old, premenopausal patient with pT2 pN1 M0, G3, HER2-positive (ISH+), ER 0%, PR 5%, Ki-67 80% invasive breast cancer after breast-conserving surgery (BCS) with a 2-mm negative margin and sentinel-node positivity.  **1) Further surgery**   - No re-excision needed. For invasive cancer treated with BCS, a negative margin defined as “no ink on tumor” is adequate; wider margins (e.g., 2 mm vs larger) don’t improve control. Your 2-mm R0 margin meets this standard. - Axilla:   - If only 1–2 positive sentinel nodes and you meet Z0011 criteria (T1–2, BCS, planned whole-breast RT and systemic therapy), omit completion axillary dissection and cover the axilla with regional nodal irradiation (RNI). AMAROS showed axillary RT gives axillary control comparable to ALND with less lymphedema.   - If ≥3 macrometastatic nodes and/or gross extranodal extension, most guidelines favor ALND or comprehensive RNI; discuss in tumor board. (Z0011 did not include these patients.)   **2) Systemic (anti-HER2 + chemotherapy)**  Indication: Node-positive, HER2-positive disease → adjuvant chemotherapy plus anti-HER2 therapy is standard. Add pertuzumab in node-positive patients (APHINITY).  Preferred regimen (non-anthracycline, to limit cardiotoxicity):   - TCHP x 6 cycles (q3w): docetaxel 75 mg/m² + carboplatin AUC 6 + trastuzumab (8 mg/kg load → 6 mg/kg q3w) + pertuzumab (840 mg load → 420 mg q3w). After 6 cycles, continue trastuzumab ± pertuzumab to complete 1 year of anti-HER2 therapy (IV or fixed-dose SC combo). Non-anthracycline TCH showed similar efficacy to AC→TH with less cardiac toxicity (BCIRG-006); APHINITY shows the added benefit of pertuzumab is concentrated in node-positive disease and now has OS benefit at 10 years.   Cardiac monitoring: Baseline LVEF, then every 3 months during trastuzumab, and every 6 months for 2 years after completing adjuvant trastuzumab (per label). Avoid anthracycline if any cardiac risk.  Anticoagulation considerations (antiphospholipid syndrome on phenprocoumon):   - Do not switch to a DOAC for convenience; DOACs are not recommended in APS (especially high-risk profiles). Continue VKA with close INR monitoring through chemotherapy.   Neratinib: Extended adjuvant neratinib benefits mainly hormone-receptor–positive patients; with ER 0% and only PR 5%, it’s generally not indicated here.  **3) Endocrine therapy**   - Technically, NCCN labels HR+ as ER and/or PR+, but the benefit of endocrine therapy is uncertain when ER is 0% with only low PR. ASCO/CAP notes limited benefit when receptor expression is very low and emphasizes ER as the predictive marker; PR alone has poor predictive value. Given ER 0%, PR 5%, HER2+ biology, and the patient’s strong VTE history (APS with prior PE/DVT), I do not recommend routine endocrine therapy. - If, after shared decision-making, endocrine therapy is still desired, avoid tamoxifen (VTE risk). Use ovarian function suppression (e.g., goserelin 3.6 mg q28d or 10.8 mg q12wk) + aromatase inhibitor (e.g., letrozole 2.5 mg daily) for 5 years; check bone density and manage bone health. Tamoxifen increases VTE risk and is ill-advised in APS.   **4) Radiation therapy (after chemotherapy; can overlap with trastuzumab)**   - Whole-breast irradiation (WBI) is mandatory after BCS. Use moderate hypofractionation as standard: 40 Gy in 15 fractions (3 weeks), then a tumor-bed boost (e.g., 10–16 Gy in 4–8 fractions), given her age (42) and grade 3. - Regional nodal irradiation (RNI) is recommended for pN1 (1–3 nodes) with adverse features (young age, T2, high grade, HER2+): treat axillary level III/supraclavicular and consider internal mammary nodes. MA.20 and EORTC 22922 show RNI reduces recurrences and breast-cancer mortality (with modest or no OS effect overall). Hypofractionated RNI (e.g., 40 Gy/15 fx concurrently with WBI) is acceptable in modern practice.   **5) Practical sequencing**   1. Baseline: history/physical, labs, ECHO, pregnancy test if relevant; port only if needed. 2. TCHP x 6 (18 weeks). 3. Start WBI + RNI soon after chemo; continue trastuzumab±pertuzumab during RT. 4. Complete anti-HER2 to 1 year. Cardiac surveillance per label.   **6) Genetic testing**   - Yes — recommend germline testing and genetic counseling. Rationale: young age (42) at breast-cancer diagnosis meets major-guideline thresholds for testing; use a multigene breast cancer panel (e.g., BRCA1/2, PALB2, TP53, CHEK2, ATM, etc.). - Family history also suggests possible Lynch syndrome (colorectal cancer at 45 in father; endometrial cancer in grandmother; renal pelvis urothelial carcinoma in uncle). Refer for Lynch syndrome risk assessment and consider including MMR genes (MLH1, MSH2, MSH6, PMS2, EPCAM) in testing. (Family-history–based referral is supported by ESMO/NCCN pathways.)   **Why these choices fit this patient**   - High-risk, node-positive HER2+ disease merits chemotherapy + dual anti-HER2; APHINITY’s long-term data now show OS benefit in node-positive patients. A non-anthracycline backbone (TCHP) keeps efficacy while reducing cardiac risk, important when giving a year of HER2 therapy. - Endocrine therapy: with ER 0% and only PR 5%, the expected benefit is minimal, while tamoxifen is contraindicated by her thrombotic history; if ET is pursued after counseling, choose OFS + AI rather than tamoxifen. - Radiation: BCS mandates WBI; RNI is appropriate for pN1 with high-risk features. Modern trials support hypofractionation and IM-SC (internal mammary/supraclavicular) coverage in selected patients. - Anticoagulation: Continue VKA (phenprocoumon) rather than a DOAC in APS; monitor INR closely during chemo.   **One-page prescribing summary (for the team)**   - Chemo/anti-HER2: TCHP q3w × 6 → continue trastuzumab ± pertuzumab to 12 months total. - Cardiac checks: ECHO q3 months on therapy; q6 months × 2 years after. - Radiation: WBI 40 Gy/15 fx + boost 10–16 Gy, with RNI (levels II–III/SCV ± IMN) ~40 Gy/15 fx. - Endocrine: Not routinely recommended; if chosen → OFS + AI for 5 years; avoid tamoxifen. - Anticoagulation: Remain on VKA (phenprocoumon); avoid DOACs in APS. - Genetics: Refer for multigene breast panel; also evaluate for Lynch given family history. | This patient has stage II (pT2pN1M0), high-grade (G3), HER2-positive, hormone receptor-negative (ER 0%, PR 5%) breast cancer, with a history of antiphospholipid syndrome and permanent anticoagulation. The following evidence-based recommendations apply:  **Further surgical treatment:**  No further breast surgery is indicated, as margins are negative (R0, 2 mm) and axillary staging has been performed. This is consistent with standard practice for breast-conserving surgery in early-stage disease.  **Endocrine treatment:**  Endocrine therapy is not indicated due to the lack of estrogen receptor expression (ER 0%) and minimal progesterone receptor positivity (PR 5%), which is below the threshold for benefit.  **Systemic treatment:**  Adjuvant chemotherapy plus anti-HER2 therapy is standard. The American Society of Clinical Oncology recommends a combination of anthracycline- and taxane-based chemotherapy with trastuzumab for node-positive, HER2-positive disease. Pertuzumab may be added for higher-risk cases (≥1 node positive, high grade).  • Chemotherapy regimen: Dose-dense AC (doxorubicin/cyclophosphamide) followed by paclitaxel, with trastuzumab initiated with the taxane phase.  • Anti-HER2 therapy: Trastuzumab for 1 year; consider adding pertuzumab.  • Anticoagulation: Anthracyclines and taxanes are not contraindicated in patients with antiphospholipid syndrome, but careful monitoring for thrombosis and bleeding is required.  **Radiation therapy:**  Adjuvant whole-breast irradiation is indicated after breast-conserving surgery, with a boost to the tumor bed due to high-grade disease and close margins. Regional nodal irradiation should be considered due to nodal involvement (pN1).  **Genetic testing:**  Genetic testing is recommended due to the strong family history of early-onset colon, endometrial, and urothelial cancers, which may suggest a hereditary cancer syndrome (e.g., Lynch syndrome, BRCA, or other germline mutations).  **In summary:**  The patient should receive adjuvant chemotherapy (anthracycline/taxane), trastuzumab (± pertuzumab), and radiation therapy, with genetic counseling and testing. Endocrine therapy is not indicated.  All recommendations are supported by the American Society of Clinical Oncology and current review articles. |

| **PATIENT CASE 12** |  | |  | |  |  |  |  |
| --- | --- | --- | --- | --- | --- | --- | --- | --- |
|  |  | |  | |  |  |  |  |
| **How difficult or easy was the patient case?** | **very easy** | | **easy** | | **moderate** | **difficult** | **very difficult** | **Score** |
| **Case 12** | **1** | | **2** | | **3** | **4** | **5** |  |
|  |  | |  | |  |  |  |  |
| **The therapy concept is safe.** | **strongly disagree** | | **disagree** | | **neither agree nor disagree** | **agree** | **strongly agree** | **Score** |
| **Therapy Concept 1** | **1** | | **2** | | **3** | **4** | **5** |  |
| **Therapy Concept 2** | **1** | | **2** | | **3** | **4** | **5** |  |
| **Therapy Concept 3** | **1** | | **2** | | **3** | **4** | **5** |  |
|  |  | |  | |  |  |  |  |
| **The therapy concept is in line with the current guidelines.** | **strongly disagree** | | **disagree** | | **neither agree nor disagree** | **agree** | **strongly agree** | **Score** |
| **Therapy Concept 1** | **1** | | **2** | | **3** | **4** | **5** |  |
| **Therapy Concept 2** | **1** | | **2** | | **3** | **4** | **5** |  |
| **Therapy Concept 3** | **1** | | **2** | | **3** | **4** | **5** |  |
|  |  | |  | |  |  |  |  |
| **The therapy concept is medically adequate.** | **strongly disagree** | | **disagree** | | **neither agree nor disagree** | **agree** | **strongly agree** | **Score** |
| **Therapy Concept 1** | **1** | | **2** | | **3** | **4** | **5** |  |
| **Therapy Concept 2** | **1** | | **2** | | **3** | **4** | **5** |  |
| **Therapy Concept 3** | **1** | | **2** | | **3** | **4** | **5** |  |
|  |  | |  | |  |  |  |  |
| **The therapy concept is complete.** | **strongly disagree** | | **disagree** | | **neither agree nor disagree** | **agree** | **strongly agree** | **Score** |
| **Therapy Concept 1** | **1** | | **2** | | **3** | **4** | **5** |  |
| **Therapy Concept 2** | **1** | | **2** | | **3** | **4** | **5** |  |
| **Therapy Concept 3** | **1** | | **2** | | **3** | **4** | **5** |  |
|  |  | |  | |  |  |  |  |
| **The therapy concept is of high quality.** | **strongly disagree** | | **disagree** | | **neither agree nor disagree** | **agree** | **strongly agree** | **Score** |
| **Therapy Concept 1** | **1** | | **2** | | **3** | **4** | **5** |  |
| **Therapy Concept 2** | **1** | | **2** | | **3** | **4** | **5** |  |
| **Therapy Concept 3** | **1** | | **2** | | **3** | **4** | **5** |  |
|  |  | |  | |  |  |  |  |
| **The reasoning behind the treatment decision is logical.** | **strongly disagree** | | **disagree** | | **neither agree nor disagree** | **agree** | **strongly agree** | **Score** |
| **Therapy Concept 1** | **1** | | **2** | | **3** | **4** | **5** |  |
| **Therapy Concept 2** | **1** | | **2** | | **3** | **4** | **5** |  |
| **Therapy Concept 3** | **1** | | **2** | | **3** | **4** | **5** |  |
|  |  | |  | |  |  |  |  |
| **Please rank the therapy concept according to your preference.** | | **Ranking (1^st^, 2^nd^, 3^rd^)** | |  |  |  |  |  |
| **Therapy Concept 1** | |  | |  |  |  |  |  |
| **Therapy Concept 2** | |  | |  |  |  |  |  |
| **Therapy Concept 3** | |  | |  |  |  |  |  |

**Patient 13**

How should the following breast cancer patient be treated based on the most relevant and current international literature?

Patient information:

Age: 56

Menopause status: postmenopausal

ECOG: 1

Previous illness: Insulin-dependent diabetes mellitus type 2, obesity with BMI of 43, obstructive sleep apnea syndrome, secondary arterial hypertension (with ACE inhibitor medication)

Previous surgical treatment: Abdominoplasty at the age of 50, bilateral mammary reduction mammoplasty for mammary hypertrophy at the age of 35

Birth history: 3 vaginal births at the age of 20, 21 and 25

Oncological family history: Mother with breast cancer at the age of 40

The patient underwent the following surgical treatment: Breast-conserving tumorectomy with sentinellymphnodectomy on the left side

Following the surgical treatment, the following TNM-classification, resection margin, histological classification, grading and immunohistology were identified:

TNM: pT1apN0M0

Resection margin: R0, minimal resection margin of 1 mm

Histological classification: NST

Grading: G2

Multifocality/-centricity: Monofocal and -centric

Estrogen receptor (ER): 0%

Progesterone receptor (PR): 0%

Her2-status: Negative (IHC 1+)

Ki-67-proliferation-index: 40%

Please provide a step-by-step treatment recommendation with regard to further surgical treatment, endocrine treatment, systemic treatment and radiation therapy taking the given patient information in consideration.

If endocrine treatment is advisable, please provide a suitable treatment regimen.

If systemic treatment is advisable, please provide a suitable treatment regimen.

If radiation therapy is advisable, please provide a suitable treatment regimen.

Based on the oncological family history, please state whether genetic testing should be performed or not.

| **Patient 13 Treatment Concepts** |  |  |
| --- | --- | --- |
| **Concept 1** | **Concept 2** | **Concept 3** |
| **Treatment Recommendations for the Patient**  **1. Further Surgical Treatment**  - No further surgical treatment is required as the patient underwent breast-conserving tumorectomy with sentinel lymph node biopsy, achieving clear resection margins (R0) with a minimal margin of 1 mm. The tumor was monofocal and -centric, and there is no evidence of lymph node involvement (pN0).  **2. Endocrine Treatment**  - Endocrine therapy is not indicated as the tumor is estrogen receptor (ER)-negative and progesterone receptor (PR)-negative. Hormone receptor-negative tumors do not benefit from endocrine treatment.  **3. Systemic Treatment**  - The tumor is triple-negative (ER-negative, PR-negative, HER2-negative) with a high Ki-67 proliferation index (40%), indicating an aggressive phenotype. Based on current guidelines, adjuvant chemotherapy is recommended for triple-negative breast cancer (TNBC) even in early-stage disease (pT1a) if the tumor is ≥0.5 cm in size and/or has high-risk features such as high Ki-67.    Recommended Chemotherapy Regimen:  - A taxane- and anthracycline-based regimen is preferred. For example:  - Doxorubicin (60 mg/m²) + Cyclophosphamide (600 mg/m²) every 3 weeks for 4 cycles, followed by  - Paclitaxel (80 mg/m²) weekly for 12 weeks or Docetaxel (75-100 mg/m²) every 3 weeks for 4 cycles.  - Dose adjustments may be necessary due to the patient’s comorbidities (e.g., diabetes, obesity). Close monitoring for chemotherapy-related toxicities is essential.  **4. Radiation Therapy**  - Radiation therapy is indicated following breast-conserving surgery to reduce the risk of local recurrence.    Recommended Radiation Therapy Regimen:  - Whole-breast irradiation (WBI) with a total dose of 40-50 Gy delivered in fractions of 2-2.67 Gy over 3-5 weeks.  - A tumor bed boost of 10-16 Gy may be considered, especially given the minimal resection margin of 1 mm.  - Careful planning is required to minimize radiation exposure to the heart and lungs, particularly in a patient with obesity and comorbidities.  **5. Genetic Testing**  - Given the patient’s family history of breast cancer (mother diagnosed at age 40), genetic testing for hereditary breast cancer syndromes (e.g., BRCA1/BRCA2 mutations) is recommended.  - If a pathogenic mutation is identified, this may have implications for the patient’s future surveillance and management, as well as for her family members.  **6. Additional Considerations**  - Comorbidity Management:  - Optimize control of diabetes, hypertension, and obstructive sleep apnea to reduce the risk of treatment-related complications.  - Consider a multidisciplinary approach involving endocrinology, cardiology, and nutrition specialists.  - Lifestyle Modifications:  - Encourage weight management and physical activity to improve overall prognosis and reduce the risk of recurrence.  **Summary of Recommendations**  1. No further surgical treatment is required.  2. Endocrine therapy is not indicated.  3. Adjuvant chemotherapy with a taxane- and anthracycline-based regimen is recommended.  4. Whole-breast irradiation with a tumor bed boost is advised.  5. Genetic testing for hereditary breast cancer syndromes is recommended. | Here’s a clear, guideline-concordant plan for this patient with small, node-negative, triple-negative breast cancer (TNBC) after breast-conserving surgery.  **1) Further surgery**   - No re-excision needed. For invasive cancer treated with breast-conserving therapy, a negative margin = “no ink on tumor”; wider margins (e.g., >1–2 mm) do not further lower local recurrence when whole-breast RT is given. A 1 mm minimal margin therefore meets the modern margin standard. Sentinel node is negative, so no further axillary surgery.   **2) Endocrine therapy**   - Not indicated. Tumor is ER 0% / PR 0% (triple-negative). (Standard ESMO/ASCO/NCCN principle.)   **3) Systemic (drug) therapy**   - Adjuvant chemotherapy: *not recommended* for pT1aN0 (≤5 mm) TNBC in the absence of other very high-risk features. Major international guidelines (NCCN/ESMO/ASCO) generally do not recommend chemo for T1aN0 TNBC; benefit has not been shown and absolute risk is very low. Given this patient’s comorbidities (insulin-dependent T2D, BMI 43, OSA, hypertension), the risk-benefit tilts even further away from chemo.   - Notes for context (no action needed): for T1b (0.6–1.0 cm) chemo may be considered; for T1c (1.1–2.0 cm) chemo is generally recommended. Pembrolizumab and/or capecitabine are for stage II–III or residual-disease settings—not for stage I T1aN0. - PARP inhibitor (olaparib): not applicable. Even if a germline BRCA1/2 variant were found, OlympiA eligibility in the upfront-surgery cohort required ≥T2 or node-positive disease (or residual invasive disease after neoadjuvant therapy). This T1aN0 tumor does not qualify.   **4) Radiation therapy (adjuvant)**  Indicated after breast-conserving surgery.  Recommended approach:   - Whole-breast irradiation (WBI), hypofractionated (preferred by ASTRO/ESMO/NCCN for essentially all suitable patients):   - Option A (ultra-hypofractionated, FAST-Forward): 26 Gy in 5 fractions over 1 week.   - Option B (moderate hypofractionation): 40 Gy in 15 fractions over 3 weeks (or 42.5 Gy in 16). - Tumor-bed boost: recommend/strongly consider. Although she is 56 (not ≤50) and G2, TNBC biology and a close 1 mm margin favor a boost for maximal local control. Typical regimens: 10–16 Gy in 4–8 fractions sequentially after WBI. Evidence shows a boost reduces local recurrence (trade-off: higher fibrosis risk). - Fields: Whole breast only; no regional nodal irradiation for pT1aN0 without other risk factors. (Standard practice per WBI guidelines.) - Technique / planning pearls for this patient:   - Left-sided tumor → use deep-inspiration breath-hold (DIBH) and heart-sparing tangents to minimize mean heart dose; consider prone positioning to improve dose homogeneity given large body habitus. (Endorsed in ASTRO WBI technical guidance.)   **5) Genetic testing**   - Yes—test. Current guidance recommends germline testing for patients with TNBC diagnosed at ≤65 years, and/or with a significant family history; this patient is 56 with a mother who had breast cancer at 40. Order a multigene panel (e.g., BRCA1/2, PALB2, ATM, CHEK2, etc.) with pre-/post-test counseling. (Note: even if positive, adjuvant olaparib is not indicated for stage I, but results affect surveillance and family risk.)   **Practical, step-by-step plan**   1. No additional surgery. Document margins as adequate (“no ink on tumor”); no axillary RT or surgery. 2. No endocrine therapy (triple-negative). 3. No adjuvant chemotherapy (pT1aN0 TNBC). Document shared decision noting guideline stance and comorbidity-related toxicity risk. 4. Adjuvant radiotherapy:    - Choose 26 Gy/5fx (1 week) *or* 40 Gy/15fx (3 weeks) to the whole breast.    - Add tumor-bed boost 10–16 Gy (e.g., 10 Gy in 4–5 fx) given TN phenotype and 1 mm margin.    - Use DIBH (left-sided), prioritize homogeneity; consider prone setup. 5. Genetics: refer for germline multigene panel testing and counseling.   **Notes on follow-up & supportive care (brief)**   - Surveillance: clinical breast exam every 6–12 months for 5 years, then annually; annual mammography of both breasts. (Follow your local society guidance.) - RT skin care: proactive skin-care plan; consider topical steroid if brisk reaction given BMI. - Cardiometabolic risk: optimize diabetes and blood pressure control during RT. | This patient has triple-negative breast cancer (TNBC) (ER 0%, PR 0%, HER2-negative), stage pT1a pN0 M0, grade 2, postmenopausal, with significant comorbidities. The most relevant and current international literature supports the following management:  **Further surgical treatment:**  No further breast or axillary surgery is indicated. The patient has undergone breast-conserving surgery with clear margins (R0) and sentinel node dissection with pN0 status, which is standard for early-stage disease.  **Endocrine therapy:**  Endocrine therapy is not indicated for TNBC, as the tumor is negative for both estrogen and progesterone receptors. The American Society of Clinical Oncology (ASCO) guideline and NEJM review confirm that endocrine therapy is only beneficial for hormone receptor-positive disease.  **Systemic therapy:**  Adjuvant chemotherapy is generally not recommended for pT1aN0 TNBC (tumor ≤0.5 cm, node-negative) due to the very low absolute benefit and increased risk of toxicity, especially in patients with significant comorbidities such as diabetes, obesity, and hypertension. The ASCO guideline states that patients with cT1aN0 TNBC should not routinely be offered chemotherapy. However, the high Ki-67 (40%) and grade 2 status are noted, but do not override the size and node status in this context. If the tumor were larger (≥T1b) or had other high-risk features, chemotherapy would be considered.  **Radiation therapy:**  Adjuvant whole-breast radiotherapy is recommended following breast-conserving surgery for all patients with invasive breast cancer, including TNBC, to reduce local recurrence risk. Standard regimens include hypofractionated schedules (e.g., 40 Gy in 15 fractions over 3 weeks), unless contraindicated by anatomy or comorbidities.  **Genetic testing:**  Genetic testing for BRCA1/2 mutations is indicated due to the patient’s triple-negative phenotype and a first-degree relative (mother) diagnosed with breast cancer at age 40. The ASCO guideline and current literature support testing in such cases, as it may impact future management and family counseling.  **In summary:**  • No further surgery  • No endocrine therapy  • No adjuvant chemotherapy  • Adjuvant whole-breast radiotherapy  • Genetic testing for BRCA1/2 is recommended |

| **PATIENT CASE 13** |  | |  | |  |  |  |  |
| --- | --- | --- | --- | --- | --- | --- | --- | --- |
|  |  | |  | |  |  |  |  |
| **How difficult or easy was the patient case?** | **very easy** | | **easy** | | **moderate** | **difficult** | **very difficult** | **Score** |
| **Case 13** | **1** | | **2** | | **3** | **4** | **5** |  |
|  |  | |  | |  |  |  |  |
| **The therapy concept is safe.** | **strongly disagree** | | **disagree** | | **neither agree nor disagree** | **agree** | **strongly agree** | **Score** |
| **Therapy Concept 1** | **1** | | **2** | | **3** | **4** | **5** |  |
| **Therapy Concept 2** | **1** | | **2** | | **3** | **4** | **5** |  |
| **Therapy Concept 3** | **1** | | **2** | | **3** | **4** | **5** |  |
|  |  | |  | |  |  |  |  |
| **The therapy concept is in line with the current guidelines.** | **strongly disagree** | | **disagree** | | **neither agree nor disagree** | **agree** | **strongly agree** | **Score** |
| **Therapy Concept 1** | **1** | | **2** | | **3** | **4** | **5** |  |
| **Therapy Concept 2** | **1** | | **2** | | **3** | **4** | **5** |  |
| **Therapy Concept 3** | **1** | | **2** | | **3** | **4** | **5** |  |
|  |  | |  | |  |  |  |  |
| **The therapy concept is medically adequate.** | **strongly disagree** | | **disagree** | | **neither agree nor disagree** | **agree** | **strongly agree** | **Score** |
| **Therapy Concept 1** | **1** | | **2** | | **3** | **4** | **5** |  |
| **Therapy Concept 2** | **1** | | **2** | | **3** | **4** | **5** |  |
| **Therapy Concept 3** | **1** | | **2** | | **3** | **4** | **5** |  |
|  |  | |  | |  |  |  |  |
| **The therapy concept is complete.** | **strongly disagree** | | **disagree** | | **neither agree nor disagree** | **agree** | **strongly agree** | **Score** |
| **Therapy Concept 1** | **1** | | **2** | | **3** | **4** | **5** |  |
| **Therapy Concept 2** | **1** | | **2** | | **3** | **4** | **5** |  |
| **Therapy Concept 3** | **1** | | **2** | | **3** | **4** | **5** |  |
|  |  | |  | |  |  |  |  |
| **The therapy concept is of high quality.** | **strongly disagree** | | **disagree** | | **neither agree nor disagree** | **agree** | **strongly agree** | **Score** |
| **Therapy Concept 1** | **1** | | **2** | | **3** | **4** | **5** |  |
| **Therapy Concept 2** | **1** | | **2** | | **3** | **4** | **5** |  |
| **Therapy Concept 3** | **1** | | **2** | | **3** | **4** | **5** |  |
|  |  | |  | |  |  |  |  |
| **The reasoning behind the treatment decision is logical.** | **strongly disagree** | | **disagree** | | **neither agree nor disagree** | **agree** | **strongly agree** | **Score** |
| **Therapy Concept 1** | **1** | | **2** | | **3** | **4** | **5** |  |
| **Therapy Concept 2** | **1** | | **2** | | **3** | **4** | **5** |  |
| **Therapy Concept 3** | **1** | | **2** | | **3** | **4** | **5** |  |
|  |  | |  | |  |  |  |  |
| **Please rank the therapy concept according to your preference.** | | **Ranking (1^st^, 2^nd^, 3^rd^)** | |  |  |  |  |  |
| **Therapy Concept 1** | |  | |  |  |  |  |  |
| **Therapy Concept 2** | |  | |  |  |  |  |  |
| **Therapy Concept 3** | |  | |  |  |  |  |  |

**Patient 14**

How should the following breast cancer patient be treated based on the most relevant and current international literature?

Patient information:

Age: 65

Menopause status: postmenopausal

ECOG: 1

Previous illness: Addison's disease (currently under hydro- and fludrocortisone medication)

Previous surgical treatment: Total knee replacement on the left side at the age of 50

Birth history: 3 cesareans at the age of 25, 28 and 35

Oncological family history: Paternal grandmother with pancreatic cancer at the age of 59, maternal aunt with colon cancer at the age of 60

The patient did not undergo surgery so far.

Following the surgical biopsy and staging, the following information about the tumor is available:

TNM: cT3pN+pM1 (OSS)

Histological classification: NST

Grading: G3

Estrogen receptor (ER): 0%

Progesterone receptor (PR): 0%

Her2-status: Negative (IHC 0)

Ki-67-proliferation-index: 60%

Please provide a step-by-step treatment recommendation with regard to the necessity of neoadjuvant treatment, present or future surgical treatment, endocrine treatment, systemic treatment and radiation therapy taking the given patient information into consideration.

If endocrine treatment is advisable, please provide a suitable treatment regimen.

If systemic treatment is advisable, please provide a suitable treatment regimen.

If radiation therapy is advisable, please provide a suitable treatment regimen.

Based on the oncological family history, please state whether genetic testing should be performed or not.

| **Patient 14 Treatment Concepts** |  |  |
| --- | --- | --- |
| **Concept 1** | **Concept 2** | **Concept 3** |
| **Treatment Recommendation for the Patient**  **1. Overview of Diagnosis and Prognostic Factors**  The patient is a 65-year-old postmenopausal woman with triple-negative breast cancer (TNBC) classified as cT3pN+pM1 (OSS), indicating a locally advanced tumor with regional lymph node involvement and distant metastasis to the bone (osseous metastasis). The tumor is high-grade (G3), with a high Ki-67 proliferation index (60%), suggesting aggressive biology. The patient has a history of Addison's disease, which requires careful management during systemic therapy.  **2. Neoadjuvant Treatment**  - Recommendation: Neoadjuvant systemic therapy is not indicated in this case due to the presence of distant metastasis (M1). The primary goal of treatment in metastatic TNBC is palliative, focusing on systemic control and symptom management rather than surgical downstaging.  **3. Systemic Treatment**  - Primary Approach: Systemic chemotherapy is the cornerstone of treatment for metastatic TNBC. The following regimen is recommended:  - First-line chemotherapy: A combination of anthracycline and taxane-based regimens is standard. Suggested regimen:  - Doxorubicin (Adriamycin): 60 mg/m² IV on day 1  - Cyclophosphamide: 600 mg/m² IV on day 1  - Administer every 21 days for 4 cycles, followed by:  - Paclitaxel: 80 mg/m² IV weekly for 12 weeks  - Alternative regimen: If anthracyclines are contraindicated due to cardiac risk, consider carboplatin-based therapy:  - Carboplatin (AUC 5): IV on day 1  - Paclitaxel: 80 mg/m² IV weekly for 12 weeks  - Additional Considerations:  - PD-L1 Testing: If the tumor expresses PD-L1 (via immunohistochemistry), consider adding an immune checkpoint inhibitor such as Atezolizumab in combination with nab-paclitaxel.  - Bone-Directed Therapy: Given the osseous metastasis, initiate zoledronic acid (4 mg IV every 4 weeks) or denosumab (120 mg subcutaneously every 4 weeks) to reduce skeletal-related events.  **4. Surgical Treatment**  - Recommendation: Surgery is not indicated in the metastatic setting unless for palliative purposes, such as managing local complications (e.g., ulceration, bleeding, or infection). The focus remains on systemic therapy.  **5. Radiation Therapy**  - Recommendation: Palliative radiation therapy should be considered for symptomatic bone metastases or to prevent complications such as pathological fractures or spinal cord compression.  -Suggested regimen:  - Dose: 20 Gy in 5 fractions or 30 Gy in 10 fractions to the affected osseous sites.  **6. Endocrine Treatment**  - Recommendation: Endocrine therapy is not applicable as the tumor is ER-negative and PR-negative.  **7. Genetic Testing**  - Recommendation: Genetic testing is warranted based on the patient’s triple-negative breast cancer diagnosis and family history of pancreatic and colon cancer, which raises suspicion for hereditary cancer syndromes such as BRCA1/2 mutations or Lynch syndrome. Testing should include:  - BRCA1/2 mutation analysis  - Multigene panel testing for other hereditary cancer-related genes (e.g., PALB2, TP53).  **8. Supportive Care**  - Management of Addison’s Disease: Ensure adequate glucocorticoid and mineralocorticoid replacement during systemic therapy. Stress-dose steroids may be required during chemotherapy to prevent adrenal crisis.  - Symptom Management: Address pain, fatigue, and other symptoms associated with metastatic disease through a multidisciplinary palliative care approach.  **Summary of Treatment Plan**  1. Systemic Therapy: Anthracycline- and taxane-based chemotherapy, with consideration of carboplatin or immune checkpoint inhibitors if PD-L1 positive.  2. Radiation Therapy: Palliative radiation for symptomatic bone metastases.  3. Genetic Testing: BRCA1/2 and multigene panel testing.  4. Supportive Care: Bone-directed therapy, adrenal insufficiency management, and palliative care.  This approach aligns with current international guidelines for metastatic TNBC and considers the patient’s comorbidities and overall condition. | Below is a practical, guideline-concordant plan for a de-novo stage IV, triple-negative breast cancer (TNBC) with bone metastasis in a fit, postmenopausal 65-year-old (ECOG 1) who has Addison’s disease on physiologic steroid replacement.  **1) What this means up front**   - Phenotype: ER-/PR-/HER2- (IHC 0), Ki-67 60% → TNBC. - Stage: cT3 pN+ pM1 (oss) → de-novo metastatic disease with bone involvement. - Goal of care: disease control, symptom relief, and survival prolongation with systemic therapy first. “Neoadjuvant” concepts don’t apply in de-novo stage IV. Routine upfront breast/axilla surgery does not improve overall survival and is reserved for symptom control or complications.   **2) Essential tests before/while starting therapy**   1. PD-L1 (CPS by 22C3 pharmDx). If CPS ≥10, add pembrolizumab to first-line chemotherapy (see §3). 2. Germline testing (at least BRCA1/2; consider multigene panel incl. PALB2). All patients with HER2-negative metastatic breast cancer who could receive a PARP inhibitor should be offered gBRCA1/2 testing regardless of age/family history; your family history (pancreatic cancer in a relative) further supports testing. 3. Consider broad NGS on tumor (to identify rare MSI-H/TMB-high or NTRK fusions with tumor-agnostic options). Use when results might change therapy sequencing.   **3) First-line systemic treatment (start now)**  A) If PD-L1 CPS ≥10   - Pembrolizumab + chemotherapy (KEYNOTE-355 standard of care):   - Pembrolizumab 200 mg IV q3 weeks (or 400 mg q6 weeks) with ONE of:     - nab-paclitaxel 100 mg/m² IV d1,8,15 q28 (preferred here to avoid steroid premeds; see Addison’s notes), or     - gemcitabine 1000 mg/m² + carboplatin AUC2 d1,8 q21.   - Continue pembrolizumab to progression/toxicity (up to ~2 years in trial), chemo per tolerance/response. This regimen improves OS versus chemo alone in CPS ≥ 10 mTNBC.   Why nab-paclitaxel over paclitaxel/docetaxel in this patient? Paclitaxel and docetaxel typically require corticosteroid premedication, whereas nab-paclitaxel generally does not, minimizing super-physiologic steroid exposure in a patient with Addison’s disease and while receiving immunotherapy.  B) If PD-L1 CPS <10 (or immunotherapy not appropriate)   - Chemotherapy alone. Reasonable options:   - nab-paclitaxel 100 mg/m² d1,8,15 q28, or   - gemcitabine + carboplatin as above (platinum is active in TNBC). (Anthracyclines are also active but consider age/cardiac risk and the good tolerability of the above options.)   C) If gBRCA1/2-mutated (HER2-neg) at any point   - Add a PARP inhibitor to your treatment sequence:   - Olaparib 300 mg PO BID or talazoparib 1 mg PO daily. Both improve outcomes vs chemo in gBRCA HER2-neg MBC; many clinicians use them as first- or second-line depending on PD-L1 status and prior therapy.   D) Upon progression after ≥1 prior line in the metastatic setting   - Sacituzumab govitecan 10 mg/kg IV d1 & d8 q21 (ASCENT) — robust PFS/OS benefit vs single-agent chemo; benefit maintained in patients ≥65.   **4.) Endocrine therapy:**  Not indicated (ER/PR 0%)  **5) Bone-targeted therapy (start with systemic therapy)**  Because there are bone metastases:   - Choose one:   - Denosumab 120 mg SC q4 weeks, or   - Zoledronic acid 4 mg IV q4 weeks; after several months, many de-escalate to q12 weeks (non-inferior in trials). - Add calcium + vitamin D, check serum calcium, and get a dental evaluation before first dose (ONJ risk). Denosumab is preferred in renal impairment; zoledronic acid allows evidence-based q12w dosing.   **6) Radiation therapy (as needed)**  Indications: painful/impending-fracture bone lesions, spinal canal compromise, or focal progression causing symptoms.   - Conventional EBRT (palliation): 8 Gy × 1 fraction is guideline-endorsed and convenient; alternatives 20 Gy in 5 or 30 Gy in 10 for selected cases (e.g., weight-bearing bones at higher fracture risk). - SBRT for oligometastatic lesions (selected cases, especially spine): commonly used regimens include 24 Gy × 1, 24–30 Gy in 3, or 35 Gy in 5, after multidisciplinary review. - Primary breast/axilla: radiate only for local symptoms (bleeding, pain, ulceration). Routine local therapy of the intact primary does not improve survival in de-novo stage IV.   **7) Surgery**   - Not recommended upfront for survival benefit in de-novo metastatic disease. Consider only for palliation/complications (e.g., uncontrolled breast symptoms; orthopedic stabilization for impending fracture).   **8) Special considerations for Addison’s disease**   - Immunotherapy is allowable in patients with pre-existing autoimmune disease when disease is minimally active and replacement-dose steroids are used, but it carries higher flare/irAE risk and requires close monitoring with your endocrinologist. - Aim to keep baseline steroids at physiologic doses (≈ 15–20 mg hydrocortisone/day ≈ ≤ 5 mg prednisone/day); baseline ≥10 mg prednisone-equivalent daily at ICI start is associated with worse outcomes. This is why we prefer nab-paclitaxel or carbo-gemcitabine over taxanes that need high-dose dexamethasone premeds. Use stress-dose steroids only when clinically indicated. - If you receive paclitaxel/docetaxel at any point, discuss steroid-sparing or minimized premedication strategies; standard paclitaxel premed includes dexamethasone and H1/H2 blockers, whereas nab-paclitaxel typically needs no premedication.   **9) Follow-up & supportive care**   - Re-stage every ~8–12 weeks (CT ± bone scan/PET per local practice) to judge response and adjust therapy. - Analgesia, physical therapy, and fracture precautions for weight-bearing lesions; involve orthopedics early if cortical breach/impending fracture. - Monitor marrow, renal function, electrolytes (esp. calcium with denosumab), and endocrine labs while on ICI.   **10) Summary of concrete regimens to choose from**  If PD-L1 CPS ≥ 10 (preferred):   - Pembrolizumab 200 mg IV q3 wk (or 400 mg q6 wk) +   - nab-paclitaxel 100 mg/m² IV d1,8,15 q28 or   - gemcitabine 1000 mg/m² + carboplatin AUC2 d1,8 q21. Continue until progression/toxicity (pembro typically ≤2 years).   If PD-L1 CPS < 10 / ICI not used:   - nab-paclitaxel 100 mg/m² d1,8,15 q28 or carbo-gem as above.   If gBRCA1/2-mutated (any line):   - Olaparib 300 mg BID or Talazoparib 1 mg daily.   After progression on the above:   - Sacituzumab govitecan 10 mg/kg IV d1 & d8 q21.   Bone protection (start now):   - Denosumab 120 mg SC q4 wk or Zoledronic acid 4 mg IV q4 wk (can later de-escalate to q12 wk).   Radiation (as needed):   - 8 Gy ×1 for painful sites; or 20 Gy/5 or 30 Gy/10. - SBRT 24 Gy×1 or 27–30 Gy/3 for selected oligometastases.   **11) Genetic testing — do it**  Yes. Order germline BRCA1/2 (and usually a multigene panel). Rationale: she has HER2-negative metastatic breast cancer and would be a candidate for PARP inhibitors if a gBRCA variant is present; ASCO recommends offering testing in this situation irrespective of age/family history, and her family history (pancreatic cancer in a relative) adds further indication. | This patient has metastatic triple-negative breast cancer (TNBC) (ER 0%, PR 0%, HER2 0) with bone metastasis, high grade (G3), and high proliferation (Ki-67 60%). The most relevant and current international literature, including guidelines from the American Society of Clinical Oncology (ASCO), supports the following approach:  **Neoadjuvant treatment:**  Neoadjuvant therapy is not indicated in metastatic (stage IV) breast cancer, as the disease is not curable and the goal is systemic control and palliation, not downstaging for surgery.  **Surgical treatment:**  Surgery is generally not recommended for the primary tumor in de novo metastatic breast cancer unless for palliation of symptoms (e.g., bleeding, ulceration, infection) or for local control in select cases. Systemic therapy is prioritized.  **Endocrine therapy:**  Endocrine therapy is not advisable because the tumor is ER and PR negative. The medical literature and ASCO guidelines state that endocrine therapy is only effective in hormone receptor-positive disease.  **Systemic therapy:**  First-line treatment is cytotoxic chemotherapy. Anthracycline- and taxane-based regimens are standard for metastatic TNBC, as recommended by the American Society of Clinical Oncology. Carboplatin may be considered, especially in high-risk or BRCA-mutated cases, but immune checkpoint inhibitors are not standard outside clinical trials for bone-only metastases. The patient’s ECOG 1 status and age are compatible with standard chemotherapy, but comorbid Addison’s disease requires careful monitoring and stress-dose steroids during cytotoxic therapy.  **Radiation therapy:**  Radiation therapy is indicated for palliation of symptomatic bone metastases (e.g., pain, risk of fracture, spinal cord compression). Dose and fractionation should be individualized based on symptoms and location.  **Genetic testing:**  Given the family history of pancreatic and colon cancer, and the diagnosis of TNBC, germline genetic testing for BRCA1/2 and other hereditary cancer syndromes is recommended. This is supported by the American Society of Clinical Oncology, as BRCA mutations may influence systemic therapy options (e.g., PARP inhibitors).  **In summary:**  Systemic chemotherapy is the mainstay of treatment, endocrine therapy is not indicated, radiation therapy is for palliation, and genetic testing should be performed due to family history and TNBC subtype. |

| **PATIENT CASE 14** |  | |  | |  |  |  |  |
| --- | --- | --- | --- | --- | --- | --- | --- | --- |
|  |  | |  | |  |  |  |  |
| **How difficult or easy was the patient case?** | **very easy** | | **easy** | | **moderate** | **difficult** | **very difficult** | **Score** |
| **Case 14** | **1** | | **2** | | **3** | **4** | **5** |  |
|  |  | |  | |  |  |  |  |
| **The therapy concept is safe.** | **strongly disagree** | | **disagree** | | **neither agree nor disagree** | **agree** | **strongly agree** | **Score** |
| **Therapy Concept 1** | **1** | | **2** | | **3** | **4** | **5** |  |
| **Therapy Concept 2** | **1** | | **2** | | **3** | **4** | **5** |  |
| **Therapy Concept 3** | **1** | | **2** | | **3** | **4** | **5** |  |
|  |  | |  | |  |  |  |  |
| **The therapy concept is in line with the current guidelines.** | **strongly disagree** | | **disagree** | | **neither agree nor disagree** | **agree** | **strongly agree** | **Score** |
| **Therapy Concept 1** | **1** | | **2** | | **3** | **4** | **5** |  |
| **Therapy Concept 2** | **1** | | **2** | | **3** | **4** | **5** |  |
| **Therapy Concept 3** | **1** | | **2** | | **3** | **4** | **5** |  |
|  |  | |  | |  |  |  |  |
| **The therapy concept is medically adequate.** | **strongly disagree** | | **disagree** | | **neither agree nor disagree** | **agree** | **strongly agree** | **Score** |
| **Therapy Concept 1** | **1** | | **2** | | **3** | **4** | **5** |  |
| **Therapy Concept 2** | **1** | | **2** | | **3** | **4** | **5** |  |
| **Therapy Concept 3** | **1** | | **2** | | **3** | **4** | **5** |  |
|  |  | |  | |  |  |  |  |
| **The therapy concept is complete.** | **strongly disagree** | | **disagree** | | **neither agree nor disagree** | **agree** | **strongly agree** | **Score** |
| **Therapy Concept 1** | **1** | | **2** | | **3** | **4** | **5** |  |
| **Therapy Concept 2** | **1** | | **2** | | **3** | **4** | **5** |  |
| **Therapy Concept 3** | **1** | | **2** | | **3** | **4** | **5** |  |
|  |  | |  | |  |  |  |  |
| **The therapy concept is of high quality.** | **strongly disagree** | | **disagree** | | **neither agree nor disagree** | **agree** | **strongly agree** | **Score** |
| **Therapy Concept 1** | **1** | | **2** | | **3** | **4** | **5** |  |
| **Therapy Concept 2** | **1** | | **2** | | **3** | **4** | **5** |  |
| **Therapy Concept 3** | **1** | | **2** | | **3** | **4** | **5** |  |
|  |  | |  | |  |  |  |  |
| **The reasoning behind the treatment decision is logical.** | **strongly disagree** | | **disagree** | | **neither agree nor disagree** | **agree** | **strongly agree** | **Score** |
| **Therapy Concept 1** | **1** | | **2** | | **3** | **4** | **5** |  |
| **Therapy Concept 2** | **1** | | **2** | | **3** | **4** | **5** |  |
| **Therapy Concept 3** | **1** | | **2** | | **3** | **4** | **5** |  |
|  |  | |  | |  |  |  |  |
| **Please rank the therapy concept according to your preference.** | | **Ranking (1^st^, 2^nd^, 3^rd^)** | |  |  |  |  |  |
| **Therapy Concept 1** | |  | |  |  |  |  |  |
| **Therapy Concept 2** | |  | |  |  |  |  |  |
| **Therapy Concept 3** | |  | |  |  |  |  |  |

**Patient 15**

How should the following breast cancer patient be treated based on the most relevant and current international literature?

Patient information:

Age: 29

Menopause status: premenopausal

ECOG: 0

Previous illness: None

Previous surgical treatment: None

Birth history: No prior birth

Oncological family history: Mother with breast cancer at the age of 65, maternal grandmother with breast cancer at the age of 70

The patient did not undergo surgery so far.

Following the surgical biopsy and staging, the following information about the tumor is available:

TNM: cT2cN0M0 on left side and cT1bcN0M0 on right side

Histological classification: NST on left and right side

Grading: G3

Estrogen receptor (ER): 0% on left and right side

Progesterone receptor (PR): 0% on left and right side

Her2-status: Negative (IHC 0) on left and right side

Ki-67-proliferation-index: 70% on left side, 85% on right side

Please provide a step-by-step treatment recommendation with regard to the necessity of neoadjuvant treatment, present or future surgical treatment, endocrine treatment, systemic treatment and radiation therapy taking the given patient information into consideration.

If endocrine treatment is advisable, please provide a suitable treatment regimen.

If systemic treatment is advisable, please provide a suitable treatment regimen.

If radiation therapy is advisable, please provide a suitable treatment regimen.

Based on the oncological family history, please state whether genetic testing should be performed or not.

| **Patient 15 Treatment Concepts** |  |  |
| --- | --- | --- |
| **Concept 1** | **Concept 2** | **Concept 3** |
| **Step-by-Step Treatment Recommendation**  **1. Neoadjuvant Treatment**  Given the patient's diagnosis of bilateral triple-negative breast cancer (TNBC) with high-grade tumors (G3) and high Ki-67 proliferation indices (70% and 85%), neoadjuvant systemic therapy is strongly recommended. This approach is supported by the following considerations:  - TNBC is an aggressive subtype with a high likelihood of response to chemotherapy.  - Neoadjuvant therapy allows for assessment of pathological complete response (pCR), which is a prognostic marker in TNBC.  - The tumor size (cT2 on the left and cT1b on the right) and bilateral involvement suggest a need for systemic control.  Recommended Neoadjuvant Chemotherapy Regimen:  - Anthracycline and taxane-based regimen:  - Doxorubicin (60 mg/m²) or epirubicin (90 mg/m²) combined with cyclophosphamide (600 mg/m²) every 2 weeks for 4 cycles (dose-dense AC) followed by paclitaxel (80 mg/m² weekly for 12 weeks) or docetaxel (100 mg/m² every 3 weeks for 4 cycles).  - Consider adding carboplatin (AUC 5-6 every 3 weeks or AUC 2 weekly) to the taxane phase, as evidence suggests improved pCR rates in TNBC.  **2. Surgical Treatment**  Following neoadjuvant chemotherapy, surgical treatment should be tailored based on the tumor response:  - Breast-conserving surgery (BCS) may be considered if there is a significant reduction in tumor size and clear margins can be achieved.  - If residual disease or extensive involvement remains, bilateral mastectomy is recommended, particularly given the bilateral nature of the disease.  - Sentinel lymph node biopsy (SLNB) should be performed to assess nodal involvement, as the clinical staging is cN0.  **3. Endocrine Treatment**  Endocrine therapy is not indicated as the tumors are ER-negative and PR-negative.  **4. Systemic Treatment**  - If residual disease is present after neoadjuvant chemotherapy, adjuvant capecitabine (1,250 mg/m² orally twice daily on days 1–14 of a 21-day cycle for 6–8 cycles) is recommended, as it has been shown to improve outcomes in TNBC with residual disease.  - No additional systemic therapy is required if pCR is achieved.  **5. Radiation Therapy**  Radiation therapy is indicated post-surgery based on the following:  - Indications:  - If breast-conserving surgery is performed, whole-breast radiation therapy (WBRT) is mandatory.  - If mastectomy is performed, post-mastectomy radiation therapy (PMRT) is recommended for the left side (cT2) and may be considered for the right side (cT1b) depending on pathological findings.  - Regimen:  - WBRT: 50 Gy in 25 fractions over 5 weeks, with a boost of 10–16 Gy to the tumor bed if BCS is performed.  - PMRT: 50 Gy in 25 fractions to the chest wall and regional lymph nodes.  **6. Genetic Testing**  Given the patient's young age (29 years) and significant family history of breast cancer (mother and maternal grandmother), genetic testing for BRCA1/BRCA2 mutations and other hereditary breast cancer syndromes (e.g., PALB2, TP53) is strongly recommended. The results may influence surgical decisions (e.g., prophylactic mastectomy) and systemic therapy options (e.g., use of PARP inhibitors in the metastatic setting).  **Summary of Recommendations**  1. Neoadjuvant chemotherapy: Dose-dense anthracycline and taxane-based regimen, with consideration of adding carboplatin.  2. Surgery: BCS or bilateral mastectomy based on tumor response, with SLNB.  3. Endocrine therapy: Not indicated.  4. Adjuvant systemic therapy: Capecitabine for residual disease.  5. Radiation therapy: WBRT or PMRT based on surgical approach and pathological findings.  6. Genetic testing: Strongly recommended for BRCA1/BRCA2 and other hereditary cancer syndromes. | Here’s a concise, evidence-based plan for a 29-year-old, ECOG 0, premenopausal patient with bilateral triple-negative breast cancer (TNBC) (left cT2N0M0, right cT1bN0M0; G3; ER/PR 0%; HER2 0; very high Ki-67). I’ll lay this out in the order decisions are typically made.  **1) Right now (before any therapy)**   - Fertility & contraception: Urgent referral to onco-fertility for embryo/oocyte cryopreservation. Start GnRH agonist (e.g., goserelin 3.6 mg SC q28 days) 1–2 weeks before chemotherapy and continue through chemo to lower premature ovarian insufficiency risk; this is guideline-endorsed and supported by randomized data. Use effective non-hormonal contraception during systemic therapy. - Genetic testing (fast-tracked before surgery): Yes. Test at least BRCA1/2, PALB2, TP53, CHEK2, ATM, BARD1, RAD51C/D on a multigene panel with genetic counseling. Rationale: age 29, bilateral TNBC, and family history → high pretest probability. Results will influence surgery (e.g., TP53 carriers should avoid breast RT and favor mastectomy) and adjuvant systemic therapy (olaparib if gBRCA+). - Baseline planning & imaging: Place biopsy clips in both tumors before therapy. Breast MRI for extent/response assessment is helpful. Routine metastatic staging is not required in asymptomatic cT1–2N0 disease; some centers consider CT CAP ± bone scan/PET only if symptoms, high nodal burden, or atypical labs. - Cardiac baseline: Echo/MUGA prior to anthracyclines per standard practice. - MDT discussion (breast surgery, medical oncology, radiation oncology, genetics, fertility).   **2) Neoadjuvant therapy (preferred)**  Given left-sided stage II TNBC, the standard of care is neoadjuvant pembrolizumab + chemotherapy, which improves pCR, event-free survival (EFS), and overall survival (OS) vs chemotherapy alone (KEYNOTE-522). This approach also treats the contralateral T1b lesion and can increase breast-conservation options.  Recommended regimen (KEYNOTE-522 backbone):   - Weeks 1–12 (q3 weeks):   - Pembrolizumab 200 mg IV q3 weeks (or 400 mg q6 weeks), plus   - Paclitaxel 80 mg/m² IV weekly × 12, plus   - Carboplatin either AUC 1.5 weekly × 12 *or* AUC 5 q3 weeks × 4. - Weeks 13–24:   - Doxorubicin 60 mg/m² + cyclophosphamide 600 mg/m² q3 weeks × 4 with pembrolizumab (as in KEYNOTE-522). Many centers use dose-dense AC q2 weeks × 4 with G-CSF while giving pembrolizumab q6 weeks; this appears feasible in real-world series but was not the trial default.   Monitoring/notes: check TSH and transaminases; manage immune-related AEs per guidelines; pembrolizumab dosing may be 200 mg q3 w or 400 mg q6 w.  **3) Surgery (after neoadjuvant therapy)**   - Timing: Usually 3–6 weeks after last chemo once counts recover. - Breast procedure: Aim for breast-conserving surgery (BCS) on each side if adequate response/margins and if TP53 negative. If TP53 pathogenic → prefer mastectomy (typically avoiding RT when safe). Discuss nipple-sparing options and symmetry planning. - Axilla: Sentinel lymph node biopsy (SLNB) after NAT on each side (dual tracer; retrieve clipped nodes if any). If SLNB negative → no ALND. If limited nodal disease, tailor ALND vs regional nodal irradiation per institutional standards. - Pathology: Request Residual Cancer Burden (RCB) scoring to guide adjuvant therapy.   **4) Adjuvant systemic therapy (post-op)**   - Pembrolizumab continuation: Continue pembrolizumab monotherapy to complete 1 year total (typically 9 additional q3-week cycles), regardless of pCR. It may be given concurrently with, or after, radiation. - If residual invasive disease (non-pCR):   - gBRCA1/2-positive: Give olaparib 300 mg PO BID for 1 year (OlympiA), which improves iDFS and OS. Many guidelines permit combining or sequencing with adjuvant pembrolizumab; sequence around RT per local practice. Olaparib generally takes priority over capecitabine in gBRCA+ residual disease.   - gBRCA-wild-type: Offer capecitabine 1,000–1,250 mg/m² PO BID days 1–14 q21 days × 6–8 cycles (CREATE-X standard in TNBC-RD). If continuing pembrolizumab, centers commonly give capecitabine sequentially; limited data support concomitant use.   **5) Radiation therapy**  Indications depend on surgery and nodes:   - After BCS (either breast): Whole-breast irradiation (WBI) is mandatory. Use hypofractionation as default:   - 40 Gy in 15 fractions (3 weeks) *or* 26 Gy in 5 fractions (FAST-Forward, 1 week); both are guideline-supported.   - Given age <50, grade 3, and TNBC, add a tumor bed boost (e.g., 10–16 Gy in 4–8 fx). For the left breast, use deep-inspiration breath-hold to minimize heart dose. - After mastectomy:   - pT1–2N0: No PMRT in most cases.   - Any node-positive or close/positive margins or other high-risk features → chest wall + regional nodal irradiation. (If TP53-mutated, try to avoid RT; this can push decision toward mastectomy at the outset.) - Sequencing with pembrolizumab: Can be concurrent or sequential in the adjuvant phase; both approaches are guideline-acceptable.   **6) What if surgery first was chosen?**  Your right tumor is T1bN0; chemotherapy is typically considered for T1b and recommended for ≥T1c in TNBC. Because the left breast is stage II, the plan above (NAT with pembro-chemo) is the best-supported approach overall.  **7) Endocrine therapy**  Not indicated. Tumors are ER/PR 0%.  **Practical “order set” (summary)**   1. Do now: genetics (multigene panel), fertility consult + start goserelin, echo, breast MRI, clip both tumors; MDT. 2. Neoadjuvant (24 weeks total): Pembrolizumab + weekly paclitaxel + carboplatin 12 weeks, then AC × 4 with pembrolizumab. Consider ddAC per center experience. 3. Surgery: BCS if feasible; SLNB bilaterally; mastectomy favored if TP53+. 4. Adjuvant:    - Pembrolizumab to complete 1 year total.    - If residual disease: olaparib 1 year if gBRCA+ (preferred); otherwise capecitabine × 6–8. 5. Radiation: After any BCS, WBI (40/15 *or* 26/5) + boost; consider nodal fields per pathology. Avoid RT if TP53 carrier when possible.   **Why this plan?**   - Pembrolizumab + chemo (KEYNOTE-522): higher pCR and significantly better EFS and OS in stage II–III TNBC, independent of PD-L1 and nodal status. - Capecitabine improves outcomes when residual disease remains after NAT in TNBC. - Olaparib for gBRCA-mutated high-risk HER2-neg early BC gives iDFS and OS benefit. - Hypofractionated RT (40/15 or 26/5) is standard; boost recommended in young, grade 3, TNBC. - Genetics at this age/phenotype is mandatory and alters surgery and adjuvant choices (e.g., avoid RT in TP53). | This patient is a 29-year-old premenopausal woman with synchronous bilateral, node-negative, grade 3, triple-negative breast cancer (TNBC) (ER 0%, PR 0%, HER2 0, high Ki-67), with a strong family history of breast cancer. The following evidence-based management steps are recommended:  **1. Neoadjuvant systemic therapy:**  The American Society of Clinical Oncology (ASCO) recommends that patients with TNBC who have clinically node-positive and/or at least T1c disease should be offered neoadjuvant chemotherapy with an anthracycline- and taxane-containing regimen. Carboplatin may be considered to increase pathologic complete response rates in TNBC. Immune checkpoint inhibitors are not yet standard of care in early-stage TNBC outside clinical trials.  Recommended regimen: Dose-dense doxorubicin/cyclophosphamide (AC) followed by paclitaxel, with optional addition of carboplatin.  **2. Surgical treatment:**  After neoadjuvant chemotherapy, surgery is indicated. Breast-conserving surgery or mastectomy may be considered based on tumor response, size, and patient preference. Sentinel lymph node biopsy should be performed if clinically node-negative.  **3. Endocrine therapy:**  Endocrine therapy is not indicated for this patient, as both tumors are ER and PR negative.  **4. Radiation therapy:**  Postoperative radiation is recommended if breast-conserving surgery is performed. If mastectomy is performed, radiation is indicated for tumors >5 cm, close/positive margins, or if there is nodal involvement at surgery.  **5. Genetic testing:**  Given the young age and strong family history (mother and maternal grandmother with breast cancer), genetic testing for BRCA1/2 and other hereditary breast cancer genes is indicated.  **In summary:**  The patient should receive neoadjuvant anthracycline- and taxane-based chemotherapy (with optional carboplatin), followed by surgery, and postoperative radiation if indicated. Endocrine therapy is not appropriate. Genetic testing is warranted due to her family history and age. |

| **PATIENT CASE 15** |  | |  | |  |  |  |  |
| --- | --- | --- | --- | --- | --- | --- | --- | --- |
|  |  | |  | |  |  |  |  |
| **How difficult or easy was the patient case?** | **very easy** | | **easy** | | **moderate** | **difficult** | **very difficult** | **Score** |
| **Case 15** | **1** | | **2** | | **3** | **4** | **5** |  |
|  |  | |  | |  |  |  |  |
| **The therapy concept is safe.** | **strongly disagree** | | **disagree** | | **neither agree nor disagree** | **agree** | **strongly agree** | **Score** |
| **Therapy Concept 1** | **1** | | **2** | | **3** | **4** | **5** |  |
| **Therapy Concept 2** | **1** | | **2** | | **3** | **4** | **5** |  |
| **Therapy Concept 3** | **1** | | **2** | | **3** | **4** | **5** |  |
|  |  | |  | |  |  |  |  |
| **The therapy concept is in line with the current guidelines.** | **strongly disagree** | | **disagree** | | **neither agree nor disagree** | **agree** | **strongly agree** | **Score** |
| **Therapy Concept 1** | **1** | | **2** | | **3** | **4** | **5** |  |
| **Therapy Concept 2** | **1** | | **2** | | **3** | **4** | **5** |  |
| **Therapy Concept 3** | **1** | | **2** | | **3** | **4** | **5** |  |
|  |  | |  | |  |  |  |  |
| **The therapy concept is medically adequate.** | **strongly disagree** | | **disagree** | | **neither agree nor disagree** | **agree** | **strongly agree** | **Score** |
| **Therapy Concept 1** | **1** | | **2** | | **3** | **4** | **5** |  |
| **Therapy Concept 2** | **1** | | **2** | | **3** | **4** | **5** |  |
| **Therapy Concept 3** | **1** | | **2** | | **3** | **4** | **5** |  |
|  |  | |  | |  |  |  |  |
| **The therapy concept is complete.** | **strongly disagree** | | **disagree** | | **neither agree nor disagree** | **agree** | **strongly agree** | **Score** |
| **Therapy Concept 1** | **1** | | **2** | | **3** | **4** | **5** |  |
| **Therapy Concept 2** | **1** | | **2** | | **3** | **4** | **5** |  |
| **Therapy Concept 3** | **1** | | **2** | | **3** | **4** | **5** |  |
|  |  | |  | |  |  |  |  |
| **The therapy concept is of high quality.** | **strongly disagree** | | **disagree** | | **neither agree nor disagree** | **agree** | **strongly agree** | **Score** |
| **Therapy Concept 1** | **1** | | **2** | | **3** | **4** | **5** |  |
| **Therapy Concept 2** | **1** | | **2** | | **3** | **4** | **5** |  |
| **Therapy Concept 3** | **1** | | **2** | | **3** | **4** | **5** |  |
|  |  | |  | |  |  |  |  |
| **The reasoning behind the treatment decision is logical.** | **strongly disagree** | | **disagree** | | **neither agree nor disagree** | **agree** | **strongly agree** | **Score** |
| **Therapy Concept 1** | **1** | | **2** | | **3** | **4** | **5** |  |
| **Therapy Concept 2** | **1** | | **2** | | **3** | **4** | **5** |  |
| **Therapy Concept 3** | **1** | | **2** | | **3** | **4** | **5** |  |
|  |  | |  | |  |  |  |  |
| **Please rank the therapy concept according to your preference.** | | **Ranking (1^st^, 2^nd^, 3^rd^)** | |  |  |  |  |  |
| **Therapy Concept 1** | |  | |  |  |  |  |  |
| **Therapy Concept 2** | |  | |  |  |  |  |  |
| **Therapy Concept 3** | |  | |  |  |  |  |  |

**Patient 16**

How should the following breast cancer patient be treated based on the most relevant and current international literature?

Patient information:

Age: 35

Menopause status: premenopausal

ECOG: 0

Previous illness: Paranoid schizophrenia (under stable condition with current olanzapine medication)

Previous surgical treatment: None

Birth history: No prior birth

Oncological family history: Maternal grandmother with endometrial cancer at age of 60, paternal uncle with rectum carcinoma at the age of 50

The patient did not undergo surgery so far.

Following the surgical biopsy and staging, the following information about the tumor is available:

TNM: cT2pN+pM1 (HEP)

Histological classification: NST

Grading: G3

Estrogen receptor (ER): 1%

Progesterone receptor (PR): 2%

Her2-status: Negative (IHC 1+)

Ki-67-proliferation-index: 80%

Please provide a step-by-step treatment recommendation with regard to the necessity of neoadjuvant treatment, present or future surgical treatment, endocrine treatment, systemic treatment and radiation therapy taking the given patient information into consideration.

If endocrine treatment is advisable, please provide a suitable treatment regimen.

If systemic treatment is advisable, please provide a suitable treatment regimen.

If radiation therapy is advisable, please provide a suitable treatment regimen.

Based on the oncological family history, please state whether genetic testing should be performed or not.

| **Patient 16 Treatment Concepts** |  |  |
| --- | --- | --- |
| **Concept 1** | **Concept 2** | **Concept 3** |
| **Step-by-Step Treatment Recommendation**  **1. Neoadjuvant Treatment**  Given the tumor's characteristics (cT2, G3, triple-negative breast cancer [TNBC], high Ki-67 index, and metastatic disease to the liver [M1]), neoadjuvant systemic therapy is not indicated as the primary goal in metastatic breast cancer is systemic disease control rather than local tumor downstaging. Instead, systemic therapy should be initiated promptly to address the metastatic burden.  **2. Systemic Treatment**  The patient has triple-negative breast cancer (TNBC) with liver metastases. The recommended systemic treatment regimen is as follows:  - First-line chemotherapy: A platinum-based regimen (e.g., carboplatin combined with paclitaxel) is preferred due to the aggressive nature of TNBC and its potential sensitivity to platinum agents.  - Immunotherapy: If the tumor expresses PD-L1 (≥1% on immune cells, as determined by an FDA-approved assay), the addition of atezolizumab to nab-paclitaxel is recommended. If PD-L1 status is unknown, testing should be performed.  - Duration: Chemotherapy should be continued for 4-6 months or until maximum response, followed by maintenance therapy if applicable.  **3. Surgical Treatment**  Surgery is not the primary treatment modality in metastatic breast cancer (M1). However, surgical resection of the primary tumor may be considered in select cases for symptom control or if systemic disease becomes well-controlled. This decision should be revisited after systemic therapy.  **4. Endocrine Treatment**  Endocrine therapy is not indicated as the tumor is hormone receptor–negative (ER 1%, PR 2%).  **5. Radiation Therapy**  Radiation therapy may be considered for palliative purposes, particularly if the patient develops symptomatic metastases (e.g., bone pain, brain metastases) or if local control of the primary tumor becomes necessary. Whole-breast or chest wall radiation is not routinely indicated in the metastatic setting unless for symptom management.  **6. Genetic Testing**  Given the patient's young age (35 years), triple-negative breast cancer, and a family history of endometrial cancer in a first-degree relative (maternal grandmother), genetic testing for BRCA1/2 mutations and other hereditary cancer syndromes (e.g., Lynch syndrome) is strongly recommended. Identification of a BRCA mutation would influence treatment decisions, as PARP inhibitors (e.g., olaparib) could be considered.  **Summary of Treatment Plan**  1. Systemic Therapy: Initiate carboplatin + paclitaxel. Add atezolizumab if PD-L1 is positive.  2. Surgery: Reassess after systemic therapy for potential palliative resection.  3. Radiation Therapy: Consider for palliation of symptoms if needed.  4. Genetic Testing: Perform BRCA1/2 and Lynch syndrome testing. | Here’s a practical, guideline-concordant plan for this 35-year-old, premenopausal woman with de-novo metastatic breast cancer to the liver (cT2 pN+ pM1[hepatic]), high grade, ER-low (ER 1%/PR 2%), HER2 IHC 1+ (i.e., HER2-low), Ki-67 80%, ECOG 0.  **1) First things first (do now)**   - Confirm predictive biomarkers on a metastatic site (e.g., liver biopsy):   - PD-L1 CPS by 22C3 (companion diagnostic for pembrolizumab; threshold CPS ≥ 10).   - Reconfirm ER/PR/HER2 (to verify HER2-low = IHC 1+ or 2+/ISH–, which unlocks trastuzumab deruxtecan later). - Germline genetics (see §7): urgent BRCA1/2 testing (and panel; details below) because this changes first-line options. - Baseline before immunotherapy/ADCs: CBC/CMP, hepatitis B/C, HIV; CT chest/abdomen/pelvis ± MRI liver; pregnancy test; discuss contraception.   Rationale: For de-novo stage IV, treatment is systemic first; surgery/radiation are not curative and are reserved for symptoms or strict oligometastatic scenarios.  **2) Should we use “neoadjuvant” therapy or operate first?**  No. In de-novo stage IV disease, early surgery or routine breast/axillary radiation does not improve overall survival vs systemic therapy alone (ECOG-ACRIN E2108). Consider local therapy only for palliation (bleeding, ulceration, pain) or highly selected oligometastatic settings after systemic response.  **3) Is endocrine therapy appropriate?**  With ER 1%/PR 2%, this is ER-low. Current ASCO/CAP and ESMO/ABC guidance notes limited/uncertain benefit from endocrine therapy; ER-low metastatic cancers often behave like triple-negative. Therefore do not start with endocrine therapy; prioritize systemic (chemo-/immuno-/targeted) therapy.  **4) First-line systemic treatment (choose based on PD-L1 and germline BRCA)**  A) If PD-L1 CPS ≥ 10 (preferred):  Pembrolizumab + chemotherapy (KEYNOTE-355 standard of care; OS benefit). Practical backbones:   - Pembrolizumab 200 mg IV q3 weeks (or 400 mg q6 weeks) + nab-paclitaxel 100 mg/m² IV d1,8,15 q28 *Why nab-paclitaxel?* avoids routine steroid premedication—useful with a history of schizophrenia where steroids may destabilize mood. – or – - Pembrolizumab + paclitaxel 90 mg/m² IV d1,8,15 q28 – or – - Pembrolizumab + gemcitabine 1000 mg/m² + carboplatin AUC 2 IV d1,8 q21   Continue pembrolizumab to 2 years if benefiting; continue chemo until best response/toxicity.  B) If PD-L1 CPS < 10 and germline BRCA1/2 pathogenic variant present:  A PARP inhibitor is appropriate first-line in HER2-negative mBC:   - Olaparib 300 mg PO BID (preferred given strongest dataset) – improved PFS vs physician’s-choice chemo in OlympiAD; OS trends with long follow-up. – or – - Talazoparib 1 mg PO daily (EMBRACA; superior PFS vs chemo).   C) If PD-L1 CPS < 10 and no germline BRCA:  Start single-agent or doublet chemotherapy (taxane or platinum-based). Practical choices:   - Carboplatin AUC 5–6 q3 weeks ± gemcitabine 1000 mg/m² d1,8 q21 - Weekly paclitaxel 80–90 mg/m² d1,8,15 q28 (Select based on comorbidities, patient preference, and toxicity profile.) KEYNOTE-355 allowed these backbones; platinum is reasonable in TNBC.   Supportive notes: With olanzapine on board, you already have an excellent antiemetic; watch for sedation. Prefer nab-paclitaxel up front if immunotherapy-eligible to limit steroid exposure.  **5) Second line and beyond (planned sequencing)**  Because the tumor is HER2-low (IHC 1+), plan to use trastuzumab deruxtecan (T-DXd) after at least one prior chemotherapy for mBC:   - T-DXd 5.4 mg/kg IV q3 weeks until progression/toxicity.   - Proven PFS/OS benefit in HER2-low mBC (DESTINY-Breast04); FDA/EMA label requires ≥1 prior chemo in the metastatic (or early relapse) setting. Monitor for ILD/pneumonitis—hold and start steroids early if suspected.   After T-DXd (or if T-DXd not appropriate):   - Sacituzumab govitecan (SG) 10 mg/kg IV d1,8 q21 with antiemetic/diarrhea prophylaxis; improves OS vs single-agent chemo in mTNBC (ASCENT). *Regulatory note:* in the EU, SG is approved after ≥2 prior systemic therapies for mTNBC; in some regions it may be used earlier—follow local rules.   Later options/clinical trials: eribulin, capecitabine, vinorelbine, platinum rechallenge; consider trials (including novel ADCs).  **6) Role of radiation and surgery in this case**   - Breast/axilla: Reserve RT for symptom control (bleeding, pain, fungation) or impending complications. Hypofractionated options: 30 Gy/10 fx or 20 Gy/5 fx for palliation; 40 Gy/15 fx for more durable local control if needed. E2108 shows no OS benefit from routine surgery/RT of the intact primary in de-novo stage IV. - Liver lesions: If disease proves oligometastatic and well-controlled systemically, a tumor board can consider SBRT (typical ranges 45–60 Gy in 3–5 fx, respecting liver constraints) or ablation/resection. Evidence suggests good local control with low toxicity, but survival benefit remains unproven; use selectively.   **7) Genetic testing (germline) — should we do it?**  Yes. The patient is 35 and has (functionally) TNBC. Current ASCO/SSO and NCCN guidance support offering BRCA1/2 testing to all patients ≤65 with a new breast cancer, and specifically to all TNBC—both for familial risk and because PARP inhibitors alter systemic therapy. A multigene panel is reasonable; prioritize BRCA1, BRCA2, PALB2, and consider TP53 given young age (especially if any suggestive family history), typically via genetics counseling.  *Note on family history:* The grandmother’s endometrial cancer and an uncle’s colorectal cancer on different sides do not by themselves prove a Lynch syndrome pattern; panel testing led by genetics can address this.  **8) Extra practicalities**   - Contraception: Strict effective contraception during and for the label-specified washout after systemic therapy (pembrolizumab/ADCs are teratogenic/contraindicated). - Psychiatric comorbidity: Avoid or minimize corticosteroids when possible (favor nab-paclitaxel over solvent paclitaxel); coordinate closely with psychiatry. - Monitoring for T-DXd: baseline & periodic chest imaging; educate on early cough/dyspnea; treat suspected ILD promptly with steroids. - Immunotherapy safety: With liver metastases, watch LFTs for immune-related hepatitis; manage per guidelines. KEYNOTE-355 PROs show maintained quality of life with pembro-chemo.   **Quick “if-then” summary you can implement**   1. Send PD-L1 CPS, confirm HER2-low, order germline BRCA1/2 (±PALB2, TP53) now. 2. If CPS ≥ 10: start pembrolizumab + nab-paclitaxel (or + paclitaxel or + gem/carbo). 3. If CPS < 10 & gBRCA+: start olaparib (or talazoparib). 4. If CPS < 10 & gBRCA–: start taxane or platinum-based chemo (e.g., weekly paclitaxel or gem/carbo). 5. At progression: use T-DXd (HER2-low) → then sacituzumab govitecan (per EU label typically ≥3L). 6. Use surgery/RT only for symptoms or carefully selected oligometastatic indications; not for routine control of the primary.   **Key sources**   - KEYNOTE-355: pembrolizumab + chemo improves OS in CPS ≥ 10 mTNBC. - ASCO/CAP ER-low guidance: limited benefit from endocrine therapy when ER 1–10%. - DESTINY-Breast04 / labels: T-DXd benefits HER2-low after ≥1 chemo. - ASCENT: sacituzumab govitecan improves OS vs chemo in pretreated mTNBC (EU: ≥2 prior lines). - E2108: locoregional therapy of the primary does not improve OS in de-novo stage IV. - ASCO/SSO 2024 genetic testing: offer BRCA1/2 ≤65 y; TNBC qualifies. | This 35-year-old premenopausal woman with metastatic (stage IV) hormone receptor–low (ER 1%, PR 2%), HER2-negative, high-grade (G3), high-proliferation (Ki-67 80%) breast cancer and hepatic metastasis should be managed with systemic therapy as first-line treatment. Surgery and radiation are generally reserved for palliation or local control in metastatic disease, not as curative modalities.  Neoadjuvant treatment is not indicated in metastatic (M1) breast cancer, as systemic therapy is the mainstay and local therapy is reserved for symptom control or specific indications.  **Systemic treatment:**  The American Society of Clinical Oncology recommends that patients with hormone receptor–positive, HER2-negative metastatic breast cancer receive endocrine therapy as initial treatment, except in cases of rapidly progressive visceral disease or low hormone receptor expression, where chemotherapy is preferred. Given the very low ER/PR expression and aggressive tumor biology (G3, high Ki-67, visceral metastasis), chemotherapy is the preferred first-line regimen. Anthracycline- and taxane-based regimens are standard; dose and schedule should be individualized based on tolerability and comorbidities.  **Endocrine therapy:**  With ER/PR expression <10%, benefit from endocrine therapy is limited, but may be considered after chemotherapy if disease stabilizes and receptor positivity is confirmed. In premenopausal women, ovarian suppression plus tamoxifen or aromatase inhibitor is standard if endocrine therapy is used. However, in this case, endocrine therapy is not advisable as first-line treatment due to low receptor expression and visceral crisis.  **Radiation therapy:**  Indicated only for palliation of symptomatic metastases (e.g., bone pain, brain metastases) or local control if needed. Routine radiation is not recommended.  **Surgical treatment:**  Not indicated for metastatic disease except for palliation (e.g., bleeding, ulceration).  **Genetic testing:**  Given the young age, triple-negative-like biology, and family history of endometrial and rectal cancer, germline BRCA1/2 testing is recommended. The American Society of Clinical Oncology advises genetic testing for patients with early-onset breast cancer and/or suggestive family history.  **In summary:**  Chemotherapy is the first-line treatment, with consideration of palliative local therapies as needed, and genetic testing for BRCA1/2 mutations should be performed. Endocrine therapy may be considered in later lines if receptor positivity is confirmed and disease is indolent. |

| **PATIENT CASE 16** |  | |  | |  |  |  |  |
| --- | --- | --- | --- | --- | --- | --- | --- | --- |
|  |  | |  | |  |  |  |  |
| **How difficult or easy was the patient case?** | **very easy** | | **easy** | | **moderate** | **difficult** | **very difficult** | **Score** |
| **Case 16** | **1** | | **2** | | **3** | **4** | **5** |  |
|  |  | |  | |  |  |  |  |
| **The therapy concept is safe.** | **strongly disagree** | | **disagree** | | **neither agree nor disagree** | **agree** | **strongly agree** | **Score** |
| **Therapy Concept 1** | **1** | | **2** | | **3** | **4** | **5** |  |
| **Therapy Concept 2** | **1** | | **2** | | **3** | **4** | **5** |  |
| **Therapy Concept 3** | **1** | | **2** | | **3** | **4** | **5** |  |
|  |  | |  | |  |  |  |  |
| **The therapy concept is in line with the current guidelines.** | **strongly disagree** | | **disagree** | | **neither agree nor disagree** | **agree** | **strongly agree** | **Score** |
| **Therapy Concept 1** | **1** | | **2** | | **3** | **4** | **5** |  |
| **Therapy Concept 2** | **1** | | **2** | | **3** | **4** | **5** |  |
| **Therapy Concept 3** | **1** | | **2** | | **3** | **4** | **5** |  |
|  |  | |  | |  |  |  |  |
| **The therapy concept is medically adequate.** | **strongly disagree** | | **disagree** | | **neither agree nor disagree** | **agree** | **strongly agree** | **Score** |
| **Therapy Concept 1** | **1** | | **2** | | **3** | **4** | **5** |  |
| **Therapy Concept 2** | **1** | | **2** | | **3** | **4** | **5** |  |
| **Therapy Concept 3** | **1** | | **2** | | **3** | **4** | **5** |  |
|  |  | |  | |  |  |  |  |
| **The therapy concept is complete.** | **strongly disagree** | | **disagree** | | **neither agree nor disagree** | **agree** | **strongly agree** | **Score** |
| **Therapy Concept 1** | **1** | | **2** | | **3** | **4** | **5** |  |
| **Therapy Concept 2** | **1** | | **2** | | **3** | **4** | **5** |  |
| **Therapy Concept 3** | **1** | | **2** | | **3** | **4** | **5** |  |
|  |  | |  | |  |  |  |  |
| **The therapy concept is of high quality.** | **strongly disagree** | | **disagree** | | **neither agree nor disagree** | **agree** | **strongly agree** | **Score** |
| **Therapy Concept 1** | **1** | | **2** | | **3** | **4** | **5** |  |
| **Therapy Concept 2** | **1** | | **2** | | **3** | **4** | **5** |  |
| **Therapy Concept 3** | **1** | | **2** | | **3** | **4** | **5** |  |
|  |  | |  | |  |  |  |  |
| **The reasoning behind the treatment decision is logical.** | **strongly disagree** | | **disagree** | | **neither agree nor disagree** | **agree** | **strongly agree** | **Score** |
| **Therapy Concept 1** | **1** | | **2** | | **3** | **4** | **5** |  |
| **Therapy Concept 2** | **1** | | **2** | | **3** | **4** | **5** |  |
| **Therapy Concept 3** | **1** | | **2** | | **3** | **4** | **5** |  |
|  |  | |  | |  |  |  |  |
| **Please rank the therapy concept according to your preference.** | | **Ranking (1^st^, 2^nd^, 3^rd^)** | |  |  |  |  |  |
| **Therapy Concept 1** | |  | |  |  |  |  |  |
| **Therapy Concept 2** | |  | |  |  |  |  |  |
| **Therapy Concept 3** | |  | |  |  |  |  |  |

**Patient 17**

How should the following ductal carcinoma in situ patient be treated based on the most relevant and current international literature?

Patient information:

Age: 70

Menopause status: postmenopausal

ECOG: 2

Previous illness: Arterial hypertension (with AT II antagonist medication)

Previous surgical treatment: Vaginal hysterectomy with bilateral adnexectomy for uterine prolapse at the age of 55, transcatheter aortic valve implantation due to aortic valve stenosis at the age of 69

Birth history: 2 vaginal births at the age of 16 and 20

Oncological family history: 1 sister with peritoneal cancer at the age of 60, maternal grandmother with ovarian cancer at the age of 65

The patient underwent the following surgical treatment: Right-sided mastectomy

Following the surgical treatment, the following TNM-classification, resection margin, histological classification, immunohistology were identified:

TNM: pTis (size of the lesion 4.3 cm)

Resection margin: R0, minimal resection margin of 10 mm

Estrogen receptor (ER): 95%

Progesterone receptor (PR): 90%

Please provide a step-by-step treatment recommendation with regard to further surgical treatment, endocrine treatment, systemic treatment and radiation therapy taking the given patient information in consideration.

If endocrine treatment is advisable, please provide a suitable treatment regimen.

If systemic treatment is advisable, please provide a suitable treatment regimen.

If radiation therapy is advisable, please provide a suitable treatment regimen.

Based on the oncological family history, please state whether genetic testing should be performed or not.

| **Patient 17 Treatment Concepts** |  |  |
| --- | --- | --- |
| **Concept 1** | **Concept 2** | **Concept 3** |
| **Step-by-Step Treatment Recommendation for the Patient with Ductal Carcinoma In Situ (DCIS)**  **1. Further Surgical Treatment**  - No further surgical treatment is required. The patient has already undergone a right-sided mastectomy with clear resection margins (R0) and a minimal margin of 10 mm, which is considered adequate for DCIS.  **2. Endocrine Treatment**  - Endocrine therapy is recommended due to the high expression of estrogen (ER: 95%) and progesterone receptors (PR: 90%), indicating hormone receptor-positive disease.  - Recommended Regimen:  - Drug: Aromatase inhibitor (e.g., anastrozole 1 mg daily or letrozole 2.5 mg daily).  - Duration: 5 years.  - Rationale: Aromatase inhibitors are preferred in postmenopausal women to reduce the risk of recurrence. Tamoxifen could be considered as an alternative if aromatase inhibitors are contraindicated.  **3. Systemic Treatment**  - Systemic chemotherapy is not indicated for DCIS, as it is a non-invasive lesion (pTis) and there is no evidence of invasive disease or nodal involvement.  **4. Radiation Therapy**  - Radiation therapy is not indicated in this case because the patient has undergone a mastectomy. Post-mastectomy radiation therapy is generally reserved for invasive breast cancer with high-risk features, such as large tumor size or nodal involvement, which are absent here.  **5. Genetic Testing**  - Genetic testing is recommended based on the patient’s oncological family history:  - A sister with peritoneal cancer at age 60 and a maternal grandmother with ovarian cancer at age 65 suggest a possible hereditary cancer syndrome, such as BRCA1/BRCA2 mutation.  - Recommended Testing: Comprehensive germline genetic testing for BRCA1, BRCA2, and other relevant genes (e.g., PALB2, RAD51C/D) associated with hereditary breast and ovarian cancer syndrome.  **Summary of Recommendations**  - Surgical Treatment: No further surgery required.  - Endocrine Therapy: Aromatase inhibitor for 5 years.  - Systemic Therapy: Not indicated.  - Radiation Therapy: Not indicated.  - Genetic Testing: Recommended due to family history of ovarian and peritoneal cancer.  This treatment plan is tailored to the patient’s postmenopausal status, ECOG performance status, comorbidities, and the non-invasive nature of the DCIS. | Here’s a concise, guideline-concordant plan tailored to this 70-year-old, ECOG 2, postmenopausal patient with pure ER+/PR+ DCIS treated by right mastectomy (pTis, R0, 10-mm margins).  **1) Further surgical treatment**   - No additional breast or axillary surgery is indicated. After a mastectomy with final pathology confirming pure DCIS (pTis) and clear margins, there’s no role for re-excision or completion axillary surgery. SLNB is typically done (or omitted) at the time of mastectomy in DCIS; if not performed and final pathology is DCIS only, no further axillary surgery is required. - Reconstruction remains an elective option based on preference and fitness; it does not alter oncologic outcomes.   **2) Endocrine (anti-estrogen) therapy**   - Recommendation: *Do not start routine endocrine therapy after a unilateral mastectomy for pure DCIS.* After mastectomy, ipsilateral events are rare, and randomized/observational data show little to no benefit from endocrine therapy in this setting, while adding toxicity—particularly relevant at age 70 with a TAVI history (raised VTE risk with tamoxifen; AIs cause bone loss). - If the patient strongly wishes to reduce contralateral breast cancer risk after shared decision-making, discuss optional chemoprevention:   - Tamoxifen 20 mg daily for 5 years (standard), or low-dose tamoxifen 5 mg daily for 3 years (“Baby-TAM”; off-label but supported by RCT showing durable event reduction with fewer side effects). However, tamoxifen increases VTE risk in older women; with ECOG 2 and prior valve disease, risk–benefit is unfavorable for most.   - Anastrozole 1 mg daily for 5 years is an alternative for postmenopausal women who cannot take tamoxifen (IBIS-II DCIS showed similar efficacy to tamoxifen after local excision), but AIs accelerate bone loss—another reason to avoid if the baseline fracture risk is high. If chosen, add bone health measures (baseline DXA, calcium/vitamin D, fall-prevention; consider antiresorptives per risk).   Bottom line for this patient: given age 70, ECOG 2, TAVI and the very low post-mastectomy event risk, omit endocrine therapy.  **3) Systemic (chemotherapy, HER2-directed) therapy**   - Not indicated. DCIS has no role for chemotherapy or anti-HER2 agents in the absence of invasion. (Standard across guidelines.)   **4) Radiation therapy**   - Not indicated after mastectomy for pure DCIS with negative margins. Post-mastectomy radiation is reserved for invasive disease and select high-risk scenarios; it is generally not used for DCIS with clear margins after mastectomy.   **5) Genetics**   - Recommend germline genetic counseling and testing. A first-degree relative (sister) with *peritoneal cancer* at 60 and a second-degree relative (maternal grandmother) with ovarian cancer at 65 meet contemporary criteria to offer testing for hereditary breast–ovarian cancer susceptibility (e.g., BRCA1/2, PALB2; often via a multigene panel). Even though this patient already had BSO, results importantly guide contralateral surveillance and cascade testing for relatives.   - Typical panel: BRCA1, BRCA2, PALB2, CHEK2, ATM, RAD51C, RAD51D, BRIP1 (panel content varies by lab and guidelines).   **6) Follow-up & survivorship (practical plan)**   - Clinical visits: every 6–12 months for 5 years, then annually (may be streamlined given age/comorbidity). - Imaging:   - No routine imaging of the mastectomy side/reconstructed chest wall.   - Annual mammography of the contralateral breast. - If endocrine therapy is elected (not recommended here): manage risks proactively—VTE precautions for tamoxifen; bone health plan and DXA for AIs. - General health: optimise blood pressure and mobility; vaccinate; fall-prevention; address vasomotor, sexual, and psychosocial concerns as needed.   **Why this plan?**   - After mastectomy for DCIS, local recurrence risk is extremely low, and studies/guidelines consistently show no routine role for RT or endocrine therapy in this specific setting; endocrine therapy’s marginal contralateral benefit is often outweighed by toxicity in older, comorbid patients. - Family history with ovarian/peritoneal cancers triggers genetic testing offers under NCCN/ESMO frameworks, chiefly to inform relatives and personalize the patient’s ongoing surveillance. | For a 70-year-old postmenopausal woman with DCIS (pTis, 4.3 cm, ER 95%, PR 90%) who has undergone right-sided mastectomy with clear margins (R0, 10 mm), the following evidence-based recommendations apply:  **Further surgical treatment:**  No additional breast surgery is indicated after total mastectomy with clear margins for DCIS. Axillary staging is not routinely recommended for pure DCIS unless invasive disease is suspected, which is not the case here.  **Endocrine treatment:**  Endocrine therapy (e.g., tamoxifen or aromatase inhibitors) is primarily recommended for risk reduction of contralateral breast cancer in patients with hormone receptor-positive DCIS who have undergone breast-conserving surgery, not mastectomy. The benefit after mastectomy is minimal, as the risk of ipsilateral recurrence is extremely low. For this patient, endocrine therapy is not routinely recommended. If there are significant risk factors for contralateral breast cancer and the patient is highly motivated, a shared decision-making approach may be considered, but routine use is not supported by current literature.  **Systemic treatment:**  No systemic chemotherapy is indicated for pure DCIS, as it is a non-invasive lesion and there is no evidence supporting chemotherapy in this setting.  **Radiation therapy:**  No adjuvant radiation therapy is indicated after mastectomy for DCIS, as the entire breast tissue has been removed and local recurrence risk is extremely low.  **Genetic testing:**  Given the family history (sister with peritoneal cancer, grandmother with ovarian cancer), there is a possible hereditary cancer syndrome (e.g., BRCA mutation). Genetic counseling and testing should be considered for this patient, as per current risk assessment strategies.  **In summary:**  • No further surgery, radiation, or systemic therapy is indicated.  • Endocrine therapy is not routinely recommended after mastectomy for DCIS.  • Genetic testing should be considered due to family history.  These recommendations are consistent with current international literature and consensus. |

| **PATIENT CASE 17** |  | |  | |  |  |  |  |
| --- | --- | --- | --- | --- | --- | --- | --- | --- |
|  |  | |  | |  |  |  |  |
| **How difficult or easy was the patient case?** | **very easy** | | **easy** | | **moderate** | **difficult** | **very difficult** | **Score** |
| **Case 17** | **1** | | **2** | | **3** | **4** | **5** |  |
|  |  | |  | |  |  |  |  |
| **The therapy concept is safe.** | **strongly disagree** | | **disagree** | | **neither agree nor disagree** | **agree** | **strongly agree** | **Score** |
| **Therapy Concept 1** | **1** | | **2** | | **3** | **4** | **5** |  |
| **Therapy Concept 2** | **1** | | **2** | | **3** | **4** | **5** |  |
| **Therapy Concept 3** | **1** | | **2** | | **3** | **4** | **5** |  |
|  |  | |  | |  |  |  |  |
| **The therapy concept is in line with the current guidelines.** | **strongly disagree** | | **disagree** | | **neither agree nor disagree** | **agree** | **strongly agree** | **Score** |
| **Therapy Concept 1** | **1** | | **2** | | **3** | **4** | **5** |  |
| **Therapy Concept 2** | **1** | | **2** | | **3** | **4** | **5** |  |
| **Therapy Concept 3** | **1** | | **2** | | **3** | **4** | **5** |  |
|  |  | |  | |  |  |  |  |
| **The therapy concept is medically adequate.** | **strongly disagree** | | **disagree** | | **neither agree nor disagree** | **agree** | **strongly agree** | **Score** |
| **Therapy Concept 1** | **1** | | **2** | | **3** | **4** | **5** |  |
| **Therapy Concept 2** | **1** | | **2** | | **3** | **4** | **5** |  |
| **Therapy Concept 3** | **1** | | **2** | | **3** | **4** | **5** |  |
|  |  | |  | |  |  |  |  |
| **The therapy concept is complete.** | **strongly disagree** | | **disagree** | | **neither agree nor disagree** | **agree** | **strongly agree** | **Score** |
| **Therapy Concept 1** | **1** | | **2** | | **3** | **4** | **5** |  |
| **Therapy Concept 2** | **1** | | **2** | | **3** | **4** | **5** |  |
| **Therapy Concept 3** | **1** | | **2** | | **3** | **4** | **5** |  |
|  |  | |  | |  |  |  |  |
| **The therapy concept is of high quality.** | **strongly disagree** | | **disagree** | | **neither agree nor disagree** | **agree** | **strongly agree** | **Score** |
| **Therapy Concept 1** | **1** | | **2** | | **3** | **4** | **5** |  |
| **Therapy Concept 2** | **1** | | **2** | | **3** | **4** | **5** |  |
| **Therapy Concept 3** | **1** | | **2** | | **3** | **4** | **5** |  |
|  |  | |  | |  |  |  |  |
| **The reasoning behind the treatment decision is logical.** | **strongly disagree** | | **disagree** | | **neither agree nor disagree** | **agree** | **strongly agree** | **Score** |
| **Therapy Concept 1** | **1** | | **2** | | **3** | **4** | **5** |  |
| **Therapy Concept 2** | **1** | | **2** | | **3** | **4** | **5** |  |
| **Therapy Concept 3** | **1** | | **2** | | **3** | **4** | **5** |  |
|  |  | |  | |  |  |  |  |
| **Please rank the therapy concept according to your preference.** | | **Ranking (1^st^, 2^nd^, 3^rd^)** | |  |  |  |  |  |
| **Therapy Concept 1** | |  | |  |  |  |  |  |
| **Therapy Concept 2** | |  | |  |  |  |  |  |
| **Therapy Concept 3** | |  | |  |  |  |  |  |

**Patient 18**

How should the following ductal carcinoma in situ patient be treated based on the most relevant and current international literature?

Patient information:

Age: 38

Menopause status: premenopausal

ECOG: 0

Previous illness: AV-node re-entry tachycardia (with beta-blocker medication)

Previous surgical treatment: None

Birth history: 1 cesarean at the age of 36

Oncological family history: Paternal grandfather with prostate cancer at the age of 65, mother with chronic myeloid leukemia at the age of 70

The patient underwent the following surgical treatment: Breast-conserving tumorectomy on the right and left side

Following the surgical treatment, the following TNM-classification, resection margin, histological classification and immunohistology were identified:

TNM: pTis on left and right side (size of the lesions: 2.3 cm on left and 3.2 cm on right side)

Resection margin: R0, minimal resection margin of 4 mm on left side and 5 mm on right side

Estrogen receptor (ER): 100% on left and right side

Progesterone receptor (PR): 100% on left and right side

Please provide a step-by-step treatment recommendation with regard to further surgical treatment, endocrine treatment, systemic treatment and radiation therapy taking the given patient information in consideration.

If endocrine treatment is advisable, please provide a suitable treatment regimen.

If systemic treatment is advisable, please provide a suitable treatment regimen.

If radiation therapy is advisable, please provide a suitable treatment regimen.

Based on the oncological family history, please state whether genetic testing should be performed or not.

| **Patient 18 Treatment Concepts** |  |  |
| --- | --- | --- |
| **Concept 1** | **Concept 2** | **Concept 3** |
| **Step-by-Step Treatment Recommendation for the Patient with Bilateral Ductal Carcinoma In Situ (DCIS)**  **1. Further Surgical Treatment**  - No further surgical treatment is required as the patient has undergone breast-conserving surgery (tumorectomy) with clear resection margins (R0) and a minimum margin of 4 mm on the left and 5 mm on the right side. Current guidelines consider these margins sufficient for DCIS.  **2. Radiation Therapy**  -Recommendation: Adjuvant whole-breast radiation therapy (WBRT) is recommended for both breasts to reduce the risk of local recurrence.  Regimen:  - Standard WBRT: 50 Gy in 25 fractions over 5 weeks, or  - Hypofractionated WBRT: 40–42.5 Gy in 15–16 fractions over 3 weeks, which is equally effective and more convenient.  - A boost dose of 10–16 Gy to the tumor bed may be considered, especially in younger patients (<50 years) due to their higher risk of recurrence.  **3. Endocrine Treatment**  - Recommendation: Endocrine therapy is advisable given the strong estrogen receptor (ER) and progesterone receptor (PR) positivity (100% on both sides). This therapy reduces the risk of recurrence in both the ipsilateral and contralateral breasts.  - Regimen:  - Tamoxifen: 20 mg daily for 5 years is the preferred option for premenopausal women.  - Alternatively, ovarian suppression with a gonadotropin-releasing hormone (GnRH) agonist (e.g., goserelin) combined with an aromatase inhibitor (e.g., anastrozole or letrozole) may be considered, but this is less commonly used in DCIS.  **4. Systemic Treatment**  - Recommendation: Systemic chemotherapy is not indicated for DCIS, as it is a non-invasive condition with no evidence of metastatic potential.  **5. Genetic Testing**  - Recommendation: Genetic testing is not routinely indicated based on the provided family history. However, the patient’s young age (38 years) at diagnosis and bilateral presentation of DCIS may warrant consideration of genetic counseling and testing for hereditary breast cancer syndromes (e.g., BRCA1/BRCA2 mutations).  - Testing is particularly relevant if there is additional family history of breast or ovarian cancer, or if the patient expresses concern about hereditary risk.  **Summary of Recommendations**  1. No further surgical treatment is needed.  2. Adjuvant whole-breast radiation therapy is recommended, with a possible boost to the tumor bed.  3. Endocrine therapy with tamoxifen (20 mg daily for 5 years) is advised.  4. Systemic chemotherapy is not indicated.  5. Genetic counseling and testing may be considered due to the patient’s age and bilateral DCIS, though the family history alone does not strongly suggest hereditary breast cancer. | Here’s a concise, guideline-concordant plan for this 38-year-old, premenopausal woman with bilateral ER/PR-positive DCIS after breast-conserving surgery (clear margins 4–5 mm; pTis both breasts).  **1) Further surgery / axilla**   - No further breast surgery: Margins are ≥2 mm, which meets the SSO-ASTRO-ASCO consensus for DCIS treated with whole-breast irradiation; wider margins don’t further reduce in-breast recurrence. - No sentinel node biopsy now: Routine SLNB is not recommended for pure DCIS treated with lumpectomy; it’s mainly considered if a mastectomy is planned or invasion is suspected.   **2) Radiation therapy (recommended)**  Young age and bilateral disease place her above “low-risk”; adjuvant RT halves ipsilateral events even in favorable DCIS.  Preferred regimen (per ASTRO WBI guidance and modern practice):   - Whole-breast irradiation to both breasts: 40.0–42.5 Gy in 15–16 fractions once daily (3 weeks). - Tumor-bed boost to each lumpectomy cavity (because she is ≤50 y): 10–16 Gy in 4–8 fractions. The randomized BIG 3-07/TROG 07.01 trial in non-low-risk DCIS showed a boost further reduces local recurrence (at the cost of some increased toxicity). - Planning/technique notes: Use heart-sparing (DIBH) for the left breast; meet standard heart/lung constraints. Either treat breasts concurrently or sequentially based on planning dosimetry and skin tolerance. (ASTRO endorses hypofractionation for WBI; DIBH is standard to lower mean heart dose.)   Acceptable alternative (center experience dependent):   - Ultra-hypofractionated WBI 26 Gy in 5 fractions over 1 week, with a sequential boost ~10–12.5 Gy in 4–5 fractions to each cavity (supported by FAST-Forward adoption data and real-world safety reports; many centers apply this to DCIS). Discuss pros/cons vs 3-week WBI.   Timing: Starting RT within ~12–16 weeks of surgery is acceptable.  **3) Endocrine therapy (recommended)**  Adjuvant endocrine therapy after BCS ± RT reduces both ipsilateral and contralateral events in ER-positive DCIS.  Standard option (best evidence for premenopausal DCIS):   - Tamoxifen 20 mg orally once daily for 5 years. Start after RT (or concurrently if preferred). Counsel on VTE risk, hot flashes, and endometrial symptoms; advise effective non-hormonal contraception (tamoxifen is teratogenic).   Lower-dose alternative (if tolerability is a concern):   - Tamoxifen 5 mg daily for 3 years, which showed durable 10-year reduction in breast events with fewer side effects in TAM-01 (many participants had DCIS). (Not yet universally standard, but reasonable in shared decision-making.)   **4) Systemic chemotherapy / biologics**   - Not indicated for pure DCIS.   **5) Genetic testing (recommended)**  She clearly meets contemporary criteria for germline testing: age ≤65 at diagnosis (age 38) and bilateral primary disease. Offer multigene panel centered on BRCA1/2, PALB2 (and, where appropriate, CHEK2, ATM, TP53, PTEN, CDH1), with pre- and post-test counseling. Results can influence surveillance and prophylactic options.  (NCCN patient materials also explicitly include DCIS within “breast cancer” for genetics discussions.).  **6) Follow-up**   - Clinical exam every 6–12 months for 5 years, then annually; annual mammography of both breasts after BCS/RT. Consider adding MRI if a pathogenic variant is found or if risk remains high.   **One possible, concrete plan to implement now**   1. No re-excision; no SLNB. 2. Radiation: Plan bilateral WBI 40.05 Gy in 15 fractions to each breast with DIBH for the left, followed by a boost 12.5 Gy in 5 fractions to each tumor bed. 3. Endocrine: Begin tamoxifen 20 mg daily for 5 years after RT; if side effects are limiting, discuss 5 mg daily for 3 years as an alternative. Use non-hormonal contraception; review VTE/endometrial warning signs. 4. Systemic chemo: none. 5. Genetics: Refer for germline testing (ASCO–SSO 2024 criteria met); test at minimum BRCA1/2 with panel expansion per counselor. | For a 38-year-old premenopausal woman with bilateral, estrogen and progesterone receptor–positive ductal carcinoma in situ (DCIS), status post bilateral breast-conserving tumorectomy with clear margins (R0, ≥4 mm), and ECOG 0, the following evidence-based management is recommended:  **Further surgical treatment:**  No additional surgery is indicated, as both lesions were excised with negative margins ≥2 mm, which is considered adequate by current standards. Sentinel lymph node biopsy is not recommended in DCIS treated with breast-conserving surgery unless future mastectomy is anticipated.  **Radiation therapy:**  Adjuvant whole-breast radiation therapy is standard after breast-conserving surgery for DCIS to reduce the risk of ipsilateral breast tumor recurrence, including both invasive and non-invasive events. This recommendation is supported by multiple randomized trials and is reflected in the current guidelines from the American College of Radiology and the National Comprehensive Cancer Network. Hypofractionated regimens (e.g., 40–42.5 Gy in 15–16 fractions) are commonly used, but the specific regimen should be tailored to institutional protocols and patient preference.  **Endocrine treatment:**  Adjuvant endocrine therapy (tamoxifen 20 mg daily for 5 years) is recommended for premenopausal women with hormone receptor–positive DCIS after breast-conserving surgery, as it reduces the risk of both ipsilateral and contralateral breast events. This is supported by the NSABP B-24 trial and is endorsed as an option in the guidelines from the American College of Radiology and the National Comprehensive Cancer Network. The decision should be individualized after discussing risks and benefits, as the absolute benefit is modest in patients with negative margins.  **Systemic (chemotherapy or targeted) treatment:**  No systemic chemotherapy or HER2-targeted therapy is indicated for pure DCIS in the absence of invasive disease or HER2 overexpression.  **Genetic testing:**  Given the young age at diagnosis (38 years) and bilateral disease, genetic counseling and consideration of germline testing for hereditary breast cancer syndromes (e.g., BRCA1/2) is appropriate, even though the family history is not strongly suggestive. This is consistent with current risk assessment practices.  **In summary:**  • No further surgery  • Whole-breast radiation therapy  • Tamoxifen 20 mg daily for 5 years  • No chemotherapy  • Genetic counseling/testing is appropriate |

| **PATIENT CASE 18** |  | |  | |  |  |  |  |
| --- | --- | --- | --- | --- | --- | --- | --- | --- |
|  |  | |  | |  |  |  |  |
| **How difficult or easy was the patient case?** | **very easy** | | **easy** | | **moderate** | **difficult** | **very difficult** | **Score** |
| **Case 18** | **1** | | **2** | | **3** | **4** | **5** |  |
|  |  | |  | |  |  |  |  |
| **The therapy concept is safe.** | **strongly disagree** | | **disagree** | | **neither agree nor disagree** | **agree** | **strongly agree** | **Score** |
| **Therapy Concept 1** | **1** | | **2** | | **3** | **4** | **5** |  |
| **Therapy Concept 2** | **1** | | **2** | | **3** | **4** | **5** |  |
| **Therapy Concept 3** | **1** | | **2** | | **3** | **4** | **5** |  |
|  |  | |  | |  |  |  |  |
| **The therapy concept is in line with the current guidelines.** | **strongly disagree** | | **disagree** | | **neither agree nor disagree** | **agree** | **strongly agree** | **Score** |
| **Therapy Concept 1** | **1** | | **2** | | **3** | **4** | **5** |  |
| **Therapy Concept 2** | **1** | | **2** | | **3** | **4** | **5** |  |
| **Therapy Concept 3** | **1** | | **2** | | **3** | **4** | **5** |  |
|  |  | |  | |  |  |  |  |
| **The therapy concept is medically adequate.** | **strongly disagree** | | **disagree** | | **neither agree nor disagree** | **agree** | **strongly agree** | **Score** |
| **Therapy Concept 1** | **1** | | **2** | | **3** | **4** | **5** |  |
| **Therapy Concept 2** | **1** | | **2** | | **3** | **4** | **5** |  |
| **Therapy Concept 3** | **1** | | **2** | | **3** | **4** | **5** |  |
|  |  | |  | |  |  |  |  |
| **The therapy concept is complete.** | **strongly disagree** | | **disagree** | | **neither agree nor disagree** | **agree** | **strongly agree** | **Score** |
| **Therapy Concept 1** | **1** | | **2** | | **3** | **4** | **5** |  |
| **Therapy Concept 2** | **1** | | **2** | | **3** | **4** | **5** |  |
| **Therapy Concept 3** | **1** | | **2** | | **3** | **4** | **5** |  |
|  |  | |  | |  |  |  |  |
| **The therapy concept is of high quality.** | **strongly disagree** | | **disagree** | | **neither agree nor disagree** | **agree** | **strongly agree** | **Score** |
| **Therapy Concept 1** | **1** | | **2** | | **3** | **4** | **5** |  |
| **Therapy Concept 2** | **1** | | **2** | | **3** | **4** | **5** |  |
| **Therapy Concept 3** | **1** | | **2** | | **3** | **4** | **5** |  |
|  |  | |  | |  |  |  |  |
| **The reasoning behind the treatment decision is logical.** | **strongly disagree** | | **disagree** | | **neither agree nor disagree** | **agree** | **strongly agree** | **Score** |
| **Therapy Concept 1** | **1** | | **2** | | **3** | **4** | **5** |  |
| **Therapy Concept 2** | **1** | | **2** | | **3** | **4** | **5** |  |
| **Therapy Concept 3** | **1** | | **2** | | **3** | **4** | **5** |  |
|  |  | |  | |  |  |  |  |
| **Please rank the therapy concept according to your preference.** | | **Ranking (1^st^, 2^nd^, 3^rd^)** | |  |  |  |  |  |
| **Therapy Concept 1** | |  | |  |  |  |  |  |
| **Therapy Concept 2** | |  | |  |  |  |  |  |
| **Therapy Concept 3** | |  | |  |  |  |  |  |

**Patient 19**

How should the following ductal carcinoma in situ patient be treated based on the most relevant and current international literature?

Patient information:

Age: 72

Menopause status: postmenopausal

ECOG: 1

Previous illness: Chronic lymphocytic leukemia Stadium A

Previous surgical treatment: Total shoulder arthroplasty on the left side

Birth history: 1 vaginal birth at the age of 22

Oncological family history: Father with colon cancer at age 55

The patient underwent the following surgical treatment: Breast-conserving tumorectomy on the left side

Following the surgical treatment, the following TNM-classification, resection margin, histological classification and immunohistology were identified:

TNM: pTis (size of lesion 1.5 cm)

Resection margin: R0, with minimal resection margin of 0.01 mm

Estrogen receptor (ER): 100%

Progesterone receptor (PR): 100%

Please provide a step-by-step treatment recommendation with regard to further surgical treatment, endocrine treatment, systemic treatment and radiation therapy taking the given patient information in consideration.

If endocrine treatment is advisable, please provide a suitable treatment regimen.

If systemic treatment is advisable, please provide a suitable treatment regimen.

If radiation therapy is advisable, please provide a suitable treatment regimen.

Based on the oncological family history, please state whether genetic testing should be performed or not.

| **Patient 19 Treatment Concepts** |  |  |
| --- | --- | --- |
| **Concept 1** | **Concept 2** | **Concept 3** |
| **Treatment Recommendation for Ductal Carcinoma In Situ (DCIS)**  **1. Further Surgical Treatment**  - No further surgical treatment is required as the patient has undergone breast-conserving tumorectomy with clear margins (R0). However, the minimal resection margin of 0.01 mm is narrow, and this may increase the risk of local recurrence. This should be considered when evaluating the need for adjuvant therapies.  **2. Endocrine Treatment**  - Endocrine therapy is recommended due to the strong hormone receptor positivity (ER: 100%, PR: 100%) and the postmenopausal status of the patient.  Recommended regimen:  - Aromatase inhibitor (AI): Anastrozole 1 mg orally once daily or Letrozole 2.5 mg orally once daily for 5 years.  - Alternatively, Tamoxifen 20 mg orally once daily for 5 years can be considered if aromatase inhibitors are contraindicated or not tolerated.  - Aromatase inhibitors are preferred in postmenopausal women due to their superior efficacy in reducing recurrence risk compared to Tamoxifen.  **3. Systemic Treatment**  - Systemic chemotherapy is not indicated for DCIS, as it is a non-invasive lesion without evidence of metastatic potential.  **4. Radiation Therapy**  - Radiation therapy is recommended to reduce the risk of local recurrence, especially given the narrow resection margin (0.01 mm) and the patient's ECOG status of 1, which indicates good functional status.  - Recommended regimen:  - Whole-breast irradiation (WBI): 40-50 Gy in 15-25 fractions over 3-5 weeks.  - Consider a boost dose of 10-16 Gy to the tumor bed due to the narrow margin, which increases the risk of recurrence.  **5. Genetic Testing**  - Based on the oncological family history (father with colon cancer at age 55), genetic testing is not routinely indicated unless there is a suspicion of hereditary cancer syndromes such as Lynch syndrome or hereditary breast and ovarian cancer syndrome (e.g., BRCA mutations).  - If the patient expresses concern or if there is additional family history of cancers, genetic counseling may be considered to assess the need for testing.  **Summary of Recommendations**  - No further surgery is required.  - Endocrine therapy: Aromatase inhibitor (preferred) or Tamoxifen for 5 years.  - Radiation therapy: Whole-breast irradiation with a possible boost to the tumor bed.  - No systemic chemotherapy is indicated.  - Genetic testing is not routinely recommended but may be considered based on further family history or patient preference. | Below is a practical, guideline-concordant plan for a 72-year-old, postmenopausal woman with ER/PR-positive DCIS (1.5 cm) treated with breast-conserving tumorectomy, pathologic stage pTis, R0 but very close margin (0.01 mm), ECOG 1, comorbidity: CLL stage A.  **1) Further surgical management (margins)**   - Why this matters: For DCIS treated with lumpectomy, an adequate negative margin is ≥ 2 mm when whole-breast irradiation (WBI) is planned; narrower margins carry higher local-recurrence risk. Re-excision is not *automatically* required in every case, but it should be strongly considered when margins are < 2 mm—especially when they are *extremely* close, as here (0.01 mm). - Recommendation: Discuss re-excision to obtain ≥ 2 mm radial clearance if it is technically straightforward and cosmetically acceptable. If re-excision is declined or would compromise cosmesis, proceed with WBI and a tumor-bed boost, as this partly mitigates close-margin risk. (Close margin alone is not an indication for mastectomy.)   **2) Radiation therapy**  Indication: After lumpectomy for DCIS, radiation significantly reduces ipsilateral breast events, including in “good-risk” disease; benefit persists to 15 years (no proven OS benefit). Given the very close margin, RT is advised.  Technique & dose options (choose one, based on local practice and preference):   - Standard hypofractionated WBI (preferred default): 40 Gy in 15 fractions (3 weeks) to the whole breast + boost to the tumor bed 10–16 Gy in 4–8 fractions (boost favored here because of the <2 mm margin). - Ultra-hypofractionated WBI (one-week) where available: 26 Gy in 5 daily fractions (1 week) to the whole breast + sequential boost ~10–12.5 Gy in 4–5 fractions (institutions differ on boost with 5-fraction WBI; data and guidance increasingly support carefully selected use).   Boost rationale: Contemporary randomized data in non-low-risk DCIS show a tumor-bed boost further lowers local recurrence, at the cost of slightly higher late breast induration/pain; with your 0.01 mm margin, the balance favors including a boost.  Not appropriate now: Partial-breast irradiation (PBI) is reasonable for selected small, favorable DCIS with adequate margins; it is not recommended with very close/positive margins. If a re-excision achieves ≥ 2–3 mm and other favorable features, PBI could be reconsidered.  **3) Endocrine therapy (risk-reduction)**  Indication: For ER-positive DCIS, adjuvant endocrine therapy reduces ipsilateral and contralateral breast events after BCS (with or without RT). It does not improve overall survival, so the choice should weigh absolute benefit against toxicity and comorbidities.  Postmenopausal options (choose one):   - Tamoxifen 20 mg orally once daily for 5 years (classic regimen; strongest long-term evidence in DCIS). - Anastrozole 1 mg orally once daily for 5 years (non-inferior disease-control vs tamoxifen in postmenopausal DCIS; side-effect profile differs—more arthralgia/bone loss, less thromboembolism/uterine effects). Consider if VTE risk is a concern.   Lower-dose alternative (for patients intolerant to standard dosing):   - Tamoxifen 5 mg daily for 3 years, which in a randomized trial halved breast events with fewer serious adverse effects over ~10-year follow-up; many guidelines now consider this a reasonable option when toxicity is a concern.   In this patient: At age 72 with CLL stage A (which may slightly increase infection/VTE concerns) and expected WBI, either tamoxifen 20 mg *or* anastrozole 1 mg is acceptable. If her VTE risk or uterine history makes tamoxifen less attractive, anastrozole is reasonable with bone protection (baseline DEXA, optimize calcium/vitamin D, consider bisphosphonate if osteopenic). If arthralgia/osteopenia are limiting, standard-dose tamoxifen or low-dose tamoxifen (5 mg × 3 y) are alternatives.  Timing: Start endocrine therapy after RT (typical) or concurrently if needed.  **4) Systemic (cytotoxic) therapy**   - Not indicated for pure DCIS. Chemotherapy has no role.   **5) Genetic testing**   - With DCIS at 72 and only one first-degree relative with colon cancer at 55 (no breast/ovarian/pancreatic/prostate pattern, no early-onset breast cancer, no male breast cancer), routine germline testing is not indicated under current NCCN/ESMO criteria. (Testing could be reconsidered if additional family history emerges.)   **Putting it all together (step-by-step)**   1. Re-excision discussion to achieve ≥ 2 mm margin (preferred), otherwise proceed as below. 2. Whole-breast irradiation after BCS:    - 40 Gy/15 fx (3 weeks) + boost 10–16 Gy (recommended given 0.01 mm margin), *or*    - 26 Gy/5 fx (1 week) + sequential boost ~10–12.5 Gy where practiced. 3. Endocrine therapy (choose one):    - Tamoxifen 20 mg daily × 5 y, *or* Anastrozole 1 mg daily × 5 y (with bone health measures), *or* Tamoxifen 5 mg daily × 3 y if toxicity limits standard dosing. Start after RT. 4. No chemotherapy. 5. No routine genetic testing based on current family history; revisit if new history appears.   **Notes tailored to this patient**   - Age 72 & ECOG 1: Favor short, hypofractionated RT to reduce burden; WBI 40/15 is widely standard; 26/5 is acceptable in many centers—confirm local experience with boost after 5-fraction WBI. - CLL (stage A): Not a contraindication to RT or endocrine therapy; if choosing tamoxifen, review personal VTE risk; if choosing anastrozole, optimize bone health. (General endocrine guidance as above.)   **Key sources**   - Margins (DCIS): SSO-ASTRO-ASCO consensus: ≥ 2 mm recommended; < 2 mm warrants individualized re-excision decision-making. NICE/ABS 2024 also advise discussing re-excision for < 2 mm. - RT benefit in DCIS: RTOG 9804 15-year follow-up—WBI significantly reduces ipsilateral events in “good-risk” DCIS. - Boost in DCIS: BIG 3-07/TROG 07.01—boost lowers recurrence in non-low-risk DCIS, with modestly higher late toxicity. - Hypofractionation: ASTRO WBI guideline (2018) endorses 40/15; modern data/guidance support 26/5 in selected settings; Ontario 2023 explicitly allows 26/5 for DCIS. - Endocrine therapy: NSABP B-24 (tamoxifen benefit); IBIS-II DCIS (anastrozole ≈ tamoxifen in postmenopause; differing toxicities); TAM-01 (tamoxifen 5 mg × 3 y effective). - Genetics: NCCN Genetic/Familial High-Risk Assessment—no testing criteria met with current history.   **Follow-up**  After completing local therapy (± endocrine), annual mammography of both breasts and routine clinical follow-up are appropriate; no special DCIS-specific lab imaging is needed. (Follow local protocols.) | For a 72-year-old postmenopausal woman with ER/PR-positive, pure DCIS (pTis, 1.5 cm), R0 resection with a minimal margin of 0.01 mm after breast-conserving tumorectomy, the following evidence-based management steps are recommended:  **1. Further Surgical Treatment:**  No further surgery is indicated, as the resection margin is negative (R0), even though the margin is minimal. The American College of Radiology and the National Comprehensive Cancer Network (NCCN) recommend no re-excision for negative margins, regardless of width, unless there are other high-risk features or patient preference.  **2. Radiation Therapy:**  Adjuvant whole-breast radiation therapy is generally recommended after breast-conserving surgery for DCIS to reduce the risk of ipsilateral breast tumor recurrence, as supported by multiple randomized trials and endorsed by the American College of Radiology and NCCN. Omission of radiation may be considered in select low-risk patients (older age, small, low/intermediate grade, wide margins), but this patient’s minimal margin (0.01 mm) does not meet typical criteria for omission. Standard regimens include conventional fractionation (e.g., 50 Gy in 25 fractions) or hypofractionation (e.g., 40 Gy in 15 fractions).  **3. Endocrine Treatment:**  Adjuvant endocrine therapy (e.g., tamoxifen 20 mg daily or an aromatase inhibitor for postmenopausal women) is recommended for risk reduction of ipsilateral and contralateral breast events in patients with ER-positive DCIS after breast-conserving surgery, as per the American College of Radiology and NCCN. The choice between tamoxifen and aromatase inhibitor should be individualized based on comorbidities and patient preference.  **4. Systemic (Cytotoxic) Therapy:**  No cytotoxic systemic therapy is indicated for pure DCIS, as there is no evidence of benefit.  **5. Genetic Testing:**  Genetic testing is not indicated based on this family history (single first-degree relative with colon cancer at age 55 and no breast/ovarian cancer), as per current risk stratification practices.  **In summary:**  No further surgery, adjuvant whole-breast radiation, adjuvant endocrine therapy (tamoxifen or aromatase inhibitor), no cytotoxic chemotherapy, and no genetic testing are recommended for this patient. |

| **PATIENT CASE 19** |  |  |  |  |  |  |
| --- | --- | --- | --- | --- | --- | --- |
|  |  |  |  |  |  |  |
| **How difficult or easy was the patient case?** | **very easy** | **easy** | **moderate** | **difficult** | **very difficult** | **Score** |
| **Case 19** | **1** | **2** | **3** | **4** | **5** |  |
|  |  |  |  |  |  |  |
| **The therapy concept is safe.** | **strongly disagree** | **disagree** | **neither agree nor disagree** | **agree** | **strongly agree** | **Score** |
| **Therapy Concept 1** | **1** | **2** | **3** | **4** | **5** |  |
| **Therapy Concept 2** | **1** | **2** | **3** | **4** | **5** |  |
| **Therapy Concept 3** | **1** | **2** | **3** | **4** | **5** |  |
|  |  |  |  |  |  |  |
| **The therapy concept is in line with the current guidelines.** | **strongly disagree** | **disagree** | **neither agree nor disagree** | **agree** | **strongly agree** | **Score** |
| **Therapy Concept 1** | **1** | **2** | **3** | **4** | **5** |  |
| **Therapy Concept 2** | **1** | **2** | **3** | **4** | **5** |  |
| **Therapy Concept 3** | **1** | **2** | **3** | **4** | **5** |  |
|  |  |  |  |  |  |  |
| **The therapy concept is medically adequate.** | **strongly disagree** | **disagree** | **neither agree nor disagree** | **agree** | **strongly agree** | **Score** |
| **Therapy Concept 1** | **1** | **2** | **3** | **4** | **5** |  |
| **Therapy Concept 2** | **1** | **2** | **3** | **4** | **5** |  |
| **Therapy Concept 3** | **1** | **2** | **3** | **4** | **5** |  |
|  |  |  |  |  |  |  |
| **The therapy concept is complete.** | **strongly disagree** | **disagree** | **neither agree nor disagree** | **agree** | **strongly agree** | **Score** |
| **Therapy Concept 1** | **1** | **2** | **3** | **4** | **5** |  |
| **Therapy Concept 2** | **1** | **2** | **3** | **4** | **5** |  |
| **Therapy Concept 3** | **1** | **2** | **3** | **4** | **5** |  |
|  |  |  |  |  |  |  |
| **The therapy concept is of high quality.** | **strongly disagree** | **disagree** | **neither agree nor disagree** | **agree** | **strongly agree** | **Score** |
| **Therapy Concept 1** | **1** | **2** | **3** | **4** | **5** |  |
| **Therapy Concept 2** | **1** | **2** | **3** | **4** | **5** |  |
| **Therapy Concept 3** | **1** | **2** | **3** | **4** | **5** |  |
|  |  |  |  |  |  |  |
| **The reasoning behind the treatment decision is logical.** | **strongly disagree** | **disagree** | **neither agree nor disagree** | **agree** | **strongly agree** | **Score** |
| **Therapy Concept 1** | **1** | **2** | **3** | **4** | **5** |  |
| **Therapy Concept 2** | **1** | **2** | **3** | **4** | **5** |  |
| **Therapy Concept 3** | **1** | **2** | **3** | **4** | **5** |  |
|  |  |  |  |  |  |  |
| **Please select your preferred therapy concept (one choice)** | **Score** |  |  |  |  |  |
| **Therapy Concept 1** |  |  |  |  |  |  |
| **Therapy Concept 2** |  |  |  |  |  |  |
| **Therapy Concept 3** |  |  |  |  |  |  |

**Patient 20**

How should the following breast cancer patient be treated based on the most relevant and current international literature?

Patient information:

Age: 36

Menopause status: premenopausal

ECOG: 0

Previous illness: None

Previous surgical treatment: None

Oncological family history: No cancer history

The patient did not undergo surgery so far.

Following the surgical biopsy and staging, the following information about the tumor is available:

TNM: cT4dpN+M0

Histological classification: NST, inflammatory breast cancer with lymphangiosis carcinomatosa

Grading: G3

Estrogen receptor (ER): 5%

Progesterone receptor (PR): 5%

Her2-status: Positive (ISH positive)

Ki-67-proliferation-index: 70%

Please provide a step-by-step treatment recommendation with regard to the necessity of neoadjuvant treatment, present or future surgical treatment, endocrine treatment, systemic treatment and radiation therapy taking the given patient information into consideration.

If endocrine treatment is advisable, please provide a suitable treatment regimen.

If systemic treatment is advisable, please provide a suitable treatment regimen.

If radiation therapy is advisable, please provide a suitable treatment regimen.

Based on the oncological family history, please state whether genetic testing should be performed or not.

| **Patient 20 Treatment Concepts** |  |  |
| --- | --- | --- |
| **Concept 1** | **Concept 2** | **Concept 3** |
| **Step-by-Step Treatment Recommendation for the Patient**  **1. Neoadjuvant Treatment**  Given the diagnosis of inflammatory breast cancer (cT4d), HER2-positive status, high Ki-67 index (70%), and premenopausal status, neoadjuvant systemic therapy is strongly recommended. This approach aims to:  - Reduce tumor burden and improve surgical outcomes.  - Assess response to therapy, which can guide adjuvant treatment decisions.  Recommended Neoadjuvant Regimen:  - Chemotherapy: A taxane-based and anthracycline-based regimen is preferred.  - Example: Dose-dense AC (doxorubicin + cyclophosphamide) for 4 cycles, followed by weekly paclitaxel for 12 weeks.  - HER2-targeted therapy: Dual HER2 blockade is recommended.  - Example: Trastuzumab + Pertuzumab initiated concurrently with paclitaxel and continued throughout the neoadjuvant phase.  **2. Surgical Treatment**  Following neoadjuvant therapy, the patient should undergo surgery to achieve local control:  - Type of Surgery: Modified radical mastectomy is the standard for inflammatory breast cancer due to its aggressive nature.  - Axillary Management: Axillary lymph node dissection (ALND) is indicated given the clinical nodal involvement (cN+).  **3. Adjuvant Systemic Treatment**  The choice of adjuvant therapy depends on the response to neoadjuvant treatment:  - HER2-targeted therapy: Continue trastuzumab for a total duration of 1 year. If residual disease is present post-surgery, consider trastuzumab emtansine (T-DM1) for 14 cycles.  - Endocrine therapy: Given the ER/PR positivity (5%), endocrine therapy is recommended post-surgery.  - Premenopausal regimen: Ovarian suppression (e.g., goserelin) combined with tamoxifen for 5–10 years.  **4. Radiation Therapy**  Radiation therapy is mandatory after surgery due to the inflammatory nature of the cancer and the high risk of locoregional recurrence:  - Target Areas: Chest wall and regional lymph nodes (axillary, supraclavicular, and internal mammary nodes).  - Regimen: Conventional fractionation (e.g., 50 Gy in 25 fractions over 5 weeks).  **5. Genetic Testing**  Although there is no family history of cancer, the patient’s young age (36 years) and diagnosis of aggressive breast cancer warrant genetic testing for hereditary breast cancer syndromes (e.g., BRCA1/2 mutations). This information may influence future treatment and surveillance strategies.  **Summary of Treatment Plan**  1. Neoadjuvant Therapy: Chemotherapy (AC → paclitaxel) + dual HER2 blockade (trastuzumab + pertuzumab).  2. Surgery: Modified radical mastectomy with ALND.  3. Adjuvant Therapy:  - HER2-targeted therapy: Continue trastuzumab or switch to T-DM1 if residual disease.  - Endocrine therapy: Ovarian suppression + tamoxifen.  4. Radiation Therapy: Chest wall and regional lymph nodes.  5. Genetic Testing: Recommended due to young age and aggressive tumor biology. | Here’s a practical, guideline-concordant plan for a 36-year-old, premenopausal woman with inflammatory breast cancer (IBC), cT4d pN+ M0, HER2-positive (ISH+), ER 5% / PR 5% (ER-low), G3, Ki-67 70%, ECOG 0, no prior surgery.  **1) Up-front priorities (before therapy starts)**   - Confirm full staging & baseline assessments   - Whole-body PET/CT (or CT chest/abdomen/pelvis + bone scan) and breast MRI if not already done (IBC has a high occult metastatic risk). Many IBC pathways recommend PET/CT for initial staging.   - Baseline cardiac function (echo or MUGA); repeat about every 3 months while on anti-HER2 therapy.   - Fertility counseling and rapid-access referral (oocyte/embryo cryopreservation). GnRH agonist (e.g., goserelin) during chemotherapy to reduce gonadotoxicity can be considered. - Genetic testing (germline panel): Yes. Age 36 meets contemporary criteria regardless of family history; offer at least BRCA1/2 and PALB2, typically via a multigene panel (consider TP53 in very young women).   **2) Neoadjuvant systemic therapy (NAST) — required in IBC**  Why: IBC is treated with trimodality therapy beginning with neoadjuvant systemic treatment to improve operability and outcomes. For HER2-positive disease, dual HER2 blockade + chemotherapy is standard.  Preferred regimen (evidence-based, anthracycline-free)  TCHP × 6 cycles q3w   - Docetaxel 75 mg/m² IV day 1 - Carboplatin AUC 6 day 1 - Pertuzumab 840 mg load → 420 mg q3w - Trastuzumab 8 mg/kg load → 6 mg/kg q3w   Rationale: High pCR rates with docetaxel + HP, and no survival advantage from adding anthracyclines when dual HER2 blockade is used (TRAIN-2), with less cardiotoxicity.  Acceptable alternative (if carboplatin isn’t suitable): Dose-dense AC → weekly paclitaxel + HP (e.g., ddAC x4 then paclitaxel 80 mg/m² weekly x12 with trastuzumab/pertuzumab), recognizing TRAIN-2 shows no clear benefit to the anthracycline component.  Notes   - Primary G-CSF prophylaxis is reasonable given FN risk with TCHP. - Start ovarian suppression (if using for fertility protection) before chemo.   **3) Surgery after NAST (assuming no progression)**  For non-metastatic IBC, breast conservation is not standard.   - Modified radical mastectomy (MRM) with level I–II axillary lymph-node dissection (ALND) on the involved side. Sentinel-node biopsy is not recommended in IBC. Immediate reconstruction is generally discouraged; plan delayed reconstruction after radiation.   Pathology should document residual disease status (pCR vs residual) and nodal response to guide adjuvant therapy.  **4) Post-operative radiation (PMRT) — mandatory in IBC**  Targets: chest wall plus all regional nodes (levels I–III axilla, infraclavicular/supraclavicular, internal mammary nodes). Use skin bolus strategy to ensure adequate skin dose given dermal lymphatic involvement.  Typical dosing (choose per center protocols and response):   - 50–50.4 Gy in 25–28 fx to chest wall and nodal basins + boost 10–16 Gy to the chest wall/skin involved areas (total 60–66 Gy). Daily or every-other-day bolus per institutional practice. - Selected centers use hyperfractionation for poor responders/high-risk features (e.g., 51 Gy BID + 15 Gy boost).   Sequencing with systemic therapy: PMRT is given after surgery; it can be delivered concurrently with trastuzumab/pertuzumab and is commonly given with T-DM1 as well (monitor for dermatitis). (Practice pattern per guidelines/centers.)  **5) Anti-HER2 therapy after surgery — depends on pathologic response**  A) If pCR (ypT0/is ypN0)   - Continue trastuzumab ± pertuzumab to complete 1 total year of anti-HER2 therapy (counting neoadjuvant doses).   - Continuing pertuzumab is reasonable in patients node-positive at diagnosis, supported by APHINITY, which shows sustained iDFS (and now OS) benefit in node-positive early HER2+ disease.   B) If residual invasive disease in breast and/or nodes   - Switch to T-DM1 3.6 mg/kg IV q3w × 14 cycles (post-neoadjuvant standard; improves iDFS and OS vs trastuzumab).   Extended adjuvant neratinib?   - May be considered for HR-positive patients at high risk who completed trastuzumab-based adjuvant therapy (not T-DM1) within the past year, acknowledging diarrhea risk and uncertain value in ER-low (5%) tumors. Not routine in this case and generally not used after T-DM1.   **6) Endocrine therapy (because ER/PR 5% = ER-low)**   - What guidelines say: ER ≥1% is reported as ER-positive, but benefit is modest/uncertain when ER is 1–10%; treatment should be individualized. - Recommendation here: discuss pros/cons; given very high-risk IBC biology, many teams offer endocrine therapy despite ER-low.   Reasonable regimens for a premenopausal patient:   1. Ovarian function suppression (OFS) + tamoxifen 20 mg daily for 5 years (start after chemo; can overlap with anti-HER2 therapy). 2. OFS + aromatase inhibitor (e.g., letrozole 2.5 mg daily) for 5 years if reliable ovarian suppression and bone health are managed. SOFT/TEXT show OFS+AI improves DFS in high-risk premenopausal HR+ disease (data largely HER2-negative, but informs approach).  - When to extend beyond 5 years: extension to 7–10 years can be considered for very high risk if well-tolerated, but incremental benefit in ER-low is uncertain. - Supportive care: baseline DEXA, vitamin D/calcium; consider bisphosphonate/denosumab per local policy if AI+OFS.   **7) Putting it all together — step-by-step**   1. Today: finalize staging; baseline echo; urgent fertility consult; place port; labs/pregnancy test; vaccinations if needed. 2. Start neoadjuvant TCHP × 6 q3w with G-CSF support; echo q3 months. Reassess by exam/imaging mid-therapy. 3. Surgery: MRM + ALND (no SLNB; no immediate reconstruction). 4. Adjuvant path-directed:    - pCR → continue H±P to 1 year total anti-HER2; PMRT as below; start endocrine therapy if chosen.    - Residual disease → T-DM1 × 14; PMRT; start endocrine therapy with T-DM1.    - Consider neratinib only if high-risk HR+ and completed trastuzumab-based adjuvant therapy (not after T-DM1); discuss diarrhea prophylaxis if chosen. 5. Radiation: chest wall + levels I–III + supraclavicular/infraclavicular + internal mammary nodes. Dose 50–50.4 Gy/25–28 fx + 10–16 Gy boost to involved skin (total 60–66 Gy). Consider BID escalation for poor responders. Use bolus strategy per center protocols. 6. Follow-up & monitoring: cardiac imaging during anti-HER2 therapy; manage treatment toxicities (diarrhea with neratinib if used; neuropathy with taxanes; cytopenias; skin during PMRT); survivorship, lymphedema prevention, and psychosocial support.   **Why these choices fit this patient**   - IBC mandates neoadjuvant systemic therapy → mastectomy/ALND → PMRT. - HER2-positive: highest pCR rates with dual anti-HER2 + chemo; omitting anthracyclines is supported (TRAIN-2). - Node-positive at diagnosis: if pCR, continuing pertuzumab with trastuzumab to complete a year is favored by APHINITY (benefit concentrated in node-positive pts). If residual disease, T-DM1 is superior (KATHERINE). - ER-low (5%): endocrine therapy benefit is uncertain but may be offered; if used in this very high-risk scenario, OFS + tamoxifen or OFS + AI are appropriate premenopausal options. - Age 36: germline testing is appropriate independent of pedigree.   **Quick regimen templates you can copy into orders**   - NAST (preferred): *TCHP q3w × 6* Docetaxel 75 mg/m² D1 + Carboplatin AUC 6 D1 + Trastuzumab 8→6 mg/kg q3w + Pertuzumab 840→420 mg q3w. - If residual disease post-op: *T-DM1 3.6 mg/kg IV q3w × 14* (start ~3–6 weeks after surgery or after PMRT per local sequence; many centers give concurrently with PMRT). - If pCR post-op: continue *trastuzumab ± pertuzumab q3w* to 1 year total anti-HER2. - Endocrine therapy (if chosen): OFS (goserelin 3.6 mg q28d or 10.8 mg q12w) + tamoxifen 20 mg daily × 5 y (or OFS + letrozole 2.5 mg daily × 5 y with bone protection). - Radiation: chest wall + RNI (IMNs included), 50–50.4 Gy/25–28 fx, boost 10–16 Gy to involved skin; consider BID dose-escalation in poor responders.   **Citations (key sources)**   - NCCN Patients (IBC 2025) – trimodality approach; mastectomy + ALND; PMRT. - TRAIN-2 – no added benefit of anthracyclines with dual HER2 blockade; supports TCHP/anthracycline-free. - NeoSphere / TRYPHAENA – dual HER2 blockade improves pCR; TCHP dosing used widely. - KATHERINE – adjuvant T-DM1 improves iDFS & OS for residual disease after NAST. - APHINITY – adjuvant pertuzumab benefit, particularly node-positive (8- to 10-year data incl. OS). - ASCO/CAP 2020 ER/PR – ER-low (1–10%): limited endocrine benefit; individualized decisions. - SOFT/TEXT long-term – OFS + AI or tamoxifen in high-risk premenopausal HR+ disease. - ASBrS / ASCO – SLNB not indicated in IBC. - PMRT in IBC – comprehensive RNI incl. IMNs, dose 60–66 Gy with boost/bolus; BID escalation in select cases. - Cardiac monitoring – echo at baseline and during anti-HER2 (commonly q3 months). - Germline testing – 2024 ASCO–SSO guideline supports broad testing; age 36 qualifies.   **Bottom line for this patient**   - Start neoadjuvant TCHP × 6, then MRM + ALND, then PMRT to chest wall + all nodal basins. - If pCR: finish 1 year of anti-HER2 (continue H±P, especially given node-positive presentation), and discuss endocrine therapy (OFS + tamoxifen or OFS + AI). - If residual disease: T-DM1 × 14, PMRT, and endocrine therapy as above. - Do germline testing now; fertility preservation discussion immediately.   This plan aligns with the most current international evidence and consensus for HER2-positive IBC in a young, premenopausal patient. | This patient has locally advanced, inflammatory, HER2-positive, hormone receptor–low (ER 5%, PR 5%), grade 3 breast cancer, with nodal involvement and no distant metastases. The standard of care is as follows:  **Neoadjuvant systemic therapy:**  Mandatory for inflammatory breast cancer and for HER2-positive, node-positive disease. The American Society of Clinical Oncology (ASCO) recommends neoadjuvant chemotherapy combined with dual anti-HER2 therapy (trastuzumab and pertuzumab) for such patients. A typical regimen is anthracycline- and taxane-based chemotherapy (e.g., dose-dense doxorubicin/cyclophosphamide followed by paclitaxel) with concurrent trastuzumab and pertuzumab during the taxane phase. Trastuzumab and pertuzumab should not be given concurrently with anthracyclines due to cardiac toxicity risk.  **Surgical treatment (should follow neoadjuvant therapy):**  For inflammatory breast cancer, the standard is modified radical mastectomy with axillary lymph node dissection. Breast-conserving surgery is contraindicated in inflammatory breast cancer.  **Radiation therapy:**  Indicated post-mastectomy for all patients with inflammatory breast cancer, regardless of response, targeting the chest wall and regional lymphatics.  **Endocrine therapy:**  Not routinely indicated for ER/PR expression <10%. For ER/PR ≥1%, some guidelines consider endocrine therapy, but the benefit is likely minimal in this case. If used, tamoxifen is preferred in premenopausal women, but the primary focus should remain on HER2-targeted and cytotoxic therapy.  **Genetic testing:**  Not routinely indicated in the absence of a family history or other risk factors, but may be considered due to the young age (<40 years) at diagnosis, as per standard risk assessment practices.  **In summary:**  The patient should receive neoadjuvant anthracycline- and taxane-based chemotherapy with trastuzumab and pertuzumab, followed by modified radical mastectomy, postmastectomy radiation, and consideration of endocrine therapy only if ER/PR expression is deemed clinically significant. Genetic testing may be considered due to young age. All recommendations are supported by the ASCO guidelines and current international literature. |

| **PATIENT CASE 20** |  | |  | |  |  |  |  |
| --- | --- | --- | --- | --- | --- | --- | --- | --- |
|  |  | |  | |  |  |  |  |
| **How difficult or easy was the patient case?** | **very easy** | | **easy** | | **moderate** | **difficult** | **very difficult** | **Score** |
| **Case 20** | **1** | | **2** | | **3** | **4** | **5** |  |
|  |  | |  | |  |  |  |  |
| **The therapy concept is safe.** | **strongly disagree** | | **disagree** | | **neither agree nor disagree** | **agree** | **strongly agree** | **Score** |
| **Therapy Concept 1** | **1** | | **2** | | **3** | **4** | **5** |  |
| **Therapy Concept 2** | **1** | | **2** | | **3** | **4** | **5** |  |
| **Therapy Concept 3** | **1** | | **2** | | **3** | **4** | **5** |  |
|  |  | |  | |  |  |  |  |
| **The therapy concept is in line with the current guidelines.** | **strongly disagree** | | **disagree** | | **neither agree nor disagree** | **agree** | **strongly agree** | **Score** |
| **Therapy Concept 1** | **1** | | **2** | | **3** | **4** | **5** |  |
| **Therapy Concept 2** | **1** | | **2** | | **3** | **4** | **5** |  |
| **Therapy Concept 3** | **1** | | **2** | | **3** | **4** | **5** |  |
|  |  | |  | |  |  |  |  |
| **The therapy concept is medically adequate.** | **strongly disagree** | | **disagree** | | **neither agree nor disagree** | **agree** | **strongly agree** | **Score** |
| **Therapy Concept 1** | **1** | | **2** | | **3** | **4** | **5** |  |
| **Therapy Concept 2** | **1** | | **2** | | **3** | **4** | **5** |  |
| **Therapy Concept 3** | **1** | | **2** | | **3** | **4** | **5** |  |
|  |  | |  | |  |  |  |  |
| **The therapy concept is complete.** | **strongly disagree** | | **disagree** | | **neither agree nor disagree** | **agree** | **strongly agree** | **Score** |
| **Therapy Concept 1** | **1** | | **2** | | **3** | **4** | **5** |  |
| **Therapy Concept 2** | **1** | | **2** | | **3** | **4** | **5** |  |
| **Therapy Concept 3** | **1** | | **2** | | **3** | **4** | **5** |  |
|  |  | |  | |  |  |  |  |
| **The therapy concept is of high quality.** | **strongly disagree** | | **disagree** | | **neither agree nor disagree** | **agree** | **strongly agree** | **Score** |
| **Therapy Concept 1** | **1** | | **2** | | **3** | **4** | **5** |  |
| **Therapy Concept 2** | **1** | | **2** | | **3** | **4** | **5** |  |
| **Therapy Concept 3** | **1** | | **2** | | **3** | **4** | **5** |  |
|  |  | |  | |  |  |  |  |
| **The reasoning behind the treatment decision is logical.** | **strongly disagree** | | **disagree** | | **neither agree nor disagree** | **agree** | **strongly agree** | **Score** |
| **Therapy Concept 1** | **1** | | **2** | | **3** | **4** | **5** |  |
| **Therapy Concept 2** | **1** | | **2** | | **3** | **4** | **5** |  |
| **Therapy Concept 3** | **1** | | **2** | | **3** | **4** | **5** |  |
|  |  | |  | |  |  |  |  |
| **Please rank the therapy concept according to your preference.** | | **Ranking (1^st^, 2^nd^, 3^rd^)** | |  |  |  |  |  |
| **Therapy Concept 1** | |  | |  |  |  |  |  |
| **Therapy Concept 2** | |  | |  |  |  |  |  |
| **Therapy Concept 3** | |  | |  |  |  |  |  |
